# Vision Evaluation Tools for Adults With Acquired Brain Injury: A Scoping Review

**DOI:** 10.1177/00084174211042955

**Published:** 2021-10-18

**Authors:** Camille Dubé, Yu Jin, Brienne G. Powers, Ginny Li, Amélie Labelle, Meghan S. Rivers, Ivy M. Gumboc, André E. Bussières

**Keywords:** Rehabilitation, Screening, Stroke, Vision disorders, Visual-perceptual, Accident vasculaire cérébral, déficits visuels‌, dépistage, réadaptation

## Abstract

**Background.** Unrecognized visual deficits (VDs) following an acquired brain injury (ABI) may impact clients’ rehabilitation. Little is known about evaluation tools used in vision rehabilitation. **Purpose.** To systematically explore the literature describing evaluation tools used for VD on adults with ABI. **Method.** Using a scoping review methodology, we searched in MEDLINE(Ovid), Embase, CINAHL, PsycINFO, and the grey literature from inception to 2020. Quantitative and thematic analyses were performed. **Findings.** Of the 83 studies reporting on 86 evaluation tools, 47% used multiple tools to assess VD. Tools were mostly used by occupational therapists and psychologists to evaluate intermediate, intermediate to high, and high-level visual skills. Clinicians tend to select specific tools that focus on different levels of the hierarchy of visual skills. **Implications.** Future research should investigate the optimal timeframe for assessment of VD and the psychometric properties of tools to ensure comprehensive VD evaluation.

## Abrégé

**Description.** Les déficits visuels (DV) non reconnus après une lésion cérébrale acquise (LCA) peuvent avoir un impact sur la réadaptation des clients. On sait peu de choses sur les outils d'évaluation utilisés en réadaptation visuelle. **But.** Explorer systématiquement la littérature décrivant les outils d'évaluation utilisés pour les déficits visuels chez les adultes atteints de lésions cérébrales acquises. **Méthodologie.** En utilisant une méthodologie de revue de portée, nous avons effectué des recherches dans MEDLINE(Ovid), Embase, CINAHL, PsycINFO et la littérature grise, depuis leur date de création jusqu'à 2020. Des analyses quantitatives et thématiques ont été réalisées. **Résultats.** Sur les 83 études rapportant 86 outils d'évaluation, 47% ont utilisé plusieurs outils pour évaluer la DV. Les outils étaient surtout utilisés par les ergothérapeutes et les psychologues pour évaluer les compétences visuelles de niveau intermédiaire, intermédiaire à élevé et élevé. Les cliniciens ont tendance à sélectionner des outils spécifiques qui se concentrent sur différents niveaux de la hiérarchie des compétences visuelles. **Conséquences.** Les recherches futures devraient porter sur l'échéancier optimal d'évaluation des DV et sur les propriétés psychométriques des outils afin de garantir une évaluation complète des DV.

## Introduction

Over 1.5 million Canadians live with acquired brain injury (ABI) (Brain Injury Association of Canada [BIAC], 2014), a condition associated with significant dysfunction when not well managed (Roberts et al., 2016). Visual deficits (VDs) following ABI are highly prevalent (Roberts et al., 2016). While the precise prevalence of VD following ABI in Canada is unknown, a 2008 study on clients with ABI from the United States suggested that about 50% of subjects experienced VD (Ciuffreda et al., 2008). Importantly, VD may increase the risk of falls, hip fractures, depression, social isolation, difficulty reading, and utilizing community services (Roberts et al., 2016). Despite the high prevalence of VD following ABI, rehabilitation professionals report a lack of knowledge as to when they should assess clients for VD following ABI, and what tools and assessments should be used (Winner et al., 2014).

According to Brain Injury Canada (BIC) (2019), ABI refers to an injury to the brain that occurs after birth, which is not hereditary, congenital, or degenerative and may be categorized into two types: traumatic and non-traumatic. A traumatic brain injury (TBI) is caused by an external force (e.g., a fall, sports injury, motor vehicle crash), while a non-traumatic brain injury is an internal injury to the brain itself (e.g., cerebral vascular accident, encephalitis, toxic substance exposure) (BIC, 2019; Ciuffreda, 2007; Diamond, 2009). Individuals can experience the effects of ABI immediately after the injury and symptoms may change over time. Published studies have estimated the annual incidence of severe TBI at 11.4 per 100,000 and of mild TBI at 600 per 100,000 (Cassidy et al., 2004; Zygun et al., 2005), with a disproportionately high occurrence in males aged 15–24 years (Pickett et al., 2001). Krueger et al. (2015) estimated that 405,000 individuals experienced the effects of stroke in Canada in 2013 (prevalence of 1.15%), with the prevalence increasing with age (Hodgson, 1998).

Vision is a primary sense with complex neurological organization. The visual pathway begins with the retina converting the image formed by the light into nerve impulses through the optic nerve. Axons of the optic nerve continue ipsilateral to the optic tracts at the optic chiasm. Most of the nerve fibers in the optic tract project to the lateral geniculate nucleus (LGN) of the thalamus. From the LGN, the visual stimuli are relayed to the primary visual cortex and secondary visual cortex located in the occipital lobe where the brain begins to reconstitute the image and where conscious visual perception takes place (Dreher & Robinson, 1991; Dubuc, 2020). The analysis of visual stimuli continues through the ventral pathway, which extends to the temporal lobe and may be involved in recognizing objects, and the dorsal pathway, which projects to the parietal lobe and appears to be essential for locating objects. Problems occurring anywhere along the visual pathway may lead to VD. For instance, disruption of cranial nerve function or disruption of central neural control can cause disruption of oculomotor control (Warren, 1993).

Several terms referring to VD are used interchangeably in the literature across disciplines, including neuro-visual deficits, visual problems, and vision impairments. Likewise, the definition of VD varies in research. The boundaries between constructs of visual perception, visual function, and functional vision are poorly defined (Lieberman, 1984; Roberts et al., 2016; Warren, 1993). The Warren’s (1993) hierarchical model for evaluation and treatment of visual perceptual dysfunction in adults with ABI helps guide clinical reasoning and practice in low vision rehabilitation settings (Roberts et al., 2016).

The Warren model categorizes visual skills into foundation, intermediate, and high levels. Foundation skills are responsible for the reception of the visual stimuli which include oculomotor control, visual fields, and acuity (Cavanagh, 2011; Lieberman, 1984; Warren, 1993). Intermediate skills are involved with identification and recognition of objects in space (e.g., visual scanning and attention). High-level visual perception skills enable the mental manipulation of visual information and the ability to integrate it with other sensory information to solve problems and make decisions. These skills are arranged in a hierarchy with function of high-level skills dependent on adequate function of foundation-level skills. Thus, Warren (1993) proposed that assessment and intervention should be carried out following this model, with foundation skills being screened and treated first before addressing intermediate and high-level skills. ABI can result in one or multiple VD, often affecting all three levels of visual skills (Armstrong, 2018; Roberts et al., 2016; Warren, 1993).

Rehabilitation settings include acute care hospitals, inpatient acute, sub-acute or outpatient rehabilitation facilities, skilled nursing or long-term care facilities, rehabilitation centers, specialty clinics, community-based programs, and private practice (American Occupational Therapy Association, 2016; Chonsky, 2012).

Evaluation tools used to evaluate VD in clients with ABI serve three purposes. Diagnostic tools are used by vision specialists (e.g., neuro-optometrists, ophthalmologists) to give or confirm a diagnosis (Freeman et al., 2007). Screening tools are used by clinicians to identify individuals with VD and do not require specialized equipment or training (Pillay et al., 2016). Assessment tools are comprehensive tests used by clinicians to evaluate capacities, build intervention plans, monitor changes, and document outcomes (Cooke et al., 2006; Radomski et al., 2014). When multiple tools are used in combination, they are referred to as test batteries. When choosing evaluation tools, clinicians should opt for standardized tools with normative data and good psychometric properties for evidence-based practice (Cooke et al., 2006).

Unrecognized VD can have a major impact on clients’ recovery process and prognosis. VD can go unrecognized or undiagnosed for several reasons. First, few rehabilitation settings in Canada focus on evaluation and treatment of VD following ABI (Leat, 2016; Robillard & Overbury, 2006; Trauzettel-Klosinski, 2010). Second, there is a lack of knowledge and communication between disciplines involved in ABI rehabilitation as to how various disciplines screen for VD (Leat, 2016; Roberts et al., 2016). Third, VD can be missed due to inadequate screening (Roberts et al., 2016; Trauzettel-Klosinski, 2010). Due to a frequent lack of vision specialists in the rehabilitation setting, VD screening often falls to the rehabilitation professionals who are already involved with the client (Erez et al., 2009; Jolly et al., 2013; Maxton et al., 2013). However, these professionals report lacking specialized training to assess VD (Copolillo et al., 2007; Jolly et al., 2013; Warren, 1995; Winner et al., 2014). Although Warren established a best-practice model of care for occupational therapists (OTs) that instructs clinicians to follow a bottom-up approach, a 2014 survey of American OTs found only between 54% and 63% of clinicians felt comfortable performing VD screening (Winner et al., 2014). Specifically, clinicians were unclear as to when they should evaluate clients for VD post-ABI, what assessment tools they should use and how to routinely use these tools in clinical practice.

Although several reviews have been conducted on interventions for clients with ABI with VD, few have examined assessments of this population in the rehabilitation setting. Of the 10 reviews conducted on evaluation tools for clients with stroke and TBI (Akhand et al., 2019a; Akhand et al., 2019b; Aksionoff & Falk, 1992; Hanna et al., 2017b; Hepworth et al., 2015; Hunt et al., 2016; Peterson, 2010; Pillai & Gittinger, 2017; Ventura et al., 2014; Ventura et al., 2015), none encompassed all conditions of adult ABI, most focused on one area of VD (e.g., unilateral spatial neglect [USN]). Eight reviews (Hanna et al., 2017a; Peterson, 2010) came from the fields of ophthalmology and neurology. Hepworth et al. (2015) and Pillai and Gittinger (2017) concluded that a combination of evaluation tools may be required for a thorough evaluation of VD in stroke and TBI. Hanna et al. (2017a) echoed these findings and concluded that multiple assessment tools should be used for evaluating USN, as there is no established gold standard. Akhand et al. (2019a) and Pillai and Gittinger (2017) also proposed several assessments for clients under 18 years who had VD and TBI.

A comprehensive literature review of assessment tools to evaluate VD in adults with ABI in the rehabilitation settings is lacking. Knowledge gained from this review may provide rehabilitation clinicians with up-to-date information and help develop adequate treatment plans (Ripley et al., 2010; Roberts et al., 2016). Our primary objective was to identify the evaluation tools used in rehabilitation settings to evaluate adult clients with ABI and VD, and report their psychometric properties. A secondary objective was to report on the timeframe when the tools should be administered after an ABI for best-practice recommendations (e.g., 1-day post-ABI vs. 1-month post-ABI).

### Ethics

As no novel human participant intervention was required, and secondary analyses were considered, this review is exempt from institutional ethics board approval.

## Methods

A scoping review methodology was used to explore and collate evidence in the literature relating to evaluation tools for ABI-related VD. Scoping reviews serve to examine the extent, range, and nature of research activity, to summarize and disseminate research findings as well as to identify gaps in the existing literature (Arksey & O’Malley, 2005; O’Brien et al., 2016; Peters et al., 2015). In this context, a scoping review methodology served to address a broad topic where several study designs were considered to answer a broad question without assessing the quality of studies.

This review is based on the Arksey and O’Malley (2005) framework and included the following phases: (1) identifying the research question, (2) identifying relevant studies, (3) study selection, (4) charting the data, (5) collating, summarizing, and reporting the results. A sixth optional consultation phase was originally planned (broad consultation of stakeholders) but was not carried out due to the COVID circumstances.

### Identifying the Research Question

The research question for this review was: *What is known about evaluation tools used in the rehabilitation setting to identify VD in adult clients with acquired brain injury?*

### Identifying Relevant Studies

Articles were retrieved on May 12, 2020, from the following databases since inception: MEDLINE(Ovid), Embase(Ovid), CINHAL, and PsycINFO(Ovid). The grey literature was searched using Google, Canadian Agency for Drugs and Technologies in Health (CADTH), OpenGrey, OTseeker, National Rehabilitation Information Center (NARIC), and Center Evidence-Based Physiotherapy (CEBP). Reference lists of relevant articles were also searched to ensure pertinent studies were not missed (Arksey & O’Malley, 2005).

Our search strategy was first developed in MEDLINE(Ovid) using MeSH terms (e.g., brain injuries, vision disorders) and keywords (e.g., hemineglect, evaluate) in collaboration with the first four authors, a team of clinicians, an expert methodologist, and a McGill librarian, and adapted for other databases. The MEDLINE(Ovid) search strategy is available in Supplemental Appendix A.

### Study Selection

The studies were included if they met the following criteria: (1) subjects had VD as a result of ABI; (2) subjects were above 14 years of age with at least 75% above 18 years old; (3) studies took place in a rehabilitation setting; (4) evaluation tools reported were used by healthcare professionals; (5) studies investigated the assessment of one or more VD; (6) studies reported psychometric properties of the evaluation tools; (7) study designs were cross-sectional, cohort, diagnostic accuracy, case-control, discriminant function analysis, randomized control trial, or repeated measures; and (8) articles published in English or French.

Studies were excluded based on the following criteria: (1) subjects had VD resulting from evolving conditions (e.g., degenerative neurological conditions, brain tumors); (2) tools which had a subscale on vision but did not primarily focus on vision (e.g., concussion assessments that focused on multiple concussion symptoms apart from VD); (3) neuroimaging assessment tools; and (4) study designs that were editorials, commentary, qualitative research, case report or series.

A pilot screening trial was first performed with four reviewers independently screening articles and comparing decisions until 90% consensus was reached, which was achieved after 64 articles. For remaining articles, pairs of reviewers independently screened the same set of articles for title and abstract based on the criteria, before reaching consensus. In case of disagreement, the other pair of reviewers was consulted to make the final decision. Potentially eligible articles underwent a full-text review, repeating the same process with pairs of reviewers independently screening and comparing decisions. The Preferred Reporting Items for Systematic Reviews and Meta-Analyses (PRISMA) flow diagram was used to report the study flow.

### Charting the Data

The data extraction process involved organizing the results into a logical and descriptive summary that aligns with the research question (Peters et al., 2015). We extracted pertinent information and characteristics from the selected studies, such as author, year of publication, purpose, population, methodology, outcome measure and key findings (Arksey & O’Malley, 2005).

The review team extracted information together for the first 5% of included studies to ensure consistency. Then, the team was divided into pairs, where each pair had two independent reviewers to source the results and keep careful records for each source.

### Collating, Summarizing, and Reporting the Results

The data aligning with the study objective was reported using descriptive numerical summary analysis using the data extraction chart. The number for each population type, study design, and evaluation tools was collated and summarized. In addition, a qualitative thematic analysis was used to identify, analyze and report common themes (e.g., areas of vision measured) (Braun & Clarke, 2006).

Furthermore, Warren’s (1993) hierarchical model of vision was used to map the assessment tools. Many evaluation tools did not fit into Warren's three-level model (foundation, intermediate, and high), as they assessed visual skills across more than one level. Therefore, an additional three classifications were created by the team (foundation & intermediate, intermediate & high, or all), for a total of six classifications levels.

Lastly, this review reported the psychometric properties of the tools, which includes sensitivity, specificity, predictive values, likelihood ratio, reliability, and validity with the last two reported as “excellent”, “adequate,” or “poor” based on Salter et al., (2005) and Andresen (2000). The sensitivity of a tool is defined as its ability to correctly identify people who have a condition, while the specificity is its ability to correctly identify those who do not have the condition (Lang & Secic, 1997). Predictive value is defined as the proportion of clients with a positive test who have the condition (e.g., positive predictive value) or those with a negative test who do not have the condition (e.g., negative predictive value) (Parikh et al., 2008). The likelihood ratio indicates the likelihood that a test result will be different in a client with the condition versus a client without the condition (Brown & Reeves, 2003). Reliability is the ability of a test to produce consistent results whereas validity is the degree to which a test measures what it claims to measure (Kline, 1986). When numerical values were not available, a qualitative description was provided where available. When sufficient numerical data was available, the reviewers calculated positive (*a*/(*a* + *b*)) and negative (*d*/(*c* + *d*)) predictive values, and positive (sensitivity/(1−specificity)) and negative ((1−sensitivity)/specificity) likelihood ratios. We did not assess the methodological quality of the included studies as the aim of this scoping review was to identify the breadth of the literature and the major areas of research activity with corresponding resulting themes.

## Findings

**Figure 1.**
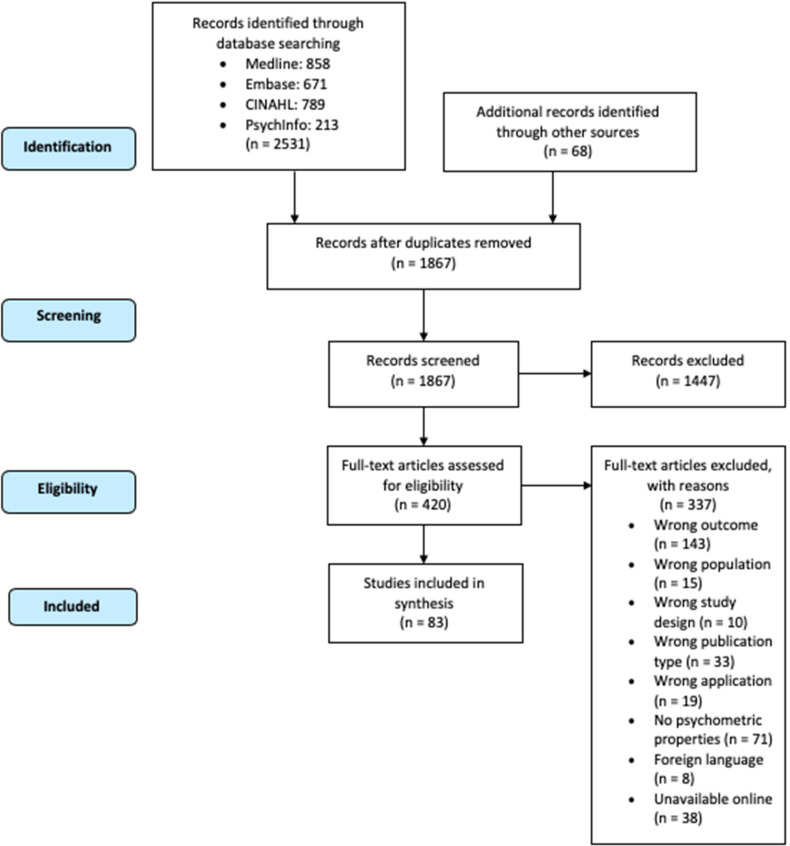
Preferred Reporting Items for Systematic Reviews and Meta-Analyses (PRISMA) flow diagram.

The search identified 2,531 articles across electronic databases and 68 hand-searched scholarly online resources, yielding 1,867 potentially eligible studies after duplicate removal. 1,447were excluded after screening titles and abstracts and 337 studies after full-text reviews. A total of 83 studies were included (Figure 1 PRISMA flow diagram).

Supplemental Appendix B reports the characteristics of the included studies. The studies were published between 1984 and 2020. Among the 83 studies, 63 (75.9%) were on stroke, 11 (13.2%) on TBI, and nine (10.8%) on mixed ABI conditions. Of the studies on stroke, 23 were published within the last 10 years and 40 were published between 1984 and 2009. All studies on TBI (except two) were published within the last 10 years. Of the studies on mixed ABI, four were published within the last 10 years and five were published between 1993 and 2008.

Most studies investigating psychometric properties were observational designs (cross-sectional [*n* = 38] or cohort [*n* = 14], case controls [*n* = 2]), reviews (*n* = 12), and diagnostic accuracy studies (*n* = 11). The three other studies were a discriminant function analysis, a randomized control trial, and a repeated measures design. Three sources came from grey literature (two books and the Stroke Engine website).

Nearly half (*n* = 39, 47%) of the studies reported using batteries or multiple evaluation tools to assess VD in their population. Supplemental Appendix C presents a summary of the areas of vision measured and Supplemental Appendix D presents a summary of the psychometric properties of the tools. Of the 86 tools, 60 focused on stroke, 12 on TBI, and 14 on both. There were 32 screening tools, 23 assessments, and 3 diagnostic tools. Eleven tools had multiple uses (e.g., used interchangeably as diagnostic, assessment, or screening) and eight were test batteries. Nine tools could not be classified.

The 86 tools were further divided by clinical disciplines. Almost half were used by OTs (*n* = 42, 48.8%), followed by psychologists and neuropsychologists (*n* = 16, 18.6%). Seven tools were used by vision specialists and four identified physiotherapists (PTs) as test users. 24 tools (27.9%) did not report which healthcare providers were the tool users.

The Behavioral Inattention Test (BIT) (*n* = 16), Line Bisection Test (LBT) (*n* = 12), Catherine Bergego Scale (CBS) (*n* = 11), and the Bells Test (*n* = 10) were the tools most frequently reported in the 83 included studies. As shown in Supplemental Appendix C, only five tools (5.8%) provided information on all psychometric properties. Sixteen tools (18.6%) had only one type of psychometric property reported. Five tools reported 100% sensitivity. These include the Ontario Society Occupational Therapists (OSOT) Perceptual Evaluation tool at the 110 cut-off (Boys et al., 1988) and the 70 and over cut-off (Fisher et al., 1991); the Tobii glasses eye-tracking monitoring task performance (Kortman & Nicholls, 2016); the VR-DiSTRO (Fordell et al., 2011); the Star Cancellation Test (SCT), Coin Sorting subtest, Line Cancellation Test (LCT), SCT, Figure Copying, Time Telling and Map Navigation subtests of the BIT (Figueiredo, 2011); and the Apple's Test (Bickerton et al., 2011).

**Figure 2.**
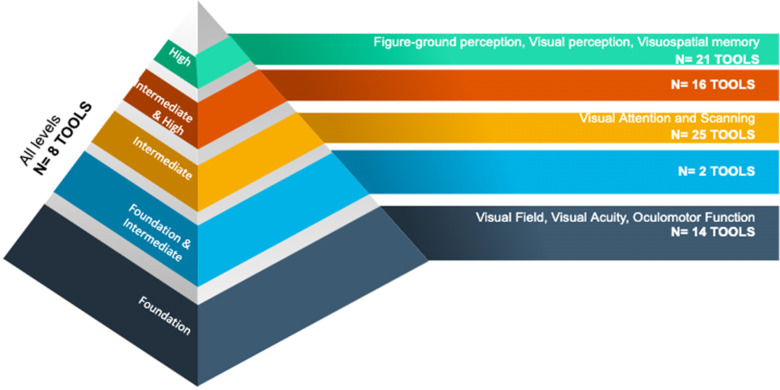
Tools corresponding to the hierarchy of visual perceptual skill development in the central nervous system adapted from Warren (1993).

Tools corresponding to each level of the hierarchy of visual perceptual skill adapted from Warren (1993) are described in Table 1 and represented in Figure 2. Most of the 86 tools evaluated intermediate skills (*n* = 25), intermediate and high-level skills (*n* = 16), and high-level skills (*n* = 21). Fewer tools assessed foundation skills and foundation and intermediate skills (*n* = 14 and *n* = 2, respectively). Eight tools evaluated various VD taken from all levels of the visual perceptual skill hierarchy.

**Table 1:** Tools Corresponding to the Hierarchy of Visual Perceptual Skill Development in the Central Nervous System Adapted From Warren (1993)

Level of visual perceptual skills	Name of the tool
All (*n* = 8)	Battery (Akinwuntan et al., 2006), Preliminary Assessment Battery of Anosognosia and Visual Extinction (Beis et al., 2004), BEN, BIVSS, MVPT/MVPT-3, SRWL, VSA, VSRT
High (*n* = 21)	Adult Visual-Perceptual Assessment, Ayres’ Figure-Ground Test, Ayres’ Space Visualization Test, Bender Visual Motor Gestalt Test, Block Design and Object Assembly subtest of the WAIS-R, CbVM, DTVP-A, Form H of Judgment of Line Orientation, Formboard Test, GEMAT, Haptic Visual Discrimination Test, HVOT, ImPACT, KVIQ-20/KVIQ-10, LOTCA/LOTCA-II/DLOTCA, Manikin and Feature Profile subtests of the Arthur Point Scale of Performance Tests, MIQ-RS, ROCF Test, Short assessment battery (Akinwuntan et al., 2007), Test of Three-Dimensional Constructional Praxis (3rd edition), TVPS
High and intermediate (*n* = 16)	Battery (Bailey et al., 2000), Battery (Barco et al., 2014), Battery (Saviola et al., 2018), CBS, CTT, Design Copy Test, Draw-A-Man Test, Light Show Device Tests, OSOT Perceptual Evaluation, OT-APST, RPAB, SAW, St. Marys CVA Evaluation Battery, TMT, UFOV, VRST
Intermediate (*n* = 25)	Albert Test, Apple's Test, Battery (Azouvi et al., 1996), Battery (Rorden et al., 2012), Bells test, BIT/BITC, BLO, BTT, CDT, DAT, DLCT, Greyscales task, HVST, Line bisection test, MAC, Scan Board Test, SCT, Semi-Structured Scale for the Functional Evaluation of Hemi-inattention Evaluation, SLCT, Sunnybrook Neglect Assessment Procedure, TOJ Test, Virtual Wheelchair Navigation Skills, VISSTA—Visual Spatial Search Task, VR-DiSTRO, VRLAT
Foundation and intermediate (*n* = 2)	Gross Visual Skills, Tobii glasses eye-tracking
Foundation (*n* = 14)	Checklist for Vision Problems Post Stroke, CHEERS, Developmental Eye Movement Test Eye Alignment Test, Eye movements recorded binocularly with video-oculography device, Eye-tracking assessments via an EyeLink 1,000 remote eye-tracking system, GST, King-Devick test, Read-Right, RightEye oculomotor tests, RightEye Vertical Smooth Pursuit test, Test of NPC, VOMS Tool, VV assessment

BEN, Batterie d’évaluation de la néglicence spatiale; BIVSS, Brain Injury Vision Symptom Survey; MVPT/MVPT-3, Motor-Free Visual Perception Test/Motor-Free Visual Perception Test-Third Edition; SRWL, Speeded Reading of Word Lists; VSA, Visual Scanning Analyzer; VSRT, Visual Skills for Reading Test; WAIS-R, Wechsler Adult Intelligence Scale-Revised; CbVM, Computer-based visuomotor task; DTVP-A, Developmental Test of Visual Perception—Adolescent and Adult; GEMAT, Gedachtnis Markaufsamkeit Test; HVOT, Hooper Visual Organization Test; ImPACT, Immediate Post-Concussion Assessment and Cognitive Test; KVIQ-20/KVIQ-10, Kinesthetic and Visual Imagery Questionnaire-20 items/Kinesthetic and Visual Imagery Questionnaire-10 items; LOTCA/LOTCA-II/DLOTCA, Loewenstein Occupational Therapy Cognitive Assessment/Loewenstein Occupational Therapy Cognitive Assessment-Second Edition/Dynamic Loewenstein Occupational Therapy Cognitive Assessment; MIQ-RS, Movement Imagery Questionnaire-Revised, Second Edition; ROCF Test, Rey–Osterrieth Complex Figure Test; TVPS, Test of Visual Perceptual Skills; CBS, Catherine Bergego Scale; CTT, Color Trails Test; OSOT, Perceptual Evaluation Ontario Society of Occupational Therapists Perceptual Evaluation; OT-APST, Occupational Therapy Adult Perceptual Screening Test; RPAB, Rivermead Perceptual Assessment Battery; SAW, Search-A-Word Test; TMT, Trail Making Tests; UFOV, Useful Field Of View; VRST, Visual Recognition Slide Test, BIT/BITC Behavioral Inattention Test/Conventional Behavioral Inattention Test, BLO Benton Judgement of Line Orientation; BTT, Baking Tray Test; CDT, Clock Drawing Test; DAT, Doorway accuracy test; DLCT, Double Letter Cancellation Test; HVST, Halifax Visual Scanning Test; MAC, Mobility Assessment Course; SCT, Star Cancellation Test; SLCT, Single Letter Cancellation Test; TOJ Test, Temporal Order Judgement Test; VRLAT, Virtual Reality Lateralized Attention Test; CHEERS, Craig Hospital Eye Evaluation Rating Scale; GST, Gaze Stabilization Test; Test of NPC, Test of Near Point Convergence; VOMS Tool, Vestibular/Ocular-Motor Screening Tool; VV assessment, Visual Vertical assessment.

**Figure 3.**
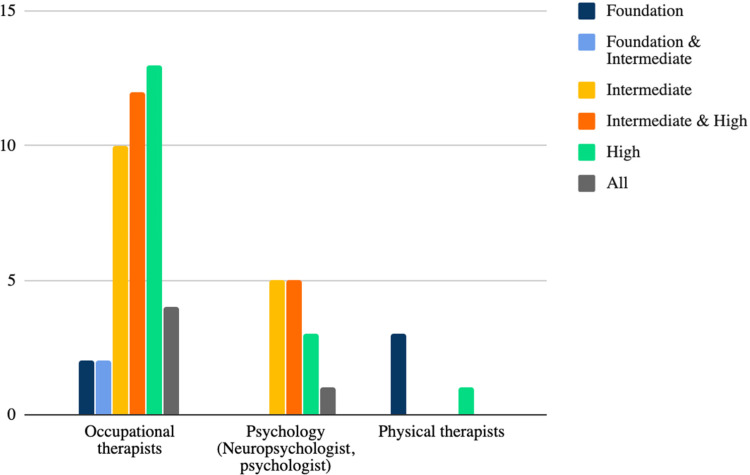
Number of tools by health care disciplines in the rehabilitation setting and level of vision assessed.

OTs and psychologists were found to primarily use tools that evaluate intermediate to high-level skills, whereas vision specialists and PTs evaluated primarily foundation skills (Figure 3).

The second study objective was to determine when the evaluation tools should be administered. No studies made any recommendations as to an ideal timeframe for assessment/screening.

## Discussion

This scoping review explored what is known about evaluation tools used in the rehabilitation setting to identify VD in adult clients with ABI. More evaluation tools assessed VD associated with stroke than any other ABI condition. Most studies which assessed complex VD or focused on comprehensive visual assessment used multiple tests. Results suggest that healthcare professionals primarily select assessment tools that are associated with the level of the hierarchy of visual perceptual skills most easily aligned with their scope of practice. OTs and psychologists mainly assess VD in the intermediate to high-level visual skills, which are commonly associated with difficulties in performing ADLs and higher cognitive activities. Contrarily, vision specialists and PTs used tools evaluating VD in foundation skills, which are typically associated with difficulties with fundamental movement skills such as head righting and gait.

This review differed from previous reviews in several ways. First, it examined and compared assessment of VD across multiple ABI conditions rather than only one condition. This highlighted important trends and gaps in research. For example, the focus of most TBI research is on a young cohort and most reviews focus on discrete areas of VD rather than a wide spectrum of VD assessment (Akhand et al., 2019a; Cavanagh, 2011; Pillai & Gittinger, 2017). Second, by extracting data on the use and development of assessment tools across several healthcare disciplines, we identified possible knowledge-practice gaps in the respective disciplines. This includes the possible lack of assessment of low-level VD by OTs and of high-level VD by PTs.

Our findings align with three other reviews (Akhand et al., 2019a; Ventura et al., 2014; Ventura et al., 2015) regarding the King-Devick test as a valid tool for assessment of saccadic eye movement following TBI. The Hanna et al. (2017a) review on the assessment of USN post-stroke found no gold standard for assessing USN, and concluded that clinicians should preferably use multiple tools to assess USN; our findings validate this conclusion.

### Relevance for Rehabilitation Clinicians

Almost half of the included studies presented batteries or the use of multiple evaluation tools to assess VD. While individual tools can provide valuable information on a specific VD, these are often limited to one or a few visual components. In contrast, a whole test battery is shown to be more sensitive than the individual tools (e.g., the BIT) (Azouvi et al., 2006; Maxton et al., 2013). Therefore, using a wide range of evaluation tools allows for the development of a better and more comprehensive rehabilitation plan (Titus et al., 1991). This is the case for USN; although paper-and-pencil tools have shown good psychometric properties (Azouvi et al., 2006), they fail to demonstrate the reality of the everyday occupational performance of the client. The CBS (a measure of behavioral neglect) paired with the BIT (which measures visual inattention) can capture different symptoms of USN, each having distinct repercussions on the client's everyday life (Azouvi et al., 2006; Luukkainen-Markkula et al., 2011; Maxton et al., 2013).

Only 14 tools were researched on both stroke and TBI populations. These demonstrated adequate to excellent psychometric properties for both conditions and did not show notable differences in the application of the tools. Nonetheless, the other 72 tools were researched on only one of the conditions. With little overlap in the evaluation tools, this review was unable to draw firm conclusions regarding differences between the assessment of VD in stroke versus TBI.

Most of the assessment tools identified in our review were used by OTs. Indeed, OTs tend to see clients soon after an acute neurological event, and spend a significant amount of time with them, thus enabling the observation of higher-order cerebral mechanisms of vision (Roberts et al., 2016; Robillard & Overbury, 2006). However, most of the identified OT tools assessed the high levels of vision. This raises concerns as to the accurate assessment of client VD. Warren (1990) warns that a top-down, non-comprehensive VD assessment can lead clinicians to misidentify lower-level VD. For example, a deficit in visual scanning can be misidentified as a figure-ground perception deficit if a client is given a visual-perceptual test without prior screening for lower-level skills (Warren, 1990). This is of particular concern as clients with ABI will usually be referred to vision specialists only if it is suspected by the acute/rehabilitation team that VD are present (Jolly et al., 2013; Rowe, 2010). Similarly, OTs may not have expertise regarding other low-level VD that impact areas of concern such as risk of falls, balance, and adequate postural modulation which are familiar to PTs (Jilk et al., 2014; Reed-Jones et al., 2013; Tomomitsu et al., 2013). Thus, this would suggest that OTs working with clients with ABI and VD should ensure they seek out training themselves in the assessment of low-level VD, or alternately, partnering with another professional trained in low-level VD assessment.

Our second study objective was to determine when the evaluation tools should be administered to clients post-ABI. As none of the included studies reported an ideal timeframe, no recommendation could be made. Furthermore, since the prognosis for TBI and stroke are different, the testing timeframe would most probably differ as well (Tippett et al., 2013). Variability exists between individuals as the evolution of the condition can also differ.

Previous research found that clients with ABI in the rehabilitation setting generally do not receive a formal vision assessment by vision specialists who are experts in diagnosing foundational VD (Groffman, 2011; Jolly et al., 2013; Rowe, 2010; Warren, 1993). Thus, if there is an absence of vision specialists, it is important for the rehabilitation team to ensure a comprehensive evaluation of vision for clients with ABI, including an early evaluation of foundation skills. Adopting a multidisciplinary approach, increasing the involvement of vision specialists, and clearly communicating assessment results between all team members will aid in best-practice, in line with recommendations for the development of clinical treatment models and guidelines (Leat, 2016; Roberts et al., 2016; Robillard & Overbury, 2006). Lastly, specialized training offered to rehabilitation professionals in vision evaluation could improve clinicians’ ability to understand and recognize VD (Jolly et al., 2013).

### Areas for Further Research

Given that our study primarily focused on adults (75% above 18 years old), and that TBI injury is most prevalent in males aged 15–24 years old (Pickett et al., 2001), future reviews targeting VD in a younger TBI cohort in the rehabilitation setting are warranted. Although most studies were published within the last 10 years, many of the evaluation tools assessing VD in adults with ABI have poorly established psychometric properties. As previous research documented that brain damage causes increased variability in performance among the chronic stroke group (Tippett et al., 2013), evaluation tools with good psychometric properties that are also able to capture variability in performance are needed. As none of the studies reported an ideal testing timeframe for assessing vision post-ABI, future research should also address when evaluation tools should be used to ensure best practice.

### Strengths and Limitations

Warren's hierarchical model used to collate the data is familiar to many rehabilitation professionals working in low vision rehabilitation both as a theoretical and treatment/assessment model. This allows for the results to be easily interpreted by clinicians and serve as a quick clinical reference.

Nonetheless, this review has some limitations. First, defining the boundaries of VD is challenging as there exist different definitions for the same construct by different authors. Although in clinical settings “low-vision rehabilitation” refers to a discreet area of rehabilitation, this does not accurately represent the complex neurological processes involved in vision. We applied our inclusion/exclusion criteria based on the author's report that the tools were primarily used to measure a defined VD (e.g., the OSOT, BIT, etc.). Second, since the inclusion criteria of this review included solely VD, combined vestibular/visual measures found in recent concussion literature were excluded. Third, few of the included studies were conducted in ophthalmology. This may be due to our search strategy omitting keywords and MeSH terms specific to vision tests related to the physiopathology of vision disorders and visual pathways often studied by vision specialists. Studies from the field of ophthalmology could provide further insight on the continuum of care for the visual evaluation for clients with ABI within multidisciplinary care. Fourth, much of the current TBI research is conducted on young athletes and military members. Our review may have excluded useful TBI literature due to the age limitation. Last, this review did not formally evaluate the quality of evidence gathered from the wide range of study designs and methods. Thus, the reported psychometric properties of the tools should be interpreted with caution.

## Conclusion

This scoping review has highlighted the complexity in evaluating VD following ABI in the rehabilitation setting. Many evaluation tools exist and are often used by OTs to evaluate visual skills. To ensure a comprehensive evaluation of vision, OTs need to use multiple tools and batteries and involve multiple professionals (e.g., vision specialists, PTs, psychologists) in the rehabilitation process for a holistic approach. Further research is needed to address the optimal evaluation timeframe for both stroke and TBI, to better understand the role of each professional on the rehabilitation team, and to investigate the quality of tools published more recently.

## Key Messages

- When selecting an assessment tool, clinicians should consider what VD it evaluates and if it is appropriate for the target condition (stroke vs. TBI).
- Findings from this review suggest OTs contribute to multidisciplinary and comprehensive evaluation of VD in adults with ABI by primarily evaluating the intermediate to higher-level skills of visual perception.
- The psychometric properties of many commonly used assessment tools for the ABI population are currently incompletely researched.

## Supplemental Material

sj-docx-1-cjo-10.1177_00084174211042955 - Supplemental material for Vision Evaluation Tools for Adults With Acquired Brain Injury: A Scoping Review Click here for additional data file. Supplemental material, sj-docx-1-cjo-10.1177_00084174211042955 for Vision Evaluation Tools for Adults With Acquired Brain Injury: A Scoping Review by Camille Dubé, Yu Jin, Brienne G. Powers, Ginny Li, Amélie Labelle, Meghan S. Rivers, Ivy M. Gumboc and André E. Bussières in Canadian Journal of Occupational Therapy

sj-docx-2-cjo-10.1177_00084174211042955 - Supplemental material for Vision Evaluation Tools for Adults With Acquired Brain Injury: A Scoping Review Click here for additional data file. Supplemental material, sj-docx-2-cjo-10.1177_00084174211042955 for Vision Evaluation Tools for Adults With Acquired Brain Injury: A Scoping Review by Camille Dubé, Yu Jin, Brienne G. Powers, Ginny Li, Amélie Labelle, Meghan S. Rivers, Ivy M. Gumboc and André E. Bussières in Canadian Journal of Occupational Therapy

sj-docx-3-cjo-10.1177_00084174211042955 - Supplemental material for Vision Evaluation Tools for Adults With Acquired Brain Injury: A Scoping Review Click here for additional data file. Supplemental material, sj-docx-3-cjo-10.1177_00084174211042955 for Vision Evaluation Tools for Adults With Acquired Brain Injury: A Scoping Review by Camille Dubé, Yu Jin, Brienne G. Powers, Ginny Li, Amélie Labelle, Meghan S. Rivers, Ivy M. Gumboc and André E. Bussières in Canadian Journal of Occupational Therapy

sj-docx-4-cjo-10.1177_00084174211042955 - Supplemental material for Vision Evaluation Tools for Adults With Acquired Brain Injury: A Scoping Review Click here for additional data file. Supplemental material, sj-docx-4-cjo-10.1177_00084174211042955 for Vision Evaluation Tools for Adults With Acquired Brain Injury: A Scoping Review by Camille Dubé, Yu Jin, Brienne G. Powers, Ginny Li, Amélie Labelle, Meghan S. Rivers, Ivy M. Gumboc and André E. Bussières in Canadian Journal of Occupational Therapy
