# Supplementary material for: Vision Evaluation Tools for Adults With Acquired Brain Injury: A Scoping Review
Source: Can J Occup Ther. 2021 Oct 18;88(4):340–51. doi: 10.1177/00084174211042955 (PMC8640270; doi:10.1177/00084174211042955)
Supplement: sj-docx-1-cjo-10.1177_00084174211042955 - Supplemental material for Vision Evaluation Tools for Adults With Acquired Brain Injury: A Scoping Review [file sj-docx-1-cjo-10.1177_00084174211042955.docx]

Appendix A

MEDLINE Search Strategy

Database: Ovid MEDLINE(R) ALL <1946 to May 12, 2020>

Search Strategy:

--------------------------------------------------------------------------------

1 Brain Concussion/ or Post-Concussion Syndrome/ (8748)

2 exp Brain Injuries/ (68287)

3 exp Stroke/ (132629)

4 cerebrovascular disorders/ or intracranial hemorrhages/ or cerebral hemorrhage/ (83205)

5 exp Craniocerebral Trauma/ (157201)

6 hematoma/ or hematoma, epidural, cranial/ or hematoma, subdural/ (31973)

7 exp Intracranial Aneurysm/ (27956)

8 cerebral infarction/ or infarction, anterior cerebral artery/ or infarction, middle cerebral artery/ or infarction, posterior cerebral artery/ (30316)

9 Cerebrovascular Trauma/ (184)

10 (brain injur* or acquired brain injur* or lesion or diffuse brain injur* or hematoma or cerebral vision impairment or cerebrovascular accident or close head injur* or cortical contusion or hematoma or cerebral hemorrhage or concussion or cerebral infarct or traumatic brain injur* or penetrating cranial injur* or post-stroke or stroke or vascular brain injur* or open head injur* or diffuse axonal injur* or post-concussion syndrome).tw,kf. (663074)

11 1 or 2 or 3 or 4 or 5 or 6 or 7 or 8 or 9 or 10 (870191)

12 exp Vision Disorders/ (71478)

13 exp Vision, Ocular/ (27570)

14 blindness/ or amaurosis fugax/ or blindness, cortical/ (20626)

15 exp Visual Acuity/ (81189)

16 Hemianopsia/ (2777)

17 Form Perception/ (15545)

18 exp Ocular Motility Disorders/ (40422)

19 Fixation, Ocular/ (13269)

20 Diplopia/ (5222)

21 Photophobia/ (749)

22 Ophthalmoplegia/ (7989)

23 Visual Perception/ (61970)

24 Nystagmus, Pathologic/ (8318)

25 Visual Fields/ (30182)

26 Accommodation, Ocular/ (6315)

27 exp Agnosia/ (3064)

28 Saccades/ (10324)

29 exp Depth Perception/ (11221)

30 exp Strabismus/ (15864)

31 exp Space Perception/ (59993)

32 (accommodation insufficienc* or achromatopsia or blindness or visual or vision or binocular function or bitemporal hemianopia or gaze or colour discrimination or colour discrimination or acuity or constancy of form or convergence insufficienc* or diplopia or eye function* or eye movement* or figured ground discrimination or fixation instability or hemi-neglect or hemianopia or abducens nerve or hemianopsia or light sensitivity or nystagmus or neuro-optometric* or ocular function* or ocular motor palsy or oculo-motor or optic chiasm or optic nerve* or primary visual cortex or prosopagnosia or saccade insufficienc* or simultagnosia or spatial contrast sensitivity or stereoacuity or strabismus or vestibular-ocular reflex or accommodative dysfunction or stereopsis or ocular alignment* or eye strain* or depth perception* or pursuit* or oculomotor nerve or trochlear nerve or hemispatial neglect or visuomotor or midline shift*).tw,kf. (610231)

33 12 or 13 or 14 or 15 or 16 or 17 or 18 or 19 or 20 or 21 or 22 or 23 or 24 or 25 or 26 or 27 or 28 or 29 or 30 or 31 or 32 (728538)

34 exp Neuropsychological tests/ (175842)

35 exp surveys/ and questionnaires/ (457999)

36 Psychometrics/ (74550)

37 ((screen* or evaluat* or assess* or diagnos* or rating or rate or measur* or case finding) adj3 (index or test* or instrument* or inventor* or battery or batteries or tool* or scale* or checklist* or check list* or schedule*)).tw,kf. (631181)

38 34 or 35 or 36 or 37 (1191137)

39 11 and 33 and 38 (5064)

40 exp Rehabilitation/ (301683)

41 neurological rehabilitation.mp. or exp Neurological Rehabilitation/ or exp Stroke Rehabilitation/ (14416)

42 Occupational Therapy/ or Occupational Therapists/ (13267)

43 Physical Therapists/ or Physical Therapy Modalities/ (37729)

44 (rehab* or physiotherap* or physical therap* or occupational therap* or vision rehab* or visual therapy or vision therapy).tw,kf. (222141)

45 40 or 41 or 42 or 43 or 44 (446956)

46 39 and 45 (858)
