# Supplementary material for: Vision Evaluation Tools for Adults With Acquired Brain Injury: A Scoping Review
Source: Can J Occup Ther. 2021 Oct 18;88(4):340–51. doi: 10.1177/00084174211042955 (PMC8640270; doi:10.1177/00084174211042955)
Supplement: sj-docx-2-cjo-10.1177_00084174211042955 - Supplemental material for Vision Evaluation Tools for Adults With Acquired Brain Injury: A Scoping Review [file sj-docx-2-cjo-10.1177_00084174211042955.docx]

Appendix B

Study characteristics

| **Author(s), year** | **Purpose of the study** | **Population, sample size (n)** | **Study design** | **Name(s) of the evaluation tool (s)** |
| --- | --- | --- | --- | --- |
| Akinwuntan et al. (2002) | To identify variables that best predict a team's decision of driving ability in stroke patients from a pre-driving assessment. | Stroke (n = 104) | Cohort study (retrospective) | (1) Monocular Vision Acuity test; (2) Binocular Vision Acuity test; (3) Stereoscopy; (4) Kinetic Vision test;(5) Figure of Rey; (6) Useful Field of View (UFOV); (7) Divided Attention test (visual part); (8) Visual Scanning test; (9) Visual Field and Neglect test |
| Akinwuntan et al. (2006) | To identify the combination of tests from a pre-driving assessment that best predicted the outcome of a group decision of stroke patients’ driving fitness in Belgium. | Stroke (n = 68) | Cohort study (prospective) | (1) Monocular Visual Acuity test; (2) Binocular Visual Acuity test; (3) Kinetic Vision test; (4) Figure of Rey; (5) Useful Field of View (UFOV); (6) 5 out of the 6 tests in the Test for Attentional Performance (TAP) battery; (7) Stroke Driver Screening Assessment (SDSA); (8) Checklist adapted from the Test Ride for Investigating Practical fitness to drive (TRIP) |
| Akinwuntan et al. (2007) | To confirm the accuracy of a short assessment battery, used previously in a study to predict fitness-to-drive after stroke, in a new cohort of stroke survivors without severe deficits. | Stroke (n = 43) | Cohort study (prospective) | (1) Figure of Rey; (2) Visual Neglect test, a part of the Test for Attentional Performance (TAP) battery; (3) On-road test |
| Amesz, Tessari, Ottoboni, and Marsden (2016) | To explore the relationship between laterality recognition after stroke and impairments in attention, 3D object rotation and functional ability. | Stroke (n = 32) | Cross-sectional study | (1) Hand Laterality task via Recognise TM; (2) Two pictures of Shepard-Metzler objects; (3) Catherine Bergego Scale (CBS); (4) Computerized Connor’s Continuous Performance Test II (CCPT) (Version 5) |
| Appelros, Nydevik, Karlsson, Thorwalls, and Seiger (2004) | To show the recovery process for different forms of unilateral neglect (UN)—including personal neglect and neglect of far space—in relationship to impairment, disability, cognition and mood. | Stroke (n = 37) | Cohort study (prospective) | (1) Behavioral Inattention Test (BIT) |
| Azouvi et al. (1996) | To evaluate the validity and sensitivity of the Catherine Bergego Scale (CBS), designed to provide therapists with an ecological assessment of explorative disorders related to neglect in patients' everyday life. | Stroke (n = 50) | Diagnostic accuracy study | (1) Copy-a-Daisy; (2) Ogden’s scene; (3) Line Cancellation; (4) Bells test; (4) Reading task |
| Azouvi et al. (2002) | To assess the sensitivity of different tests of neglect after right hemisphere stroke. Another aim of this study was to relate performance on conventional tests to behavioural neglect. | Stroke: study 1 (n = 206); study 2 (n = 69) | Cross-sectional study | (1) Visual Extinction and Hemianopia; (2) Gaze and Head Orientation; (3) Personal Neglect; (4) Bells test; (5) Figure Copying; (6) Clock Drawing; (7) Line Bisection Test; (8) Overlapping Figures test; (9) Reading; (10) Writing; (11) Catherine Bergego Scale (CBS) |
| Azouvi et al. (2006) | To present the validation studies of a quantitative test battery for USN, including paper-and-pencil tests, an assessment of personal neglect, extinction, and anosognosia, and a behavioural assessment, the Catherine Bergego Scale (CBS). | Stroke: study 1 (n = 206); study 2 (n = 54) | Cross-sectional study | (1) Behavioural Inattention Test (BIT); (2) Batterie d'Évaluation de la Négligence Spatiale (BEN); (3) Catherine Bergego Scale (CBS) |
| Bailey, Riddoch, and Crome (2000) | To measure performance of healthy elderly subjects on a battery of tests for hemineglect in order to establish appropriate cut-off scores, to assess performance of a large sample of elderly acute stroke patients using the same battery, and to evaluate the clinical usefulness of the test battery in relation to test sensitivity, and simplicity of application in the clinical situation. | Stroke (n = 107) | Case control study | (1) Star Cancellation Test (SCT); (2) Line Bisection Test; (3) Copy-a-Daisy; (4) The Baking Tray Task (BTT); (5) Draw-a-Clock; (6) Exploratory Motor (EM) Task; (7) Personal Neglect (PN) test |
| Barco, Wallendorf, Snellgrove, Ott, and Carr (2014) | To develop a predictive model of driving performance for people with stroke by using a combination of brief cognitive screens that could be easily administered by clinicians and adopted at low cost in a variety of settings. | Stroke (n = 72) | Cross-sectional study | (1) far and near visual acuity and fields with the Optec 5500P; (2) Contrast sensitivity was tested using the Pelli–Robson contrast sensitivity chart; (3) Clock Drawing test; (4) Snellgrove Maze Test; (5) Trail Making Test Part A; (6) Trail Making Test Part B; (7) Subtest 2 of the UFOV; (8) Motor-Free Visual Perceptual Test (MVPT) |
| Barker-Collo, Feigin, Lawes, Senior, and Parag (2010) | To identify the frequency of various forms of attention deficit (both visual and auditory) after first-ever stroke and to examine relationships between measures of attention and measures of disability, handicap, and health-related quality of life. | Stroke (n = 94) | Cross-sectional study | (1) Bells Test; (2) The Integrated Visual Auditory Continuous Performance Test (IVA-CPT); (3) Trail Making Tests A and B |
| Basagni et al. (2017) | To compare the diagnostic accuracy of two widespread tests often used to assess USN, the Apples and Bells tests, in the assessment of egocentric neglect, in patients with right hemisphere stroke. | Stroke (n = 56) | Case control study (retrospective) | (1) Bells test |
| Blaylock, Warren, Yuen, and DeCarlo (2016) | To preliminarily validate the Visual Skills for Reading Test (VSRT) for assessing reading performance in persons with homonymous hemianopia (HH) or quadrantanopia. | ABI (n = 38) | Review (retrospective chart) | (1) Visual Skills for Reading Test (VSRT) or the Pepper Test |
| Beis et al. (2004) | To assess contralateral neglect in subacute left hemisphere stroke patients using a comprehensive test battery validated in a large control group after right hemisphere stroke. | Stroke (n = 78) | Cross-sectional study | (1) Gaze and Head Orientation; (2) Personal Neglect; (3) Bells test; (4) Figure Copying; (5) Clock Drawing; (6) Line Bisection Test; (7) Awareness; (8) Visual Extinction and Hemianopia |
| Bickerton, Samson, Williamson, and Humphreys (2011) | To report data on the validation and functional correlates of Apples Test, which attempts to differentiate between different forms of unilateral neglect. | Study 1: Acquired brain lesions (n = 25), left hemisphere lesion (n = 7), right hemisphere lesion (n = 18). Study 2 & 3: Acute stroke (n = 115) | Cross-sectional study | Study 1: (1) The Apples test; Study 2: (2) Birmingham University Cognitive Screen (BUCS) [Apples subtest] |
| Bohannon (2003) | To summarize research literature addressing the evaluation and treatment of these impairments. | Stroke | Review (literature) | (1) Rivermead Perceptual Assessment Battery (RPAB); (2) BIT; (3) Drawing and Copying tests; (4) Line Bisection Test; (5) Cancellation tests |
| Brown, Mapleston, and Nairn (2011) | To investigate the convergent validity of the Occupational Therapy Adult Perceptual Screening Test (OT-APST) with two other cognitive-perceptual tests: the Neurobehavioural Cognitive Status Examination (Cognistat) and the Developmental Test of Visual Perception – Adolescent and Adult (DTVP-A). | Stroke (n = 32) | Cross-sectional study | (1) Occupational Therapy Adult Perceptual Screening Test (OT-APST) |
| Brown, Mapleston, and Nairn (2012) | To assess three standardized cognitive and visual perceptual assessments for their ability to predict functional performance in adults who have had a stroke. | Stroke (n = 27) | Cross-sectional study (pilot) | (1) Neurobehavioural Cognitive Status Examination (Cognistat); (2) Developmental Test of Visual Perception – Adolescent and Adult (DTVP-A); (3) Occupational Therapy Adult Perceptual Screening Test (OT-APST) |
| Butler et al. (2012) | To examine and compare the test-retest intra-rater reliability of the Movement Imagery Questionnaire- Revised, second Edition (MIQ-RS) in stroke survivors and able-bodied controls, examine internal consistency of the visual and kinesthetic items of the MIQ-RS, determine if the MIQ-RS includes both the visual and kinesthetic dimensions of mental imagery, correlate impairment and motor imagery scores, and investigate the criterion validity of the MIQ-RS in stroke survivors by comparing the results to the KVIQ-10. | Stroke (n = 23) | Diagnostic accuracy study (reliability and validity) | (1) Movement Imagery Questionnaire-Revised, Second Edition (MIQ-RS) |
| Calvanio et al. (2004) | To compare the findings of the Useful Field of View (UFOV) test with those of conventional neuropsychological tests to determine the utility of the UFOV test as a measure of attention in a population with brain injury. | Severe brain injury (n = 15) | Cohort study (pilot) | (1) Useful Field of View (UFOV) |
| Cassidy, Lewis, and Gray (1998) | To describe the natural recovery of visuospatial neglect in stroke patients and the distribution of errors made on cancellation tests using a standardised neuropsychological test battery. | Stroke (n = 66) | Cohort study (prospective) | (1) Behavioural Inattention Test (BIT) |
| Cate and Richards (2000) | To investigate the relationship between basic visual functions (including acuity, visual field deficits, oculomotor skills, and visual attention or scanning) and higher level visual perceptual processing skills (e.g., visual closure and figure–ground discrimination). | Stroke (n = 30) | Diagnostic accuracy study (correlational validity) | (1) Motor-Free Visual Perception Test (MVPT) |
| Cermak and Hausser (1989) | To establish what is known about the Behavioural Inattention Test. | Stroke (n = 80) | Review (critical) | (1) Behavioural Inattention Test (BIT) |
| Chiu, Wu, Chou, Yu, and Hung (2016) | To examine the test-retest reliability, calculate minimal detectable change (MDC), and report internal consistency of the Test of Visual Perceptual Skills-Third Edition (TVPS-3) in patients with stroke. | Stroke (n = 50) | Repeated-measures study | (1) Test of Visual Perceptual Skills-Third Edition (TVPS-3) |
| Chiu et al. (2019) | To investigate ecological validity, convergent validity, and discriminative validity of the TVPS-3 in patients with stroke. | Stroke (n = 100) | Cross-sectional study | (1) Test of Visual Perceptual Skills - Third Edition (TVPS-3) |
| Cooke, McKenna, and Fleming (2005) | To describe the variations in visual perception terminology and occupational therapy approaches to visual perceptual assessment. To describe the Occupational Therapy Adult Perceptual Screening Test (OT-APST). | ABI | Review (opinions) | (1) Occupational Therapy Adult Perceptual Screening Test (OT-APST) |
| Cooke, McKenna, Fleming, and Darnell (2005) | To report three aspects of the reliability of the Occupational Therapy Adult Perceptual Screening Test (OT-APST): interrater, intrarater and test-retest reliability. | Stroke (n = 15) | Diagnostic accuracy study (reliability) | (1) Occupational Therapy Adult Perceptual Screening Test (OT- APST) |
| Cooke, McKenna, Fleming, and Darnell (2006a) | To describe the variations in visual perception terminology and OT approaches to visual perceptual assessment. | Stroke (n = 208) | Review (opinions) | (1) Occupational Therapy Adult Perceptual Screening Test (OT- APST) |
| Cooke, McKenna, Fleming, and Darnell (2006b) | To report the criterion validity of the Occupational Therapy Adult Perceptual Screening Test (OT-APST) including examination of test sensitivity and specificity at selected cut-off points. | Stroke (n = 208) | Diagnostic accuracy study (validity) | (1) Occupational Therapy Adult Perceptual Screening Test (OT-APST) |
| Donnelly (2002) | To investigate if a relationship between the Rivermead Perceptual Assessment Battery (RPAB) and functional performance, as measured by the Functional Independence Measure (FIM), did exist and second, to investigate whether the RPAB admission scores could predict the functional performance of a person at discharge from rehabilitation following a stroke. | Stroke (n = 46) | Cross-sectional study | (1) Rivermead Perceptual Assessment Battery (RPAB) |
| Dunlap et al. (2020) | To examine the gaze stabilization test (GST) in those referred for vestibular physical therapy following concussion, to determine the association between the GST and other measures of recovery following concussion, and to examine the effect of demographic variables on GST performance. | Concussion (n = 158) | Review (retrospective chart) | (1) Gaze Stabilization Test (GST) |
| Erez, Katz, Ring, and Soroker (2009) | To assess the diagnostic sensitivity of tasks employing feature and conjunction visual searches in stroke patients with unilateral spatial neglect (USN). | Stroke (n = 72) | Cross-sectional study | (1) Computerised visual search test and training program (VISSTA – Visual Spatial Search Task) |
| George, Clark, and Crotty (2008) | To determine the construct and predictive validity of the New South Wales Visual Recognition Slide Test (VRST) — a component of the occupational therapy off-road driving rehabilitation program designed to assess the abilities of speed of information processing and visual scanning by determining how well it is related to an assessment of scanning (Visual Scanning Analyser (VSA)) and an assessment of speed of information processing (Response Time Measures (RTM)). | Stroke, Construct validity study (n = 26); Predictive validity study (n = 24) | Diagnostic accuracy study (validity) | (1) Visual Recognition Slide Test (VRST) |
| Greve, Lindberg, Bianchini, and Adams (2000) | To contribute to the ongoing discussion by further examining the construct validity of the Hooper Visual Organization Test (HVOT) in a sample of stroke patients referred for neuropsychological evaluation while being treated in a comprehensive inpatient physical rehabilitation program. | Stroke (n = 98) | Diagnostic accuracy study (validity) | (1) Hooper Visual Organization Test (HVOT) |
| Halligan, Wilson, and Cockburn (1990) | To extend a preliminary study of the internal structure of six measures compromising the "conventional' subtests of the Behavioural Inattention Test (BIT) in order to develop a short screening test for visual neglect. | Stroke (n = 59) | Discriminant function analysis | (1) BIT |
| Harlowe and Van Deusen (1984) | This study was conducted to continue the construct validation of the occupational therapy Cerebral Vascular Accident (CVA) evaluation used at St. Marys Hospital Medical Center in Madison, Wisconsin. | Stroke (n = 53) | Cross-sectional study | (1) St. Marys CVA Evaluation Battery: Perceptual Measures |
| Hartman-Maeir, Erez, Ratzon, Mattatia, and Weiss (2008) | To examine the predictive and convergent validity of the Colour Trails Test (CTT) as a screening test in the pre-driver evaluation process for individuals with ABI. | ABI (n = 30) | Cohort study (retrospective) | (1) Colour Trails Test (CTT); (2) Useful Field of View (UFOV) |
| Hunfalvay et al. (2019) | To measure horizontal and vertical saccades with eye tracking tests as a proxy for neural deficits associated with TBI. | TBI (n = 195) | Cross-sectional study | (1) RightEye oculomotor tests (Horizontal Saccades and Vertical Saccades test) |
| Hunfalvay et al. (2020) | To evaluate the ability of the eye tracking tests to differentiate between different levels of TBI severity and healthy controls. | TBI (n = 69) | Cross-sectional study | (1) RightEye Vertical Smooth Pursuit test (RightEye, LLC, MD, USA) |
| Jolly, Macfarlane, and Heard (2013) | To report on the development and evaluation of a tool, to be used by any healthcare practitioner, to screen for the presence of eye issues and problems in patients who have been diagnosed to have had a stroke. | Stroke (n = 100) | Cohort study (retrospective) | (1) Checklist for Vision Problems Post Stroke |
| Kettunen, Nurmi, Dastidar, and Jehkonen (2012) | To examine the presence of rightward bias after right hemisphere stroke within 10 days of stroke onset and after 6 months. | Stroke (n = 43) | Cross-sectional study | (1) Conventional subtests of the Behavioural Inattention Test (BIT C) |
| Koiava et al. (2012) | To validate the Read-Right test by comparing with a clinical ‘gold standard’ - the Humphrey automated visual field analyser. | ABI (n = 23) | Cross-sectional study | (1) Read-Right |
| Kontos, Deitrick, Collins, and Mucha (2017) | To evaluate current findings on vestibular and oculomotor impairments as well as treatment approaches after sport-related concussion and to highlight areas in which investigation is needed. | TBI (concussion) | Review | N/A |
| Korner-Bitensky et al. (2000) | To determine the ability of a visual-perception assessment tool, the Motor-Free Visual Perception Test, to predict on-road driving outcome in subjects with stroke. | Stroke (n = 269) | Cohort study (retrospective) | (1) Motor-Free Visual Perception Test (MVPT) |
| Kortman and Nicholls (2016) | To establish proof of concept as to whether eye-tracking is a successful method of differentiating between people with USN and those without following stroke in the instrumental activity of daily living (IADL) task of making a cup of coffee. The secondary objective was to investigate how effective the eye-tracking glasses are in detecting USN compared to two common paper-based assessments. | Stroke (n = 11) | Diagnostic accuracy study (feasibility) | (1) Tobii glasses eye-tracking while performing a task (making a cup of coffee) |
| Ku et al. (2020) | To investigate the determinants related to the ability to drive a motorized mobility scooter after a stroke. | Stroke (n = 29) | Cross-sectional study | (1) Colour Trails Test; (2) Reaction Time test; (3) Visual Acuity test; (4) Visual Field test |
| Laukkanen, Scheiman, and Hayes (2017) | To validate the instrument on a sample of adult TBI against a reference cohort of optometry students. | TBI (n = 107) | Cross-sectional study | (1) Brain Injury Vision Symptom Survey (BIVSS) |
| Leibovitch, Vasquez, Ebert, Beresford, and Black (2012) | To test and select an efficient, small battery of tests to address gaps in tests of neglect. Previous tests have shown to either fail to detect mild forms of neglect or are too lengthy to use at bedside. | Stroke (n = 224) | Cross-sectional study | (1) Sunnybrook Neglect Assessment Procedure (SNAP) |
| Luukkainen-Markkula, Tarkka, Pitkanen, Sivenius, and Hamalainen (2011) | To compare the severity of visual neglect in the conventional subtests of the Behavioural Inattention Test with the severity of neglect measured in the Catherine Bergego Scale in individual patients with hemi-spatial neglect. | Stroke (n = 17) | Cross-sectional study | (1) Conventional subtests of the Behavioural Inattention Test (BIT C); (2) Catherine Bergego Scale (CBS) |
| Malouin et al. (2007) | To examine the test-retest reliability of the Kinesthetic and Visual Imagery Questionnaire (KVIQ-20) and its short version (the KVIQ-10) in healthy subjects and subjects with stroke, investigate the internal consistency of both KVIQ versions, and explore the factorial structure of the two KVIQ versions. | Stroke (n = 19) | Diagnostic accuracy study (reliability) | (1) Kinesthetic Visual Imagery Questionnaire (KVIQ-20); (2) Kinesthetic Visual Imagery Questionnaire short version (the KVIQ-10) |
| Maruta, Suh, Niogi, Mukherjee, and Ghajar (2010) | To determine whether performance variability during predictive visual tracking can provide a screening measure for mild traumatic brain injury. | Concussion (n = 17) | Cross-sectional study | (1) Eye movements recorded binocularly with video-oculography device (EyeLink II, SR Research, Osgoode, Ontario, Canada) at 500-Hz sampling frequency. |
| Matthey, Donnelly, and Hextell (1993) | To examine the statistical background of the Rivermead Perceptual Assessment Battery (RPAB) on the interpretation of a patient's results and to identify potential weaknesses of the RPAB which may reduce its clinical usefulness. | Acquired brain injury (n = 51) | Cohort study (retrospective) | (1) Rivermead Perceptual Assessment Battery (RPAB) |
| Mattingley et al. (2004) | To examine attentional biases for judging the darker of two left–right mirror-reversed brightness gradients under conditions of free viewing (the greyscales task). | Stroke (n = 98) | Cross-sectional study | (1) Greyscales task |
| Maxton, Dineen, Padamsey, and Munshi (2013) | To perform a literature search that examines all aspects of post-stroke neglect assessment and treatment. | Stroke | Narrative review | (1) Gaze and Head Orientation; (2) Personal Neglect assessment; (3) Behavioural Inattention Test (BIT); (4) 'Clock face'; (5) Copy a Figure or Circle; (6) Line Bisection Test; (7) Writing assessment; (8) Bells test; (9) Catherine Bergego Scale (CBS) |
| Mazer, Sofer, Korner-Bitensky, and Gelinas (2001) | To examine the use of a visual attention analyzer in the evaluation and retraining of useful field of view in clients with stroke. | Stroke (n = 52) | Cross-sectional study (pilot) | (1) Useful field of view (UFOV) |
| Nijboer, Ten Brink, Kouwenhoven, and Visser-Meily (2014) | To investigate behavioural consequences at the level of basic activities of daily living of region-specific neglect, using the Catherine Bergego Scale (CBS). | Stroke (n = 118) | Cross-sectional study | (1) Catherine Bergego Scale (CBS) |
| Ogourtsova, Souza Silva, Archambault, and Lamontagne (2017) | To identify and appraise existing VR-based USN assessments; and to determine whether VR is more effective than conventional therapy. Assessment tools were critically appraised using standard criteria. | Stroke | Review (systematic) | N/A |
| Piscicelli, Nadeau, Barra, and Pérennou (2015) | To determine how many trials are required to assess visual vertical (VV) perception after stroke to better adapt assessment to patient abilities while maintaining good inter-trial reliability of the measure. | Stroke (n = 117) | Cohort study (prospective) | (1) Visual Vertical (VV) assessment |
| Politzer et al. (2017) | To assess the intra- and interrater reliability of a new rating scale for detecting the presence and degree of 5 oculomotor abnormalities after TBI. | Moderate to severe mechanical TBI (n = 11) | Diagnostic accuracy study (reliability) | (1) The Craig Hospital Eye Evaluation Rating Scale (CHEERS) |
| Potter et al. (2000) | To determine whether a computer-based method for recording standardized assessment of neglect identifies abnormalities in the ‘process’ as well as in the 'outcome’ of such tasks. | Stroke (n = 87) | Cohort study (controlled) | (1) Rivermead Behavioural Inattention Test (BIT); (2) computer recording of Line Bisection Test and Albert’s test |
| Punt et al. (2008) | To investigate the performance of patients with neglect when steering a power wheelchair on two tasks. | Stroke (n = 6) | Cross-sectional study | (1) Wheelchair Assessment Course (WAC); (2) Doorway Accuracy Test (DAT) |
| Razemba, Jacobs, and Franzsen (2017) | To determine the convergent validity of the Occupational Therapy Adult Perceptual Screening Test (OT-APST) with two other cognitive-perceptual tools used in South Africa. | Stroke (n = 32) | Cross-sectional study | (1) Occupational Therapy Adult Perceptual Screening Test (OT-APST); (2) Loewenstein Occupational Therapy Cognitive Assessment (LOTCA); (3) Dynamic Loewenstein Occupational Therapy Cognitive Assessment (DLOTCA); (4) Rivermead Perceptual Assessment Battery (RPAB) |
| Rorden et al. (2012) | To measure the severity of egocentric and allocentric aspects of neglect by using a novel continuous measure for allocentric neglect, in addition to a recently developed continuous measure for egocentric neglect. | Stroke (n = 36) | Cross-sectional study | (1) Defect Detection task (Ota et al., 2001); (2) Letter and Feature Cancellation tasks |
| Saviola et al. (2018) | To identify a short battery of neuropsychological tests with predictive value with regard to safe return to driving after sABI. | ABI (n = 127) | Cohort study (prospective) | (1) Attentive Matrices; (2) Trail Making Test (TMT A/B); (3) Bells test; (4) Spatial Span; (5) Digit Span; (6) Verbal Memory test; (7) Supra-Spatial Span; (8) Street's Completion test; (9) Abstract Reasoning test; (10) Rey Osterrieth Complex Figure; (11) Raven Progressive Matrix; (12) Wisconsin Card Sorting test; (13) Elithorn Perceptual Maze Test (PMT); (14) Verbal Fluency on Phonological Cue; (15) WAIS-R Performance IQ; (16) WAIS-R Verbal IQ |
| Stroke Engine (Figueiredo, 2011; Marvin, 2012; McDermott, 2012; Zeltzer, 2012; Zeltzer, 2008a, 2008b; Zeltzer & Menon, 2008a, 2008b, 2008c, 2008d, 2008e, 2008f; Zeltzer, 2010) | To provide resources and evidence-based recommendation for stroke assessment and treatment. | Stroke | Website | (1) Ontario Society of Occupational Therapists (OSOT) Perceptual Evaluation; (2) Motor-Free Visual Perception Test (MVPT); (3) Draw-a-man test; (4) Clock Drawing Test (CDT); (5) Double Letter Cancellation Test (DLCT); (6) Line Bisection Test; (7) Albert's Test; (8) Semi-Structured Scale for the Functional Evaluation of Hemi-inattention Evaluation; (9) Behavioural Inattention Test (BIT); (10) Catherine Bergego Scale (CBS); (11) Single Letter Cancellation Test (SLCT); (12) Colour Trails Test (CTT); (13) Trail Making Test (TMT) |
| Su, Chien, Cheng, and Lin (1995) | To investigate whether older subjects with brain damage score lower on the Test of Visual-Perceptual Skills (TVPS) than control subjects without brain damage matched for age and education, determine the demographic effects on test performance in both groups, and determine the capacity of TVPS in identifying Visual-perceptual deficits in adults with brain damage. | Stroke (n = 22) | Cross-sectional study | (1) Test of Visual-Perceptual Skills (TVPS) |
| Su et al. (2000) | To assess perceptual performances of patients with intracerebral hemorrhage (ICH) compared with those of ischemic patients early after stroke and to analyze the psychometric properties of three perceptual tests used in the study. | Stroke (n = 22) | Cross-sectional study | (1) Loewenstein Occupational Therapy Cognitive Assessment (LOTCA); (2) Rivermead Perceptual Assessment Battery (RPAB); (3) Motor-Free Visual Perception Test (MVPT) |
| Ten Brink, Visser-Meily, and Nijboer (2018) | To assess the feasibility of the Mobility Assessment Course (MAC), a visual search multitasks, to assess neglect, and its relationship with existing neglect tasks. | Stroke (n = 113) | Cross-sectional study | (1) Mobility Assessment Course (MAC) |
| Tippett, Alexander, Rizkalla, Sergio, and Black (2013) | To determine how visuospatial and visuomotor performance in chronic stroke patients compare to the performance of healthy participants. | Stroke (n = 9) | Cross-sectional study | (1) Computer-based visuomotor task (CbVM) |
| Titus, Gall, Yerxa, Roberson, and Mack (1991) | To determine to what extent a sample of 25 stroke patients would differ from normative samples on perceptual abilities, to explore relationships between particular perceptual tests and performance of daily living tasks with the use of a comprehensive battery of standardized tests available to occupational therapists, and to evaluate and compare the effectiveness of these assessments in identifying the deficits and abilities of this sample. | Stroke (n = 25) | Cross-sectional study | (1) Gross Visual Skills; (2) Adult Visual-Perceptual Assessment; (3) Manikin and Feature Profile subtests of the Arthur Point Scale of Performance tests; (4) Form H of Judgment of Line Orientation; (5) Bender Visual Motor Gestalt test; (6) Haptic Visual Discrimination test; (7) Block Design and Object Assembly subtests of the Wechsler Adult Intelligence Scale-Revised; (8) Test of Three-Dimensional Constructional Praxis (3rd ed) |
| Toglia and Cermak (2009) | To investigate the application of dynamic assessment in examining learning potential for adults with right hemisphere stroke and unilateral neglect. | Stroke (n = 20) | Randomized control trial | (1) Line Bisection Test; (2) Star Cancellation Test; (3) Picture Scanning subtest (subtest of BIT); (4) Object Search task |
| Tsirlin, Dupierrix, Chokron, Coquillart, and Ohlmann (2009) | To present past and ongoing research of virtual reality applications for unilateral neglect and discuss the existing problems and new directions for development. | Stroke | Review | N/A |
| Van der Stigchel and Nijboer (2018) | To investigate to what extent performance on standard neglect tests is dependent on the spatial bias, the aim of this study was to relate performance on the TOJ test to performance online bisection and cancellation tests. | Stroke (n = 73) | Cross-sectional study | (1) Temporal order judgement (TOJ) test; (2) Line Bisection Test; (3) Shape Cancellation test |
| Van Deusen (1988) | To summarize the research literature pertinent to the evaluation and treatment of unilateral neglect as it pertains to potential investigations by occupational therapists. | Stroke (n = 20) | Review | (1) Line Bisection Test; (2) Search-A-Word (SAW); (3) Speeded Reading of Word Lists (SRWL); (4) BIT |
| Wang, Sonoda, Hanamura, Okazaki, and Saitoh (2005) | To investigate the relationship between the bisection test and the severity of behavioural hemineglect and to verify if this test can predict the behavioural hemineglect. | Stroke (n = 30) | Cross-sectional study | (1) Line Bisection Test; (2) Line Re-Bisection Test |
| Warren (1990) | To examine the reliability and validity of five tests designed to measure visual scanning in the post-CVA adult patient. | Stroke (n = 23) | Diagnostic accuracy study (reliability and validity) | (1) Light Show Single; (2) Light Show Double; (3) Light Show Scanning; (4) Scan Board; (5) Design Copy test |
| Weightman, Radomski, Mashima, and Roth (2014) | To review in the literature assessment and treatment recommendations for mTBI. | mTBI | Book | (1) Test of near point convergence (NPC); (2) Eye Alignment test; (3) Developmental Eye Movement (DEM) test |
| Wetzel et al. (2018) | To investigate an analysis of eye movements, which may be more sensitive to neurologic dysfunction. | mTBI (n = 71) | Cross-sectional study | (1) Eye Tracking assessments via EyeLink 1000 at 500 Hz |
| Whitehouse et al. (2019) | To provide initial construct validity for this assessment measure by examining performance on the HVST in a sample of stroke patients in comparison to conventional and functional outcomes. | Stroke (n = 15) | Cross-sectional study | (1) Halifax Visual Scanning Test (HVST) |
| Whitney and Sparto (2019) | To provide the clinician with the most up-to-date knowledge related to eye movement abnormalities, screening measures, and evidence related to exercise interventions that are designed to enhance outcomes in persons after mTBI. | mTBI | Narrative Review | N/A |
| Yaretzky, Raviv, Netz, and Jacob (1995) | To address primary visual memory of stroke patients who function cognitively within the normal range of the MMSE, using the Gedachtnis Markaufsamkeit Test (GEMAT) visual memory test. | Stroke (n = 29) | Cross-sectional study | (1) Gedachtnis Markaufsamkeit Test (GEMAT) visuo-memory test |
| Zaninotto et al. (2017) | To compare visual memory performance via the Rey–Osterrieth Complex Figure (ROCF) test 6 and 12 months after diffuse axonal injury (DAI). | TBI (n = 40) | Cohort study (prospective) | (1) Rey-Osterrieh Complex Figure (ROCF) test |
| Zoltan (2007) | To provide a manual for the evaluation and treatment of the adult with acquired brain injury. | ABI | Book | (1) Pepper Visual Skills for Reading Test (VSRT); (2) Developmental Eye Movement test; (3) Dynamic Object Search test; (4) Near Point of Convergence (NPC); (5) Behavioural Inattention Test (BIT); (6) Formboard test; (7) Ayres' Figure-Ground Test; (8) LOTCA; (9) Ayres' Space Visualization test |

ABI, Acquired Brain Injury; sABI, Severe Acquired Brain Injury; TBI, Traumatic Brain Injury; mTBI, mild Traumatic Brain Injury; USN, Unilateral Spatial Neglect; CVA, Cerebrovascular Accident; RHD, Right Hemisphere Damage; LHD, Left Hemisphere Damage; MMSE, Mini Mental State Examination; GCS, Glasgow Coma Score; VR, Virtual Reality; US, United States.

References

Akinwuntan, A., Feys, H., De Weerdt, W., Baten, G., Arno, P., & Kiekens, C. (2006). prediction of driving after stroke: A prospective study. *Neurorehabilitation and Neural Repair, 20*(3), 417-423. doi:10.1177/1545968306287157

Akinwuntan, A., Devos, H., Feys, H., Verheyden, G., Baten, G., Kiekens, C., & De Weerdt, W. (2007). Confirmation of the accuracy of a short battery to predict fitness-to-drive of stroke survivors without severe deficits. *Journal of Rehabilitation Medicine, 39*(9), 698-702. doi:10.2340/16501977-0113

Akinwuntan, A., Feys, H., DeWeerdt, W., Pauwels, J., Baten, G., & Strypstein, E. (2002). Determinants of driving after stroke. *Archives of Physical Medicine and Rehabilitation, 83*(3), 334-341. doi:10.1053/apmr.2002.29662

Amesz, S., Tessari, A., Ottoboni, G., & Marsden, J. (2016). An observational study of implicit motor imagery using laterality recognition of the hand after stroke. *Brain Injury, 30*(8), 999-1004. doi:10.3109/02699052.2016.1147600

Appelros, P., Nydevik, I., Karlsson, G., Thorwalls, A., & Seiger, A. (2004). Recovery from unilateral neglect after right-hemisphere stroke. *Disability & Rehabilitation, 26*(8), 471-477. doi:10.1080/09638280410001663058

Azouvi, Marchal, Samuel, C., Morin, Renard, C., Louis-Dreyfus, A., . . . Bergego. (1996). Functional Consequences and Awareness of Unilateral Neglect: Study of an Evaluation Scale. *Neuropsychological Rehabilitation, 6*(2), 133-150. doi:10.1080/713755501

Azouvi, P., Bartolomeo, P., Beis, J., Perennou, D., Pradat-Diehl, P., & Rousseaux, M. (2006). A battery of tests for the quantitative assessment of unilateral neglect. *Restorative Neurology & Neuroscience, 24*(4), 273-285.

Azouvi, P., Samuel, C., Louis-Dreyfus, A., Bernati, T., Bartolomeo, P., Beis, J., . . . Rousseaux, M. (2002). Sensitivity of clinical and behavioural tests of spatial neglect after right hemisphere stroke. *Journal of Neurology, Neurosurgery & Psychiatry*, 160-166. doi:10.1136/jnnp.73.2.160

Bailey, M., Riddoch, M., & Crome, P. (2000). Evaluation of a test battery for hemineglect in elderly stroke patients for use by therapists in clinical practice. *Neurorehabilitation, 14*(3), 139-150. doi:10.3233/NRE-2000-14303

Barco, P., Wallendorf, M., Snellgrove, C., Ott, B., & Carr, D. (2014). Predicting road test performance in drivers with stroke. *American Journal of Occupational Therapy, 68*(2), 221-229. doi:10.5014/ajot.2014.008938

Barker-Collo, S., Feigin, V., Lawes, C., Senior, H., & Parag, V. (2010). Natural history of attention deficits and their influence on functional recovery from acute stages to 6 months after stroke. *Neuroepidemiology, 35*(4), 255-262. doi:10.1159/000319894

Basagni, B., De Tanti, A., Damora, A., Abbruzzese, L., Varalta, V., Antonucci, G., . . . Mancuso, M. (2017). The assessment of hemineglect syndrome with cancellation tasks: A comparison between the Bells test and the Apples test. *Neurological Sciences, 38*(12), 2171-2176. doi:10.1007/s10072-017-3139-7

Beis, J., Keller, C., Morin, N., Bartolomeo, P., Bernati, T., Chokron, S., . . . Azouvi, P. (2004). Right spatial neglect after left hemisphere stroke: Qualitative and quantitative study. *Neurology, 63*(9), 1600-1605. doi:10.1212/01.WNL.0000142967.60579.32

Bickerton, W., Samson, D., Williamson, J., & Humphreys, G. (2011). Separating forms of neglect using the Apples Test: Validation and functional prediction in chronic and acute stroke. *Neuropsychology, 25*(5), 567-580. doi:10.1037/a0023501

Blaylock, S., Warren, M., Yuen, H., & DeCarlo, D. (2016). Validation of a reading assessment for persons with homonymous hemianopia or quadrantanopia. *Archives of Physical Medicine & Rehabilitation, 97*(9), 1515-1519. doi:10.1016/j.apmr.2016.02.022

Bohannon, R. (2003). Evaluation and treatment of sensory and perceptual impairments following stroke. *Topics in Geriatric Rehabilitation, 19*(2), 87-97.

Brown, T., Mapleston, J., & Nairn, A. (2011). Convergent validity of the Occupational Therapy Adult Perceptual Screening Test (OT-APST) with two other cognitive-perceptual tests. *The British Journal of Occupational Therapy, 74*(12), 562-572. doi:10.4276/030802211X13232584581416

Brown, T., Mapleston, J., & Nairn, A. (2012). Can cognitive and perceptual standardized test scores predict functional performance in adults diagnosed with stroke? A pilot study. *Physical & Occupational Therapy in Geriatrics, 30*(1), 31-44. doi:10.3109/02703181.2011.652348

Butler, A., Cazeaux, J., Fidler, A., Jansen, J., Lefkove, N., Gregg, M., . . . Wolf, S. (2012). The Movement Imagery Questionnaire-Revised, Second Edition (MIQ-RS) is a reliable and valid tool for evaluating motor imagery in stroke populations. *Evidence-Based Complementary & Alternative Medicine: eCAM, 2012*, 497289. doi:10.1155/2012/497289

Calvanio, R., Williams, R., Burke, D., Mello, J., Lepak, P., Al-Adawi, S., & Shah, M. (2004). Acquired brain injury, visual attention, and the useful field of view test: A pilot study. *Archives of Physical Medicine & Rehabilitation, 85*(3), 474-478. doi:10.1016/S0003-9993(03)00469-6

Cassidy, T., Lewis, S., & Gray, C. (1998). Recovery from visuospatial neglect in stroke patients. *Journal of Neurology, Neurosurgery & Psychiatry, 64*(4), 555-557. doi:10.1136/jnnp.64.4.555

Cate, Y., & Richards, L. (2000). Relationship between performance on tests of basic visual functions and visual-perceptual processing in persons after brain injury. *American Journal of Occupational Therapy, 54*(3), 326-334. doi:10.5014/ajot.54.3.326

Cermak, S., & Hausser, J. (1989). The Behavioral Inattention Test for unilateral visual neglect: A critical review. *Physical & Occupational Therapy in Geriatrics, 7*(3), 43-53. doi:10.1300/J148v07n03_04

Chiu, E., Wu, W., Chou, C., Yu, M., & Hung, J. (2016). Test-retest reliability and minimal detectable change of the Test of Visual Perceptual Skills-Third Edition in patients with stroke. *Archives of Physical Medicine & Rehabilitation, 97*(11), 1917-1923. doi:10.1016/j.apmr.2016.04.023

Chiu, E., Yu, M., Wu, W., Chou, C., Hung, J., & Chen, P. (2019). Validation of the Test of Visual Perceptual Skills-Third Edition in patients with stroke. *Disability & Rehabilitation, 41*(1), 104-109. doi:10.1080/09638288.2017.1378389

Cooke, D., McKenna, K., & Fleming, J. (2005). Development of a standardized occupational therapy screening tool for visual perception in adults. *Scandinavian Journal of Occupational Therapy, 12*(2), 59-71. doi:10.1080/11038120410020683-1

Cooke, D., McKenna, K., Fleming, J., & Darnell, R. (2005). The reliability of the Occupational Therapy Adult Perceptual Screening Test (OT-APST). *British Journal of Occupational Therapy, 68*(11), 509-517. doi:10.1177/03080226050681105

Cooke, D., McKenna, K., Fleming, J., & Darnell, R. (2006a). Construct and ecological validity of the Occupational Therapy Adult Perceptual Screening Test (OT-APST). *Scandinavian Journal of Occupational Therapy, 13*(1), 49-61. doi:10.1080/11038120500363014

Cooke, D., McKenna, K., Fleming, J., & Darnell, R. (2006b). Criterion validity of the Occupational Therapy Adult Perceptual Screening Test (OT-APST). *Scandinavian Journal of Occupational Therapy, 13*(1), 38-48. doi:10.1080/11038120500363006

Donnelly, S. (2002). The Rivermead Perceptual Assessment Battery: Can it predict functional performance? *Australian Occupational Therapy Journal, 49*(2), 71-81. doi:10.1046/j.1440-1630.2002.00308.x

Dunlap, P., Mucha, A., Smithnosky, D., Whitney, S., Furman, J., Collins, M., . . . Sparto, P. (2020). The Gaze Stabilization Test following concussion. *Journal of the American Academy of Audiology*, Advance online publication. doi:10.3766/jaaa.18015

Erez, A., Katz, N., Ring, H., & Soroker, N. (2009). Assessment of spatial neglect using computerised feature and conjunction visual search tasks. *Neuropsychological Rehabilitation, 19*(5), 677-695. doi:10.1080/09602010802711160

Figueiredo, S. (2011). Behavioral Inattention Test (BIT). Retrieved from <https://www.strokengine.ca/en/assess/bit/>

George, S., Clark, M., & Crotty, M. (2008). Validation of the Visual Recognition Slide Test with stroke: A component of the New South Wales occupational therapy off-road driver rehabilitation program. *Australian Occupational Therapy Journal, 55*(3), 172-179. doi:10.1111/j.1440-1630.2007.00699.x

Greve, K., Lindberg, R., Bianchini, K., & Adams, D. (2000). Construct validity and predictive value of the Hooper Visual Organization Test in stroke rehabilitation. *Applied Neuropsychology, 7*(4), 215-222. doi:10.1207/S15324826AN0704_3

Halligan, P., Wilson, B., & Cockburn, J. (1990). A short screening test for visual neglect in stroke patients. *International Disability Studies, 12*(3), 95-99. doi:10.3109/03790799009166260

Harlowe, D., & Van Deusen, J. (1984). Construct validation of the St. Marys CVA evaluation: Perceptual measures. *American Journal of Occupational Therapy, 38*(3), 184-186. doi:10.5014/ajot.38.3.184

Hartman-Maeir, A., Erez, A., Ratzon, N., Mattatia, T., & Weiss, P. (2008). The validity of the Color Trail Test in the pre-driver assessment of individuals with acquired brain injury. *Brain Injury, 22*(13/14), 994-998. doi:10.1080/02699050802491305

Hunfalvay, M., Roberts, C., Murray, N., Tyagi, A., Kelly, H., & Bolte, T. (2019). Horizontal and vertical self-paced saccades as a diagnostic marker of traumatic brain injury. *Concussion, 4*(1), CNC60. doi:10.2217/cnc-2019-0001

Hunfalvay, M., Roberts, C., Murray, N., Tyagi, A., Barclay, K., Bolte, T., . . . Carrick, F. (2020). Vertical smooth pursuit as a diagnostic marker of traumatic brain injury. *Concussion, 5*(1), CNC69. doi:10.2217/cnc-2019-0013

Jolly, N., Macfarlane, A., & Heard, R. (2013). Towards gaining the best information about vision to assist the recovery of a patient with stroke. *Strabismus, 21*(2), 145-149. doi:10.3109/09273972.2013.787633

Kettunen, J., Nurmi, M., Dastidar, P., & Jehkonen, M. (2012). Recovery from visual neglect after right hemisphere stroke: does starting point in cancellation tasks change after 6 months? *Clinical Neuropsychologist, 26*(2), 305-320. doi:10.1080/13854046.2011.648213

Koiava, N., Ong, Y., Brown, M., Acheson, J., Plant, G., & Leff, A. (2012). A 'web app' for diagnosing hemianopia. *Journal of Neurology, Neurosurgery & Psychiatry, 83*(12), 1222-1224. doi:10.1136/jnnp-2012-302270

Kontos, A., Deitrick, J., Collins, M., & Mucha, A. (2017). Review of vestibular and oculomotor screening and concussion rehabilitation. *Journal of Athletic Training, 52*(3), 256-261. doi:10.4085/1062-6050-51.11.05

Korner-Bitensky, N., Mazer, B., Sofer, S., Gelinas, I., Meyer, M., Morrison, C., . . . White, M. (2000). Visual testing for readiness to drive after stroke: A multicenter study. *American Journal of Physical Medicine & Rehabilitation, 79*(3), 253-317. doi:10.1097/00002060-200005000-00007

Kortman, B., & Nicholls, K. (2016). Assessing for unilateral spatial neglect using eye-tracking glasses: A feasibility study. *Occupational Therapy In Health Care, 30*(4), 344-355. doi:10.1080/07380577.2016.1208858

Ku, F., Chen, W., Chen, M., Tung, S., Chen, T., & Tsai, C. (2020). The determinants of motorized mobility scooter driving ability after a stroke. *Disability & Rehabilitation*, 1-10. doi:10.1080/09638288.2020.1748125

Laukkanen, H., Scheiman, M., & Hayes, J. (2017). Brain injury vision symptom survey (BIVSS) questionnaire. *Optometry and Vision Science, 94*(1), 43-50. doi:10.1097/OPX.0000000000000940

Leibovitch, F., Vasquez, B., Ebert, P., Beresford, K., & Black, S. (2012). A short bedside battery for visuoconstructive hemispatial neglect: Sunnybrook Neglect Assessment Procedure (SNAP). *Journal of Clinical & Experimental Neuropsychology: Official Journal of the International Neuropsychological Society, 34*(4), 359-368. doi:10.1080/13803395.2011.645016

Luukkainen-Markkula, R., Tarkka, I., Pitkanen, K., Sivenius, J., & Hamalainen, H. (2011). Comparison of the Behavioural Inattention Test and the Catherine Bergego Scale in assessment of hemispatial neglect. *Neuropsychological Rehabilitation, 21*(1), 103-116. doi:10.1080/09602011.2010.531619

Malouin, F., Richards, C., Jackson, P., Lafleur, M., Durand, A., & Doyon, J. (2007). The Kinesthetic and Visual Imagery Questionnaire (KVIQ) for assessing motor imagery in persons with physical disabilities: a reliability and construct validity study. *Journal of Neurologic Physical Therapy, 31*(1), 20-29. doi:10.1097/NPT.0000260567.24122.64

Maruta, J., Suh, M., Niogi, S., Mukherjee, P., & Ghajar, J. (2010). Visual tracking synchronization as a metric for concussion screening. *The Journal of Head Trauma Rehabilitation, 25*(4), 293-305. doi:10.1097/HTR.0b013e3181e67936

Marvin, K. (2012). Trail Making Test (TMT). Retrieved from <https://www.strokengine.ca/en/assess/tmt/>

Matthey, S., Donnelly, S., & Hextell, D. (1993). The clinical usefulness of the Rivermead Perceptual Assessment Battery: Statistical considerations. *The British Journal of Occupational Therapy, 56*(10), 365-370. doi:10.1177/030802269305601003

Mattingley, J., Berberovic, N., Corben, L., Slavin, M., Nicholls, M., & Bradshaw, J. (2004). The greyscales task: A perceptual measure of attentional bias following unilateral hemispheric damage. *Neuropsychologia, 42*(3), 387-394. doi:10.1016/j.neuropsychologia.2003.07.007

Maxton, C., Dineen, R., Padamsey, R., & Munshi, S. (2013). Don't neglect 'neglect'- an update on post stroke neglect. *International Journal of Clinical Practice, 67*(4), 369-378. doi:10.1111/ijcp.12058

Mazer, B., Sofer, S., Korner-Bitensky, N., & Gelinas, I. (2001). Use of the UFOV to evaluate and retrain visual attention skills in clients with stroke: a pilot study. *American Journal of Occupational Therapy, 55*(5), 552-557. doi:10.5014/ajot.55.5.552

McDermott, A. (2012). Catherine Bergego Scale (CBS). Retrieved from <https://www.strokengine.ca/en/assess/cbs/>

Nijboer, T., Ten Brink, A., Kouwenhoven, M., & Visser-Meily, J. (2014). Functional assessment of region-specific neglect: Are there differential behavioural consequences of peripersonal versus extrapersonal neglect? *Behavioural Neurology, 2014*, 526407. doi:10.1155/2014/526407

Ogourtsova, T., Souza Silva, W., Archambault, P., & Lamontagne, A. (2017). Virtual reality treatment and assessments for post-stroke unilateral spatial neglect: A systematic literature review. *Neuropsychological Rehabilitation, 27*(3), 409-454. doi:10.1080/09602011.2015.1113187

Piscicelli, C., Nadeau, S., Barra, J., & Pérennou, D. (2015). Assessing the visual vertical: How many trials are required? *BMC Neurology, 15*(1), 1-5. doi:10.1186/s12883-015-0462-6

Politzer, T., Berryman, A., Rasavage, K., Snell, L., Weintraub, A., & Gerber, D. (2017). The Craig Hospital Eye Evaluation Rating Scale (CHEERS). *Pm & R, 9*(5), 477-482. doi:10.1016/j.pmrj.2016.08.032

Potter, J., Deighton, T., Patel, M., Fairhurst, M., Guest, R., & Donnelly, N. (2000). Computer recording of standard tests of visual neglect in stroke patients. *Clinical Rehabilitation, 14*(4), 441-446. doi:10.1191/0269215500cr344oa

Punt, T., Kitadono, K., Hulleman, J., Humphreys, G., Riddoch, M., Punt, T., . . . Riddoch, M. (2008). From both sides now: Crossover effects influence navigation in patients with unilateral neglect. *Journal of Neurology, Neurosurgery & Psychiatry, 79*(4), 464-466. doi:10.1136/jnnp.2007.139832

Razemba, F., Jacobs, L., & Franzsen, D. (2017). Convergent validity of the Occupational Therapy Adult Perceptual Screening Test (OT-APST) with two other cognitive-perceptual tools in a South African context. *South African Journal of Occupational Therapy, 47*(2), 3-10. doi:10.17159/2310-3833/2017/v47n2a2

Rorden, C., Hjaltason, H., Fillmore, P., Fridriksson, J., Kjartansson, O., Magnusdottir, S., & Karnath, H. (2012). Allocentric neglect strongly associated with egocentric neglect. *Neuropsychologia, 50*(6), 1151-1157. doi:10.1016/j.neuropsychologia.2012.03.031

Saviola, D., De Tanti, A., Conforti, J., Posteraro, L., Manfredini, A., Bagattini, C., & Basagni, B. (2018). Safe return to driving following severe acquired brain injury: Role of a short neuropsychological assessment. *European Journal of Physical & Rehabilitation Medicine., 54*(5), 717-723. doi:10.23736/S1973-9087.17.04905-X

Su, C., Chien, T., Cheng, K., & Lin, Y. (1995). Performance of older adults with and without cerebrovascular accident on the test of visual-perceptual skills. *American Journal of Occupational Therapy, 49*(6), 491-499. doi:10.5014/ajot.49.6.491

Su, C., Chang, J., Chen, H., Su, C., Chien, T., & Huang, M. (2000). Perceptual differences between stroke patients with cerebral infarction and intracerebral hemorrhage. *Archives of Physical Medicine & Rehabilitation, 81*(6), 706-714. doi:10.1016/S0003-9993(00)90097-2

Ten Brink, A., Visser-Meily, J., & Nijboer, T. (2018). Dynamic assessment of visual neglect: The mobility assessment course as a diagnostic tool. *Journal of Clinical and Experimental Neuropsychology, 40*(2), 161-172. doi:10.1080/13803395.2017.1324562

Tippett, W., Alexander, L., Rizkalla, M., Sergio, L., & Black, S. (2013). True functional ability of chronic stroke patients. *Journal of Neuroengineering & Rehabilitation, 10*, 20. doi:10.1186/1743-0003-10-20

Titus, M., Gall, N., Yerxa, E., Roberson, T., & Mack, W. (1991). Correlation of perceptual performance and activities of daily living in stroke patients. *American Journal of Occupational Therapy, 45*(5), 410-418. doi:10.514/ajot.45.5.410

Toglia, J., & Cermak, S. (2009). Dynamic assessment and prediction of learning potential in clients with unilateral neglect. *American Journal of Occupational Therapy, 63*(5), 569-579. doi:10.5014/ajot.63.5.569

Tsirlin, I., Dupierrix, E., Chokron, S., Coquillart, S., & Ohlmann, T. (2009). Uses of virtual reality for diagnosis, rehabilitation and study of unilateral spatial neglect: Review and analysis. *Cyberpsychology & Behavior, 12*(2), 175-181. doi:10.1089/cpb.2008.0208

Van der Stigchel, S., & Nijboer, T. (2018). Temporal order judgements as a sensitive measure of the spatial bias in patients with visuospatial neglect. *Journal of Neuropsychology, 12*(3), 427-441. doi:10.1111/jnp.12118

Van Deusen, J. (1988). Unilateral neglect: Suggestions for research by occupational therapists. *American Journal of Occupational Therapy, 42*(7), 441-448. doi:10.5014/ajot.42.7.441

Wang, Q., Sonoda, S., Hanamura, M., Okazaki, H., & Saitoh, E. (2005). Line bisection and rebisection: the crossover effect of space location. *Neurorehabilitation & Neural Repair, 19*(2), 84-92. doi:10.1177/1545968305274661

Warren, M. (1990). Identification of visual scanning deficits in adults after cerebrovascular accident. *American Journal of Occupational Therapy, 44*(5), 391-399. doi:10.5014/ajot.44.5.391

Weightman, M., Radomski, M., Mashima, P., & Roth, C. (2014). *Mild Traumatic Brain Injury Rehabilitation Toolkit*. Borden Institute.

Wetzel, P., Lindblad, A., Raizada, H., James, N., Mulatya, C., Kannan, M., . . . Weaver, L. (2018). Eye tracking results in postconcussive syndrome versus normative participants. *Investigative Ophthalmology & Visual Science, 59*(10), 4011-4019. doi:10.1167/iovs.18-23815

Whitehouse, C., Green, J., Giles, S., Rahman, R., Coolican, J., & Eskes, G. (2019). Development of the Halifax Visual Scanning Test: A new measure of visual-spatial neglect for personal, peripersonal, and extrapersonal space. *Journal of the International Neuropsychological Society*, 1-11. doi:10.1017/S135561771900002X

Whitney, S., & Sparto, P. (2019). Eye movements, dizziness, and mild traumatic brain injury (mTBI): A topical review of emerging evidence and screening measures. *Journal of Neurologic Physical Therapy, 43*, S31-S36. doi:10.1097/NPT.0000000000000272

Yaretzky, A., Raviv, S., Netz, Y., & Jacob, T. (1995). Primary visual memory of stroke patients. *Disability & Rehabilitation, 17*(6), 293-297. doi:10.3109/09638289509166649

Zaninotto, A., Vicentini, J., Solla, D., Silva, T., Guirado, V., Feltrin, F., . . . Paiva, W. (2017). Visuospatial memory improvement in patients with diffuse axonal injury (DAI): A 1-year follow-up study. *Acta Neuropsychiatrica, 29*(1), 35-42. doi:10.1017/neu.2016.29

Zeltzer, L., & Poulin, V. (2012). Color Trails Test (CTT). Retrieved from https://www.strokengine.ca/en/assess/ctt/

Zeltzer, L. (2008a). Motor-Free Visual Perception Test (MVPT). Retrieved from <https://www.strokengine.ca/en/assess/mvpt/>

Zeltzer, L. (2008b). Ontario Society of Occupational Therapists (OSOT) Perceptual Evaluation. Retrieved from <https://www.strokengine.ca/en/assess/osot/>

Zeltzer, L., & Menon, A. (2008a). Clock Drawing Test (CDT). Retrieved from <https://www.strokengine.ca/en/assess/cdt/>

Zeltzer, L., & Menon, A. (2008b). Double Letter Cancellation Test (DLTC). Retrieved from <https://www.strokengine.ca/en/assess/dlct/>

Zeltzer, L., & Menon, A. (2008c). Draw-A-Man Test. Retrieved from <https://www.strokengine.ca/en/assess/damt/>

Zeltzer, L., & Menon, A. (2008d). Line Bisection Test. Retrieved from <https://www.strokengine.ca/en/assess/lbt/>

Zeltzer, L., & Menon, A. (2008e). Semi-Structured Scale for the Functional Evaluation of Hemi-inattention. Retrieved from <https://www.strokengine.ca/en/assess/sssfeh/>

Zeltzer, L., & Menon, A. (2008f). Single Letter Cancellation Test (SLCT). Retrieved from <https://www.strokengine.ca/en/assess/slct/>

Zeltzer, L., & Menon, A. (2010). Albert's Test. Retrieved from <https://www.strokengine.ca/en/assess/at/>

Zoltan, B. (2007). *Vision, perception, and cognition: A manual for the evaluation and treatment of the adult with acquired brain injury, fourth edition (4^th^ ed.)*. Thorofare, NJ: Slack Incorporated.
