# Supplementary material for: Vision Evaluation Tools for Adults With Acquired Brain Injury: A Scoping Review
Source: Can J Occup Ther. 2021 Oct 18;88(4):340–51. doi: 10.1177/00084174211042955 (PMC8640270; doi:10.1177/00084174211042955)
Supplement: sj-docx-3-cjo-10.1177_00084174211042955 - Supplemental material for Vision Evaluation Tools for Adults With Acquired Brain Injury: A Scoping Review [file sj-docx-3-cjo-10.1177_00084174211042955.docx]

Appendix C

Summary of the tools

| **# of Studies** | **Name of the evaluation tool (s)** | **What areas of vision are measured?** | **Domains, Sub-scales, Items** | **Population** | **By whom the tool(s) is/are used?** | **Time to Administer** | **How does the tool(s) work?** |
| --- | --- | --- | --- | --- | --- | --- | --- |
|  |  |  |  |  |  |  |  |
| n = 1 (Titus, Gall, Yerxa, Roberson, & Mack, 1991) | Adult Visual-Perceptual Assessment | Visual-perceptual skills | (1) Figure-Ground; (2) Form Constancy; (3) Position in Space; (4) Depth Perception; (5) Visual Body Scheme; (6) Spatial Relations | Stroke | OT | Not reported | Standardized clinical test with some portion paper-and-pencil test |
| n = 8 (Appelros, Nydevik, Karlsson, Thorwalls, & Seiger, 2004; Calvanio et al., 2004; McDermott, 2012; Potter et al., 2000; Toglia & Cermak, 2009; Zeltzer & Menon, 2008a, 2008c, 2010) | Albert's Test | Visual neglect | N/A | ABI | Neuropsychologist and OT | 5 minutes | Standardized paper-and-pencil clinical test |
| n = 2 (Basagni et al., 2017; Bickerton, Samson, Williamson, & Humphreys, 2011) | Apple's Test/Apple Cancellation Test/Apples subtest of Birmingham University Cognitive Screen (BUCS) | Peripersonal, allocentric and egocentric spatial neglect | N/A | ABI | OT | 5 minutes | Standardized paper-and-pencil clinical test |
| n = 2 (Harlowe & Van Deusen, 1984; Zoltan, 2007) | Ayres' Figure-Ground Test | Figure-ground perception | N/A | ABI | Not reported | Not reported | Paper-and-pencil clinical test |
| n = 1 (Zoltan, 2007) | Ayres' Space Visualization Test | Spatial relations | N/A | ABI | Not reported | Not reported | Paper-and-pencil clinical test |
| n = 2 (Bailey, Riddoch, & Crome, 2000; Zeltzer & Menon, 2008c) | Baking Tray Test (BTT) | Visuo-spatial neglect | N/A | Stroke | Not reported | Not reported | Standardized clinical test |
| n = 2 (Azouvi et al., 2006; Azouvi et al., 2002) | Batterie d'Évaluation de la Néglicence Spatiale (BEN) | Extrapersonal neglect, gaze orientation, personal neglect, awareness of motor and visual deficits, visual extinction and hemianopia | (1) Bell's test; (2) Figure Copying; (3) Clock Drawing; (4) Line Bisection; (5) Overlapping Figures test; (6) Reading; (7) Writing; (8) Assessment of Gaze Orientation and Personal Neglect; (9) Assessment of Related Disorders (anosognosia and extinction) | Stroke | Not reported | Not reported | Combination of paper-and-pencil test and clinical observation |
| n = 1 (Beis et al., 2004) | Preliminary Assessment Battery of Anosognosia and Visual Extinction | Anosognosia, visual extinction, hemianopia gaze orientation, personal neglect, and extrapersonal neglect | (1) Assessment of Gaze Orientation and Personal Neglect; (2) Bells test; (3) Figure Copying; (4) Clock Drawing; (5) Line Bisection | Stroke | Not reported | 1 hour | Paper-and-pencil test and clinical observation |
| n = 1 (Saviola et al., 2018) | Battery | Tests (2), (3), (8), (10) and (15) measure: Visual gnostic function and spatial exploration | (1) Attentive Matrices; (2) Trail Making Test (TMT A/B); (3) Bells test; (4) Spatial Span; (5) Digit Span; (6) Verbal Memory test; (7) Supra-Spatial Span; (8) Street’s Completion test; (9); Abstract Reasoning test; (10) Rey-Osterrieth Complex Figure; (11) Raven Progressive Matrix; (12) Wisconsin Card Sorting test; (13) Wlithorn Perceptual Maze Test (PMT); (14) Verbal Fluency on Phonological Cue; (15) WAIS-R Performance IQ; (16) WAIS-R Verbal IQ | Severe ABI, TBI, stroke and other non-traumatic neurological disorders | Not reported | Not reported | Paper-and-pencil clinical test |
| n = 1 (Rorden et al., 2012) | Battery | Discrimination of egocentric and allocentric neglect and object-based deficits | (1) Defect Detection task (Ota, Fujii, Suzuki, Fukatsu, & Yamadori, 2001); (2) Letter and Feature Cancellation tasks (Weintraub, 1985) scored using the center of cancellation (CoC) measure and software (Rorden & Karnath, 2010) | Stroke | Neuropsychologist | Not reported | Paper-and-pencil clinical test |
| n = 1 (Azouvi et al., 1996) | Battery | Visual neglect | (1) Daisy Drawing; (2) Ogden’s Scene; (3) Line Cancellation; (4) Bells test; (5) Reading task | Stroke | OT | Not reported | Paper-and-pencil clinical test |
| n = 1 (Barco, Wallendorf, Snellgrove, Ott, & Carr, 2014) | Battery | Visual-spatial abilities and visual scanning | (1) Snellgrove Maze Task (SMT); (2) Trail Making Test Part A | Stroke | OT and OT assistant | 5 minutes | Paper-and-pencil test |
| n = 1 (Akinwuntan et al., 2006) | Battery | (1) Far vision; (2) Acuity; (3) Ability to recognize objects in motion; (4) Perceptual organization and visuospatial inattention; (5) Degree of reduction in the useful field of view by evaluating the speed of processing, divided and selective attention of performance using visual discrimination, and localization tasks; (6) Divided attention, visual scanning, incompatibility, visual field, and visual neglect; (7) Attention, concentration, and executive reasoning abilities; (8) On-road test measures driving abilities | (1) Monocular Visual Acuity test; (2) Binocular Visual Acuity test; (3) Kinetic Vision test; (4) Figure of Rey; (5) Useful Field of View (UFOV); (6) 5 out of 6 tests in the Test for Attentional Performance (TAP) battery; (7) Stroke Driver Screening Assessment (SDSA); (8) Checklist adapted from the Test Ride for Investigating Practical Fitness to Drive (TRIP) | Stroke | Psychologist and driving assessment expert | 2-3 hours | Clinical test and clinical observation |
| n = 1 (Bailey et al., 2000) | Battery | Tests (1) to (4) measure Visuo-spatial neglect, (5) Representational neglect, (6) Directional hypokinesia or pre-motor neglect, and (7) Personal neglect | (1) Star Cancellation Test (SCT); (2) Line Bisection; (3) Copy-a-Daisy; (4) Baking Tray Task (BTT); (5) Draw-a-Clock; (6) Exploratory Motor (EM) task; (7) Personal Neglect (PN) test | Stroke | Not reported | 20 minutes | Paper-and-pencil clinical test |
| n = 16 (Appelros et al., 2004; Azouvi et al., 2006; Bailey et al., 2000; Bohannon, 2003; Cassidy, Lewis, & Gray, 1998; Cermak & Hausser, 1989; Erez, Katz, Ring, & Soroker, 2009; Figueiredo, 2011; Halligan, Wilson, & Cockburn, 1990; Kettunen, Nurmi, Dastidar, & Jehkonen, 2012; Luukkainen-Markkula, Tarkka, Pitkanen, Sivenius, & Hamalainen, 2011; Maxton, Dineen, Padamsey, & Munshi, 2013; McDermott, 2012; Potter et al., 2000; Van Deusen, 1988; Whitehouse et al., 2019) BITC n = 2 (Kettunen et al., 2012; Luukkainen-Markkula et al., 2011) | Behavioral Inattention Test (BIT); Rivermead Behavioral Inattention Test; Conventional Behavioral Inattention Test (BIT C) | Neglect, visual attention, visuo-spatial neglect in reaching space, visual and behavioral neglect, visual spatial functioning, USN, visual starting point, visual sided bias, and visual search strategy | (1) Line Crossing; (2) Letter Cancellation; (3) Star Cancellation; (4) Figure and Shape Copying; (5) Line Bisection; (6) Representational Drawing; (7) Picture Scanning; (8) Telephone Dialling; (9) Menu Reading; (10) Article Reading; (11) Telling and Setting the Time; (12) Coin Sorting; (13) Address and Sentence Copying; (14) Map Navigation; (15) Card Sorting | Stroke | All | 45 minutes to 1 hour | Standardized paper-and-pencil clinical test |
| n = 10 (Azouvi et al., 1996; Azouvi et al., 2006; Azouvi et al., 2002; Barker-Collo, Feigin, Lawes, Parag, & Senior, 2010; Basagni et al., 2017; Beis et al., 2004; Bickerton et al., 2011; Kortman & Nicholls, 2016; Maxton et al., 2013; Saviola et al., 2018) | Bells test | USN in the peripersonal space | N/A | Stroke | Neuropsychologist and OT | 5 minutes | Paper-and-pencil clinical test |
| n = 1 (Titus et al., 1991) | Bender Visual Motor Gestalt Test | Visuomotor skills | N/A | Stroke | OT | Not reported | Standardized paper-and-pencil test |
| n = 1 (Tippett, Alexander, Rizkalla, Sergio, & Black, 2013) | Benton Judgement of Line Orientation (BLO) | Visuospatial | N/A | Stroke | Not reported | Not reported | Clinical test |
| n = 1 (Titus et al., 1991) | Block Design and Object Assembly subtest of the Wechsler Adult Intelligence Scale-Revised (WAIS-R) | Apraxia | N/A | Stroke | Supervision by a psychologist when employed by an OT | Not reported | Standardized clinical test |
| n = 1 (Laukkanen, Scheiman, & Hayes, 2017) | Brain Injury Vision Symptom Survey (BIVSS) | Visual symptoms (e.g. eyesight clarity, visual comfort, diplopia, depth perception, dry eye, peripheral vision, light sensitivity, and reading) | 28 items in the symptom checklist | TBI | Self-administered, interpreted by optometrist | Not reported | Self-report |
| n = 11 (Amesz, Tessari, Ottoboni, & Marsden, 2016; Azouvi et al., 1996; Azouvi et al., 2006; Azouvi et al., 2002; Erez et al., 2009; Luukkainen-Markkula et al., 2011; Maxton et al., 2013; McDermott, 2012; Nijboer, Ten Brink, Kouwenhoven, & Visser-Meily, 2014; Ten Brink, Visser-Meily, & Nijboer, 2018; Wang, Sonoda, Hanamura, Okazaki, & Saitoh, 2005) | Catherine Bergego Scale (CBS) | Performance in personal, peripersonal, and extrapersonal space, in perceptual, representational and motor domains, USN, and self-awareness of behavioral neglect (anosognosia) | 10 real life situations (e.g., grooming, dressing, and wheelchair driving) | Stroke | Nurse, OT and neuropsychologist | 30 minutes | Standardized paper-and-pencil clinical test and clinical observation |
| n = 1 (Jolly, Macfarlane, & Heard, 2013) | Checklist for Vision Problems Post Stroke | Visual field and visual acuity | Items: symptoms, history, medication, glasses, distance vision, near vision, pupil defects, eye movement defects, ocular deviation, reduced CNP, field loss, neglect, nystagmus, and face or lid droop | Stroke | Orthoptist | Not reported | Questionnaire, clinical observation and clinical test |
| n = 9 (Azouvi et al., 2006; Azouvi et al., 2002; Bailey et al., 2000; Barco et al., 2014; Beis et al., 2004; Leibovitch, Vasquez, Ebert, Beresford, & Black, 2012; Maxton et al., 2013; Tippett et al., 2013; Zeltzer & Menon, 2008a) | Clock Drawing Test (CDT) | Extrapersonal neglect | N/A | Stroke | All | 1-2 minute(s) | Paper-and-pencil test |
| n = 3 (Hartman-Maeir, Erez, Ratzon, Mattatia, & Weiss, 2008; Kortman & Nicholls, 2016; Zeltzer & Poulin, 2012) | Color Trails Test (CTT) | Sustained and divided attention, mental flexibility, visual spatial skills, and motor speed | 2 subtests (CTT1 and CTT2); 4 versions of the CTT (A, B, C and D) | Stroke | OT | 3-8 minutes | Standardized paper-and-pencil clinical test |
| n = 1 (Tippett et al., 2013) | Computer-based Visuomotor Task (CbVM) (adapted from (William J Tippett & Sergio, 2006) | Visuospatial and visuomotor abilities | 2 elements recorded to create total reaction time (RT): movement times (MT) and initiation time (IT) | Stroke | Not reported | 15-20 minutes | Computerized test |
| n = 1 (Erez et al., 2009) | Computerised visual search test and training programme (VISSTA – Visual Spatial Search Task) | Neglect, attentional bias, feature and conjunction visual search modes | N/A | Stroke | OT | Not reported | Computerized clinical test |
| n = 1 (Politzer et al., 2017) | Craig Hospital Eye Evaluation Rating Scale (CHEERS) | Eye movement abnormalities, smooth pursuit, saccades, convergence, vestibular ocular reflex (VOR), fixation and nystagmus | (1) Smooth Pursuit; (2) Saccades; (3) VOR; (4) Vergence; (5) Fixation | TBI | Administered by OT, but recorded and rated by neuro-optometrist andophthalmologist | 5-7 minutes | Clinical test and clinical observation |
| n = 1 (Warren, 1990) | Design Copy Test (Copy house, Clock, Flower, and Diamond drawings) | Visual scanning, visual closure, visual memory, spatial relationships, and visual discrimination | N/A | Stroke | OT | 5 minutes | Paper-and-pencil clinical test |
| n = 2 (Weightman, Radomski, Mashima, & Roth, 2014; Zoltan, 2007) | Developmental Eye Movement Test (DEM) | Saccadic eye movements | N/A | ABI | All | Not reported | Clinical observational |
| n = 2 (Brown, Mapleston, & Nairn, 2011, 2012) | Developmental Test of Visual Perception – Adolescent and Adult (DTVP-A) | Visual-motor integration and motor-free visual perceptual abilities | (1) Figure-Ground; (2) Visual-Motor Search; (3) Visual Closure; (4) Visual-Motor Speed; (5) Form Constancy; (6) General Visual Perception | Stroke | OT | 20 minutes | Paper-and-pencil test |
| n = 1 (Punt et al., 2008) | Doorway Accuracy Test (DAT) | USN, Indoor navigation with motorized wheelchair | N/A | Stroke | Not reported | 5-10 minutes | Observed functional task |
| n = 1 (Zeltzer, 2008b) | Double Letter Cancellation Test (DLCT) | USN in the near extrapersonal space | N/A | Stroke | All rehabilitation therapist (no training needed) | 5 minutes | Paper-and-pencil clinical test |
| n = 1 (Zeltzer & Menon, 2008b) | Draw-A-Man test | Unilateral Spatial Neglect (USN) in the personal and extrapersonal space, and anosognosia | N/A | Stroke | Not reported | Less than 5 minutes | Paper-and-pencil clinical test |
| n = 1 (Weightman et al., 2014) | Eye Alignment test | Binocular vision | N/A | TBI | All | Not reported | Clinical test and clinical observation |
| n = 1 (Maruta, Suh, Niogi, Mukherjee, & Ghajar, 2010) | Eye movements recorded binocularly with video-oculography device (EyeLink II, SR Research, Osgoode, Ontario,Canada) at 500-Hz sampling frequency | Visual tracking, gaze trajectory, and eye position | N/A | mTBI | Ophthalmologist | Not reported | Clinical test with measurement device (video-oculography device, EyeLink II) |
| n = 1 (Wetzel et al., 2018) | Eye tracking assessments via EyeLink 1000 at 500 Hz | Saccadic and smooth pursuit | (1) 2-point; (2) Circular; (3) Horizontal & Vertical Ramp; (4) Horizontal & Vertical Step; (5) Reading; (6) Memory guided on and off; (7) Antisaccade | mTBI | Technician trained in ophthalmology | Not reported | Measurement device |
| n = 1 (Titus et al., 1991) | Form H of Judgment of Line Orientation | Visuospatial orientation | N/A | Stroke | OT | Not reported | Standardized clinical test |
| n = 2 (Weightman et al., 2014; Zoltan, 2007) | Formboard Test | Form discrimination | N/A | ABI | Not reported | Not reported | Paper-and-pencil clinical test |
| n = 1 (Dunlap et al., 2018) | Gaze Stabilization Test (GST) | Vestibular ocular reflex | N/A | Concussion | PT | Not reported | Clinical test |
| n = 1 (Yaretzky, Raviv, Netz, & Jacob, 1995) | Gedachtnis Markaufsamkeit Test (GEMAT) visual-memory test | Primary visual memory | 96 geometric figures of a simple structure (24 used as stimulus) | Stroke | Not reported | 10 minutes | Clinical test |
| n = 1 (Mattingley et al., 2004) | Greyscales task | Attentional bias | N/A | Stroke | All | 5 minutes | Clinical test |
| n = 1 (Titus et al., 1991) | Gross Visual Skills | Gross visual skills | (1) Visual Attentiveness; (2) Ocular Pursuits; (3) Field Cut Testing; (4) Alternating Simultaneous Stimuli; (5) Line Bisection | Stroke | OT | Not reported | Standardized clinical test with some portion paper-and-pencil test |
| n = 1 (Whitehouse et al., 2019) | Halifax Visual Scanning Test (HVST) | Visual spatial neglect (Personal, Peripersonal and Extrapersonal space) | (1) Shirt; (2) Paper; (3) Wall | Stroke | Not reported | 10 minutes | Clinical test |
| n = 1 (Titus et al., 1991) | Haptic Visual Discrimination Test | Agnosia | (1) Shape; (2) Size; (3) Texture; (4) Configuration | Stroke | OT | Not reported | Standardized clinical test |
| n = 1 (Greve, Lindberg, Bianchini, & Adams, 2000) | Hooper Visual Organization Test (HVOT) | Visual perception | 30 items (line drawings to mentally assemble and identify) | Stroke | Not reported | Not reported | Paper-and-pencil test |
| n = 1 (Dunlap et al., 2018) | Immediate Post-Concussion Assessment and Cognitive Test (ImPACT) | Verbal memory, visual memory, visual motor processing speed, and reaction time | N/A | Concussion | Neuropsychologist | Not reported | Self-report |
| n = 1 (Malouin et al., 2007) | Kinesthetic and Visual Imagery Questionnaire (KVIQ-20)/the KVIQ-10 | Motor imagery in the visual and the kinesthetic dimensions | KVIQ-20: Visual and kinesthetic subscales, 20 items (10 movements in each of the visual and kinesthetic subscales); KVIQ-10 includes 10 items (5 movements in each of the visual and kinesthetic subscales) | Stroke | PT and OT | KVIQ-20: 45 minutes; KVIQ-10: 23 minutes | Questionnaire |
| n = 2 (Kontos, Deitrick, Collins, & Mucha, 2017; Whitney & Sparto, 2019) | King-Devick test | Saccadic eye movements | N/A | Concussion | Neuro-optometrist, neuro-ophthalmologist, OT and PT | 2 minutes | Clinical test |
| n = 1 (Warren, 1990) | Light Show Device Tests (Light Show Single, Light Show Double, Light Show Scanning) | Visual scanning, visual closure, visual memory, spatial relationships, and visual discrimination | N/A | Stroke | OT | 20 minutes | Observed functional task using measurement device |
| n = 12 (Azouvi et al., 2002; Bailey et al., 2000; Beis et al., 2004; Bohannon, 2003; Potter et al., 2000; Ten Brink et al., 2018; Tsirlin, Dupierrix, Chokron, Coquillart, & Ohlmann, 2009; Van der Stigchel & Nijboer, 2018; Van Deusen, 1988; Wang et al., 2005; Zeltzer & Menon, 2008c, 2010) | Line bisection Test | USN and visual attention | N/A | Stroke | Any therapist, OT, neuropsychologist and doctor | 5 minutes | Standardized paper-and-pencil clinical test and computerized test(Potter et al., 2000) |
| n = 5 (n = 3 LOTCA (Cooke, McKenna, & Fleming, 2005; Su et al., 2000; Zoltan, 2007); n = 2 LOTCA-2 (Cooke, McKenna, Fleming, & Darnell, 2006b; Razemba, Jacobs, & Franzsen, 2017); n = 1 DLOTCA (Razemba et al., 2017); n = 1 LOTCA-G (Cooke, McKenna, & Fleming, 2005) | Loewenstein Occupational Therapy Cognitive Assessment (LOTCA); LOTCA-II; & Dynamic Loewenstein Occupational Therapy Cognitive Assessment (DLOTCA) | Visual perception | **LOTCA**  (20 items, 4 domains): (1) Orientation; (2) Perception; (3) Visuomotor Organization; (4) Thinking Operations. **LOTCA-II**  (26 items, 6 domains): (1) Orientation; (2) Visual Perception; (3) Spatial Perception; (4) Praxis; (5) Visuomotor Organization; (6) Thinking Operations. **DLOTCA**  (28 items, 7 domains): (1) Orientation; (2) Awareness; (3) Visual Perception; (4) Spatial Perception; (5) Praxis; (6) Visuomotor Construction; (7) Thinking Operations. **LOTCA-G**  (23 items, 6 domains): (1) Orientation; (2) Perception; (3) Praxis; (4) Visuomotor Organization; (5) Thinking Operations; (6) Memory | ABI | OT | **LOTCA:** 45 minutes-2 hours **LOTCA-G:** 30-45 minutes | Clinical test |
| n = 1 (Titus et al., 1991) | Manikin and Feature Profile subtests of the Arthur Point Scale of Performance Tests | Body scheme/Body parts | N/A | Stroke | OT | Not reported | Standardized clinical test |
| n = 1 (Ten Brink et al., 2018) | Mobility Assessment Course (MAC) | Visuospatial neglect, wayfinding | 24 targets to find and report on corridor walls | Stroke | Not reported | 2-10 minutes | Clinical test and functional task |
| n = 4 (Cate & Richards, 2000; Korner-Bitensky et al., 2000; Su et al., 2000; Zeltzer, 2008a) | Motor-Free Visual Perception Test (MVPT) and MVPT – 3rd Edition (MVPT-3) | Visual perception independent of motor ability, basic functional screening battery: visual acuity, visual field integrity, oculomotor function, visual scanning and attention | (1) Visual Discrimination; (2) Figure-Ground Discrimination; (3) Spatial Relationships; (4) Visual Closure; (5) Visual Memory | Stroke and TBI | OT | MVPT: 15-20 minutes; MVPT-3: 30-40 minutes | Standardized paper-and-pencil clinical test |
| n = 1 (Butler et al., 2012) | Movement Imagery Questionnaire-Revised, Second Edition (MIQ-RS) | Mental imagery | 7 visual and 7 kinesthetic items | Stroke | Not reported | 25–30 minutes | Self-report |
| n = 7 (Brown et al., 2011, 2012; Cooke, McKenna, & Fleming,2005; Cooke, McKenna, Fleming, & Darnell, 2005, 2006a, 2006b; Razemba et al., 2017) | Occupational Therapy Adult Perceptual Screening Test (OT-APST) | Agnosia, visuospatial skills including body scheme and neglect, constructional skills, apraxia, and acalculia | OT-APST (25 items, 7 subscales): (1) Agnosia (Colour Naming, Object Name, Figure-Ground, Shape Constancy, Reading); (2) Body Scheme (Body Parts-Self, Body Parts-Therapist, Left/Right on Self, Directions/Positions); (3) USN (Clock, House, Hand Writing, Reading, Telling Time); (4) Constructional Skills (Clock, House, 2D Construction, 3D Construction); (5) Functional Skills; (6) Apraxia (Facial Gesture, Wave Right Hand, Wave Left Hand, Stapler Manipulation, Pen Use, Handwriting); (7) Acalculia (Acalculia) | Stroke | OT | 20-25 minutes | Standardized paper-and-pencil clinical test |
| n = 1 (Zeltzer, 2008b) | Ontario Society of Occupational Therapists (OSOT) Perceptual Evaluation | Perceptual impairment | Original OSOT (28 subtests); Revised OSOT (18 subtests); both evaluate 6 domains: (1) Sensation; (2) Scanning; (3) Apraxia; (4) Body Awareness; (5) Spatial Relations; (6) Visual Agnosia | TBI or stroke | OT | 90 minutes | Paper-and-pencil clinical test |
| n = 1 (Koiava et al., 2012) | Read-Right | Visual field | N/A | ABI | Not reported | 5 minutes | Computerized clinical test |
| n = 5 (Akinwuntan et al., 2006; Akinwuntan et al., 2007; Akinwuntan et al., 2002; Saviola et al., 2018; Zaninotto et al., 2017) | Rey–Osterrieth Complex Figure ROCF Test/ The Figure of Rey | Visuospatial memory | Visuospatial recall memory, visuospatial recognition memory, response bias, processing speed, and visuospatial constructional ability | ABI | Certified psychologist, driving assessment expert, neurologist, OT, and neuropsychologist | 45 minutes | Standardized paper-and-pen clinical test |
| n = 2 (Hunfalvay et al., 2019; Hunfalvay et al., 2020) | RightEye oculomotor tests (Horizontal Saccades and Vertical Saccades test) | Horizontal and vertical saccades | N/A | TBI | Not reported | Not reported | Measurement device |
| n = 1 (Hunfalvay et al., 2020) | RightEye Vertical Smooth Pursuit test (RightEye, LLC, MD, USA) | Smooth pursuits | Variance and smooth pursuit percentage | TBI | Neurologist and neuro-optometrist | Not reported | Computerized clinical test with measurement device (eye-tracking equipment) |
| n = 6 (Bohannon, 2003; Cooke, McKenna, & Fleming, 2005; Donnelly, 2002; Matthey, Donnelly, & Hextell, 1993; Razemba et al., 2017; Su et al., 2000) | Rivermead Perceptual Assessment Battery (RPAB) | Visual perception | RPAB (16 items, 8 categories): (1) Form Constancy; (2) Colour Constancy; (3) Sequencing; (4) Object Completion; (5) Figure Ground Discrimination; (6) Body Image; (7) Inattention; (8) Spatial Awareness | Stroke | OT | 45 minutes-2 hours | Paper-and-pencil clinical test |
| n = 2 (Warren, 1990; Weightman et al., 2014) | Scan Board Test | Visual scanning, visual search, and visual discrimination | N/A | Stroke | OT | 5 minutes | Clinical observation (using a visual scanning board) |
| n = 1 (Van Deusen, 1988) | Search-A-Word (SAW) | USN, reading | N/A | Stroke | OT | Not reported | Clinical test |
| n = 1 (Zeltzer, 2008a) | Semi-Structured Scale for the Functional Evaluation of Hemi-inattention Evaluation | USN in both the personal and extrapersonal space | Personal neglect and extrapersonal neglect subscales | Stroke | Not reported | 20 minutes | Clinical observation (of functional tasks) |
| n = 1 (Akinwuntan et al., 2007) | Short assessment battery | (1) Visuospatial abilities, attention, organization and problem-solving skills; (2) Visual neglect; and (3) Driving abilities | (1) Figure of Rey; (2) Visual Neglect test, part of the Test for Attentional Performance (TAP) battery; (3) On-road test | Stroke | Neurologist, OT and neuropsychologist | Not reported | Standardized paper-and-pencil clinical test and clinical observation, observed functional task (for the on-road test) |
| n = 1 (Zeltzer & Menon, 2008a) | Single Letter Cancellation Test (SLCT) | USN in the near extrapersonal space and visual scanning deficits | N/A | Stroke | Not reported | 5 minutes | Paper-and-pencil clinical test |
| n = 1 (Van Deusen, 1988) | Speeded Reading of Word Lists (SRWL) | USN, left spatial hemi-imperception and lateral eye movement efficiency, reading | 4 scores obtained: (1) Anchoring errors; (2) Scanning effectiveness; (3) Proportion of success with side-placed versus center-placed words; (4) Periphery monitoring | Stroke | OT | Not reported | Computerized clinical test |
| n = 1 (Harlowe & Van Deusen, 1984) | St. Marys CVA Evaluation Battery include (1) Body Scheme; (2) Figure Ground; (3) Position in Space; (4) Spacial Relations; (5) Stereognosis | Visual perception | (1) Body Scheme (St. Marys Body Scheme Test 3-piece body puzzle, St. Marys Body Scheme Test 6-piece puzzle, MacDonald Draw-a-Person Test); (2) Figure Ground (Ayres Figure-ground Perception Test [items 1 through 6 of the Southern California Sensory Integration Test (SCSIT)], Frostig Figure Ground Test [adapted]); (3) Position in Space (Ayres Position in Space Test [items 1 through 8 of the SCSIT]); (4) Spacial Relations (St. Marys Spacial Relations Test); (5) Stereognosis (Coin Subtest) | Stroke | OT | Not reported | Paper-and-pencil clinical tests |
| n = 9 (Bailey et al., 2000; Bickerton et al., 2011; Figueiredo, 2011; Mattingley et al., 2004; McDermott, 2012; Toglia & Cermak, 2009; Zeltzer & Menon, 2008c, 2010; Zoltan, 2007) | Star Cancellation Test (SCT) | Visuo-spatial neglect | N/A | Stroke | OT | Not reported as an individual test | Standardized paper-and-pencil clinical test |
| n = 2 (Leibovitch et al., 2012; Whitehouse et al., 2019) | Sunnybrook Neglect Assessment Procedure (SNAP) | Visual attention and neglect | (1) Drawing/Copying of a Clock and Daisy; (2) Line Bisection; (3) Line Cancellation; (4) Shape Cancellation | Stroke | Not reported | Not reported | Paper-and-pencil test |
| n = 1 (Van der Stigchel & Nijboer, 2018) | Temporal Order Judgement (TOJ) test | Neglect and spatial bias | N/A | Stroke | Not reported | Not reported | Computerized clinical test |
| n = 2 (Weightman et al., 2014; Zoltan, 2007) | Test of near point convergence (NPC) | Convergence | N/A | TBI | All | 5 minutes | Clinical test and clinical observation |
| n = 1 (Titus et al., 1991) | Test of Three-Dimensional Constructional Praxis (3rd edition) | Apraxia | N/A | Stroke | OT | Not reported | Standardized clinical test |
| n = 3 (Chiu, Wu, Chou, Yu, & Hung, 2016; Chiu et al., 2019; Su, Chien, Cheng, & Lin, 1995) TVPS, n = 1 (Su et al., 1995) and TVPS-3, n = 2 (Chiu et al., 2016; Chiu et al., 2019) | Test of Visual Perceptual Skills (TVPS) and Test of Visual Perceptual Skills - Third Edition (TVPS-3) | Visual perception | (1) Visual Discrimination; (2) Visual Memory; (3) Spatial Relationships; (4) Form Constancy; (5) Sequential Memory; (6) Figure-Ground; (7) Visual Closure | Stroke | Trained OT (2-hour training) | 40 minutes | Clinical test |
| n = 1 (Kortman & Nicholls, 2016) | Tobii glasses eye-tracking while performing a task (making a cup of coffee) | Gaze direction and fixation of object | N/A | Stroke | OT | Not reported | Measurement device |
| n = 6 (Barco et al., 2014; Barker-Collo et al., 2010; Calvanio et al., 2004; Marvin, 2012; Saviola et al., 2018; Tippett et al., 2013) | Trail Making Tests (TMT) | Both Part A & B: Motor function, attention, visual screening/scanning and processing speed, and overall cognitive functioning; only Part A: rote memory; only Part B: executive functioning | 2 subtests (TMT A/B), and 4 versions (A, B, C and D)**.** | ABI | Neuropsychologist, OT, OT assistant, driver rehabilitation specialist, driving instructor and SLP | 5-10 minutes | Standardized paper-and-pencil clinical test |
| n = 7 (Akinwuntan et al., 2006; Akinwuntan et al., 2007; Akinwuntan et al., 2002; Barco et al., 2014; Calvanio et al., 2004; Hartman-Maeir et al., 2008; Mazer, Sofer, Korner-Bitensky, & Gelinas, 2001) | Useful Field Of View (UFOV) | Useful field of view, degree of reduction in the useful field of view by evaluating the speed of processing, divided and selective attention of performance using visual discrimination, and localization tasks | (1) speed of simple visual data processing; (2) divided attention; (3) selective attention | ABI | Neurologist, OT and OT assistant, neuropsychologist, driver rehabilitation specialist and expert, SLP, certified psychologist | 15 minutes | Paper-and-pencil clinical test |
| n = 2 (Kontos et al., 2017; Whitney & Sparto, 2019) | Vestibular/Ocular-Motor Screening (VOMS) tool | Smooth pursuit, saccades, convergence, VOR, and visual motion sensitivity | (1) Smooth pursuit; (2) Horizontal and Vertical saccades; (3) Convergence; (4) Horizontal and Vertical VOR; (5) Visual motion sensitivity | Concussion | Neuro-optometrist, neuro-ophthalmologist, OT and PT | 5-10 minutes | Clinical test |
| n = 1 (Ogourtsova, Souza Silva, Archambault, & Lamontagne, 2017) | Virtual Wheelchair Navigation Skills (Using VR headset motorized wheelchair, and treadmill) | Near and far-extrapersonal USN, motorized wheelchair navigation skills. | N/A | Stroke | OT | Not reported | Computerized and observed functional task |
| n = 1 (George, Clark, & Crotty, 2008) | Visual Recognition Slide Test (VRST) | Visual memory, scanning, speed of information processing, spatial orientation and depth perception | 18 slides projected | Stroke | OT (For on-road: driving instructor and driver-trained OT) | Not reported | Clinical test, clinical observation and observed functional task (on-road assessment) |
| n = 1 (George et al., 2008) | Visual Scanning Analyser (VSA) | Extent to which the participant scans or neglects his or her visual fields and scanning ability | N/A | Stroke | OT | Not reported | Clinical test |
| n = 2 (Blaylock, Warren, Yuen, & DeCarlo, 2016; Zoltan, 2007) | Visual Skills for Reading Test (VSRT) or Pepper Test | Visual-perceptual skill required for reading | (1) Visual word recognition ability; (2) Saccadic and return sweep eye movement control; (3) How well the client can position the central scotoma without obscuring the field of view necessary for reading | Patients with homonymous hemianopia or quadrantanopia | Trained OT | 10-15 minutes | Standardized clinical test |
| n = 1 (Piscicelli, Nadeau, Barra, & Pérennou, 2015) | Visual vertical (VV) assessment/visual vertical (VV) measurement/Visual estimation of the vertical (VV) test | Visual vertical perception | N/A | Stoke | Not reported | Not reported | Clinical observation |
| n = 1 (Ogourtsova et al., 2017) | VR Lateralized Attention Test (VRLAT) | Functional task within near and far-extrapersonal and USN, Wayfinding | N/A | Stroke | Neuropsychologist and OT | Not reported | Computerized and observed functional task |
| n = 1 (Ogourtsova et al., 2017) | VR-DiSTRO includes (1) VR-Star Cancellation (SCT); (2) VR-Line Bisection (LBT); (3) VR-Visual Extinction (EXT); (4) VR-Baking Tray Task (BTT) | Near space USN | N/A | Stroke | Neuropsychologist and OT | 15 minutes | Paper-and-pencil clinical test within VR immersive environment, with computerized tracking and scoring |

R, Right; L, Left; OT, Occupational Therapist; BIT, Behavioural Inattention Test; ABI, Acquired Brain Injury; N/A, Not Applicable; LH, Left Hemisphere; WAIS-R, Wechsler Adult Intelligence Scale-Revised; IQ, Intelligence Quotient; TBI, Traumatic Brain Injury; RH, Right Hemisphere; CNP, Convergence Near Point; mTBI, mild traumatic brain injury; cTBI, chronic traumatic brain injury; SD, Standard Deviation; PT, Physical Therapist; CVA, Cerebral Vascular Accident; SLP, Speech Language Pathologist; VR, Virtual Reality; USN, Unilateral Spatial Neglect.

References

Akinwuntan, A., Feys, H., De Weerdt, W., Baten, G., Arno, P., & Kiekens, C. (2006). Prediction of driving after stroke: A prospective study. *Neurorehabilitation and Neural Repair, 20*(3), 417-423. doi:10.1177/1545968306287157

Akinwuntan, A., Devos, H., Feys, H., Verheyden, G., Baten, G., Kiekens, C., & De Weerdt, W. (2007). Confirmation of the accuracy of a short battery to predict fitness-to-drive of stroke survivors without severe deficits. *Journal of Rehabilitation Medicine, 39*(9), 698-702. doi:10.2340/16501977-0113

Akinwuntan, A., Feys, H., DeWeerdt, W., Pauwels, J., Baten, G., & Strypstein, E. (2002). Determinants of driving after stroke. *Archives of Physical Medicine and Rehabilitation, 83*(3), 334-341. doi:10.1053/apmr.2002.29662

Amesz, S., Tessari, A., Ottoboni, G., & Marsden, J. (2016). An observational study of implicit motor imagery using laterality recognition of the hand after stroke. *Brain Injury, 30*(8), 999-1004. doi:10.3109/02699052.2016.1147600

Appelros, P., Nydevik, I., Karlsson, G., Thorwalls, A., & Seiger, A. (2004). Recovery from unilateral neglect after right-hemisphere stroke. *Disability & Rehabilitation, 26*(8), 471-477. doi:10.1080/09638280410001663058

Azouvi, Marchal, Samuel, C., Morin, Renard, C., Louis-Dreyfus, A., . . . Bergego. (1996). Functional consequences and awareness of unilateral neglect: Study of an evaluation scale. *Neuropsychological Rehabilitation, 6*(2), 133-150. doi:10.1080/713755501

Azouvi, P., Bartolomeo, P., Beis, J. M., Perennou, D., Pradat-Diehl, P., & Rousseaux, M. (2006). A battery of tests for the quantitative assessment of unilateral neglect. *Restorative Neurology & Neuroscience, 24*(4), 273-285.

Azouvi, P., Samuel, C., Louis-Dreyfus, A., Bernati, T., Bartolomeo, P., Beis, J., . . . Rousseaux, M. (2002). Sensitivity of clinical and behavioural tests of spatial neglect after right hemisphere stroke. *Journal of Neurology, Neurosurgery & Psychiatry*, 160-166. doi:10.1136/jnnp.73.2.160

Bailey, M., Riddoch, M., & Crome, P. (2000). Evaluation of a test battery for hemineglect in elderly stroke patients for use by therapists in clinical practice. *Neurorehabilitation, 14*(3), 139-150. doi:10.3233/NRE-2000-14303

Barco, P., Wallendorf, M., Snellgrove, C., Ott, B., & Carr, D. (2014). Predicting road test performance in drivers with stroke. *American Journal of Occupational Therapy, 68*(2), 221-229. doi:10.5014/ajot.2014.008938

Barker-Collo, S., Feigin, V., Lawes, C., Parag, V., & Senior, H. (2010). Attention deficits after incident stroke in the acute period: Frequency across types of attention and relationships to patient characteristics and functional outcomes. *Topics in Stroke Rehabilitation, 17*(6), 463-476. doi:10.1310/tsr1706-463

Basagni, B., De Tanti, A., Damora, A., Abbruzzese, L., Varalta, V., Antonucci, G., . . . Mancuso, M. (2017). The assessment of hemineglect syndrome with cancellation tasks: A comparison between the Bells test and the Apples test. *Neurological Sciences, 38*(12), 2171-2176. doi:10.1007/s10072-017-3139-7

Beis, J., Keller, C., Morin, N., Bartolomeo, P., Bernati, T., Chokron, S., . . . Azouvi, P. (2004). Right spatial neglect after left hemisphere stroke: Qualitative and quantitative study. *Neurology, 63*(9), 1600-1605. doi:10.1212/01.WNL.0000142967.60579.32

Bickerton, W., Samson, D., Williamson, J., & Humphreys, G. (2011). Separating forms of neglect using the Apples test: Validation and functional prediction in chronic and acute stroke. *Neuropsychology, 25*(5), 567-580. doi:10.1037/a0023501

Blaylock, S., Warren, M., Yuen, H., & DeCarlo, D. (2016). Validation of a reading assessment for persons with homonymous hemianopia or quadrantanopia. *Archives of Physical Medicine and Rehabilitation, 97*(9), 1515-1519. doi:10.1016/j.apmr.2016.02.022

Bohannon, R. (2003). Evaluation and treatment of sensory and perceptual impairments following stroke. *Topics in Geriatric Rehabilitation, 19*(2), 87-97.

Brown, T., Mapleston, J., & Nairn, A. (2011). Convergent validity of the Occupational Therapy Adult Perceptual Screening Test (OT-APST) with two other cognitive-perceptual tests. *The British Journal of Occupational Therapy, 74*(12), 562-572. doi:10.4276/030802211X13232584581416

Brown, T., Mapleston, J., & Nairn, A. (2012). Can cognitive and perceptual standardized test scores predict functional performance in adults diagnosed with stroke? A pilot study. *Physical & Occupational Therapy in Geriatrics, 30*(1), 31-44. doi:10.3109/02703181.2011.652348

Butler, A., Cazeaux, J., Fidler, A., Jansen, J., Lefkove, N., Gregg, M., . . . Wolf, S. (2012). The Movement Imagery Questionnaire-Revised, Second Edition (MIQ-RS) is a reliable and valid tool for evaluating motor imagery in stroke populations. *Evidence-Based Complementary & Alternative Medicine: eCAM, 2012*, 497289. doi:10.1155/2012/497289

Calvanio, R., Williams, R., Burke, D., Mello, J., Lepak, P., Al-Adawi, S., & Shah, M. (2004). Acquired brain injury, visual attention, and the useful field of view test: A pilot study. *Archives of Physical Medicine & Rehabilitation, 85*(3), 474-478. doi:10.1016/S0003-9993(03)00469-6

Cassidy, T., Lewis, S., & Gray, C. (1998). Recovery from visuospatial neglect in stroke patients. *Journal of Neurology, Neurosurgery & Psychiatry, 64*(4), 555-557. doi:10.1136/jnnp.64.4.555

Cate, Y., & Richards, L. (2000). Relationship between performance on tests of basic visual functions and visual-perceptual processing in persons after brain injury. *American Journal of Occupational Therapy, 54*(3), 326-334. doi:10.5014/ajot.54.3.326

Cermak, S., & Hausser, J. (1989). The Behavioral Inattention Test for unilateral visual neglect: A critical review. *Physical & Occupational Therapy in Geriatrics, 7*(3), 43-53. doi:10.1300/J148v07n03_04

Chiu, E., Wu, W., Chou, C., Yu, M., & Hung, J. (2016). Test-retest reliability and minimal detectable change of the Test of Visual Perceptual Skills-Third Edition in patients with stroke. *Archives of Physical Medicine & Rehabilitation, 97*(11), 1917-1923. doi:10.1016/j.apmr.2016.04.023

Chiu, E., Yu, M., Wu, W., Chou, C., Hung, J., & Chen, P. (2019). Validation of the Test of Visual Perceptual Skills-Third Edition in patients with stroke. *Disability & Rehabilitation, 41*(1), 104-109. doi:10.1080/09638288.2017.1378389

Cooke, D., McKenna, K., & Fleming, J. (2005). Development of a standardized occupational therapy screening tool for visual perception in adults. *Scandinavian Journal of Occupational Therapy, 12*(2), 59-71. doi:10.1080/11038120410020683-1

Cooke, D., McKenna, K., Fleming, J., & Darnell, R. (2005). The reliability of the Occupational Therapy Adult Perceptual Screening Test (OT-APST). *British Journal of Occupational Therapy, 68*(11), 509-517. doi:10.1177/03080226050681105

Cooke, D., McKenna, K., Fleming, J., & Darnell, R. (2006a). Construct and ecological validity of the Occupational Therapy Adult Perceptual Screening Test (OT-APST). *Scandinavian Journal of Occupational Therapy, 13*(1), 49-61. doi:10.1080/11038120500363014

Cooke, D., McKenna, K., Fleming, J., & Darnell, R. (2006b). Criterion validity of the Occupational Therapy Adult Perceptual Screening Test (OT-APST). *Scandinavian Journal of Occupational Therapy, 13*(1), 38-48. doi:10.1080/11038120500363006

Donnelly, S. (2002). The Rivermead Perceptual Assessment Battery: Can it predict functional performance? *Australian Occupational Therapy Journal, 49*(2), 71-81. doi:10.1046/j.1440-1630.2002.00308.x

Dunlap, P., Mucha, A., Smithnosky, D., Whitney, S., Furman, J., Collins, M., . . . Sparto, P. (2018). The gaze stabilization test following concussion. *Journal of the American Academy of Audiology,* Advance online publication. doi:10.3766/jaaa.18015

Erez, A., Katz, N., Ring, H., & Soroker, N. (2009). Assessment of spatial neglect using computerised feature and conjunction visual search tasks. *Neuropsychological Rehabilitation, 19*(5), 677-695. doi:10.1080/09602010802711160

Figueiredo, S. (2011). Behavioral Inattention Test (BIT). Retrieved from <https://www.strokengine.ca/en/assess/bit/>

George, S., Clark, M., & Crotty, M. (2008). Validation of the visual recognition slide test with stroke: A component of the New South Wales occupational therapy off-road driver rehabilitation program. *Australian Occupational Therapy Journal, 55*(3), 172-179. doi:10.1111/j.1440-1630.2007.00699.x

Greve, K., Lindberg, R., Bianchini, K., & Adams, D. (2000). Construct validity and predictive value of the Hooper Visual Organization Test in stroke rehabilitation. *Applied Neuropsychology, 7*(4), 215-222. doi:10.1207/S15324826AN0704_3

Halligan, P., Wilson, B., & Cockburn, J. (1990). A short screening test for visual neglect in stroke patients. *International Disability Studies, 12*(3), 95-99. doi:10.3109/03790799009166260

Harlowe, D., & Van Deusen, J. (1984). Construct validation of the St. Marys CVA evaluation: Perceptual measures. *American Journal of Occupational Therapy, 38*(3), 184-186. doi:10.5014/ajot.38.3.184

Hartman-Maeir, A., Erez, A., Ratzon, N., Mattatia, T., & Weiss, P. (2008). The validity of the Color Trail Test in the pre-driver assessment of individuals with acquired brain injury. *Brain Injury, 22*(13/14), 994-998. doi:10.1080/02699050802491305

Hunfalvay, M., Roberts, C., Murray, N., Tyagi, A., Kelly, H., & Bolte, T. (2019). Horizontal and vertical self-paced saccades as a diagnostic marker of traumatic brain injury. *Concussion, 4*(1), CNC60. doi:10.2217/cnc-2019-0001

Hunfalvay, M., Roberts, C., Murray, N., Tyagi, A., Barclay, K., Bolte, T., . . . Carrick, F. (2020). Vertical smooth pursuit as a diagnostic marker of traumatic brain injury. *Concussion, 5*(1), CNC69. doi:10.2217/cnc-2019-0013

Jolly, N., Macfarlane, A., & Heard, R. (2013). Towards gaining the best information about vision to assist the recovery of a patient with stroke. *Strabismus, 21*(2), 145-149. doi:10.3109/09273972.2013.787633

Kettunen, J., Nurmi, M., Dastidar, P., & Jehkonen, M. (2012). Recovery from visual neglect after right hemisphere stroke: Does starting point in cancellation tasks change after 6 months? *Clinical Neuropsychologist, 26*(2), 305-320. doi:10.1080/13854046.2011.648213

Koiava, N., Ong, Y., Brown, M., Acheson, J., Plant, G., & Leff, A. (2012). A 'web app' for diagnosing hemianopia. *Journal of Neurology, Neurosurgery & Psychiatry, 83*(12), 1222-1224. doi:10.1136/jnnp-2012-302270

Kontos, A., Deitrick, J., Collins, M., & Mucha, A. (2017). Review of vestibular and oculomotor screening and concussion rehabilitation. *Journal of Athletic Training, 52*(3), 256-261. doi:10.4085/1062-6050-51.11.05

Korner-Bitensky, N., Mazer, B., Sofer, S., Gelinas, I., Meyer, M., Morrison, C., . . . White, M. (2000). Visual testing for readiness to drive after stroke: A multicenter study. *American Journal of Physical Medicine & Rehabilitation, 79*(3), 253-317. doi:10.1097/00002060-200005000-00007

Kortman, B., & Nicholls, K. (2016). Assessing for unilateral spatial neglect using eye-tracking glasses: A feasibility study. *Occupational Therapy In Health Care, 30*(4), 344-355. doi:10.1080/07380577.2016.1208858

Laukkanen, H., Scheiman, M., & Hayes, J. (2017). Brain Injury Vision Symptom Survey (BIVSS) questionnaire. *Optometry and Vision Science, 94*(1), 43-50. doi:10.1097/OPX.0000000000000940

Leibovitch, F., Vasquez, B., Ebert, P., Beresford, K., & Black, S. (2012). A short bedside battery for visuoconstructive hemispatial neglect: Sunnybrook Neglect Assessment Procedure (SNAP). *Journal of Clinical & Experimental Neuropsychology: Official Journal of the International Neuropsychological Society, 34*(4), 359-368. doi:10.1080/13803395.2011.645016

Luukkainen-Markkula, R., Tarkka, I., Pitkanen, K., Sivenius, J., & Hamalainen, H. (2011). Comparison of the Behavioural Inattention Test and the Catherine Bergego Scale in assessment of hemispatial neglect. *Neuropsychological Rehabilitation, 21*(1), 103-116. doi:10.1080/09602011.2010.531619

Malouin, F., Richards, C., Jackson, P., Lafleur, M., Durand, A., & Doyon, J. (2007). The Kinesthetic and Visual Imagery Questionnaire (KVIQ) for assessing motor imagery in persons with physical disabilities: A reliability and construct validity study. *Journal of Neurologic Physical Therapy, 31*(1), 20-29. doi:10.1097/NPT.0000260567.24122.64

Maruta, J., Suh, M., Niogi, S., Mukherjee, P., & Ghajar, J. (2010). Visual tracking synchronization as a metric for concussion screening. *The Journal of Head Trauma Rehabilitation, 25*(4), 293-305. doi:10.1097/HTR.0b013e3181e67936

Marvin, K. (2012). Trail Making Test (TMT). Retrieved from <https://www.strokengine.ca/en/assess/tmt/>

Matthey, S., Donnelly, S., & Hextell, D. (1993). The clinical usefulness of the Rivermead Perceptual Assessment Battery: Statistical considerations. *The British Journal of Occupational Therapy, 56*(10), 365-370. doi:10.1177/030802269305601003

Mattingley, J., Berberovic, N., Corben, L., Slavin, M., Nicholls, M., & Bradshaw, J. (2004). The greyscales task: A perceptual measure of attentional bias following unilateral hemispheric damage. *Neuropsychologia, 42*(3), 387-394. doi:10.1016/j.neuropsychologia.2003.07.007

Maxton, C., Dineen, R., Padamsey, R., & Munshi, S. (2013). Don't neglect 'neglect'- an update on post stroke neglect. *International Journal of Clinical Practice, 67*(4), 369-378. doi:10.1111/ijcp.12058

Mazer, B., Sofer, S., Korner-Bitensky, N., & Gelinas, I. (2001). Use of the UFOV to evaluate and retrain visual attention skills in clients with stroke: A pilot study. *American Journal of Occupational Therapy, 55*(5), 552-557. doi:10.5014/ajot.55.5.552

McDermott, A. (2012). Catherine Bergego Scale (CBS). Retrieved from <https://www.strokengine.ca/en/assess/cbs/>

Nijboer, T., Ten Brink, A., Kouwenhoven, M., & Visser-Meily, J. (2014). Functional assessment of region-specific neglect: Are there differential behavioural consequences of peripersonal versus extrapersonal neglect? *Behavioural Neurology, 2014*, 526407. doi:10.1155/2014/526407

Ogourtsova, T., Souza Silva, W., Archambault, P., & Lamontagne, A. (2017). Virtual reality treatment and assessments for post-stroke unilateral spatial neglect: A systematic literature review. *Neuropsychological Rehabilitation, 27*(3), 409-454. doi:10.1080/09602011.2015.1113187

Ota, H., Fujii, T., Suzuki, K., Fukatsu, R., & Yamadori, A. (2001). Dissociation of body-centered and stimulus-centered representations in unilateral neglect. *Neurology, 57*(11), 2064-2069. doi:10.1212/WNL.57.11.2064

Piscicelli, C., Nadeau, S., Barra, J., & Pérennou, D. (2015). Assessing the visual vertical: How many trials are required? *BMC Neurology, 15*(1), 1-5. doi:10.1186/s12883-015-0462-6

Politzer, T., Berryman, A., Rasavage, K., Snell, L., Weintraub, A., & Gerber, D. (2017). The Craig Hospital Eye Evaluation Rating Scale (CHEERS). *Pm & R, 9*(5), 477-482. doi:10.1016/j.pmrj.2016.08.032

Potter, J., Deighton, T., Patel, M., Fairhurst, M., Guest, R., & Donnelly, N. (2000). Computer recording of standard tests of visual neglect in stroke patients. *Clinical Rehabilitation, 14*(4), 441-446. doi:10.1191/0269215500cr344oa

Punt, T., Kitadono, K., Hulleman, J., Humphreys, G., Riddoch, M., Punt, T., . . . Riddoch, M. (2008). From both sides now: Crossover effects influence navigation in patients with unilateral neglect. *Journal of Neurology, Neurosurgery & Psychiatry, 79*(4), 464-466. doi:10.1136/jnnp.2007.139832

Razemba, F., Jacobs, L., & Franzsen, D. (2017). Convergent validity of the Occupational Therapy Adult Perceptual Screening Test (OT-APST) with two other cognitive-perceptual tools in a South African context. *South African Journal of Occupational Therapy, 47*(2), 3-10. doi:10.17159/2310-3833/2017/v47n2a2

Rorden, C., Hjaltason, H., Fillmore, P., Fridriksson, J., Kjartansson, O., Magnusdottir, S., & Karnath, H. (2012). Allocentric neglect strongly associated with egocentric neglect. *Neuropsychologia, 50*(6), 1151-1157. doi:10.1016/j.neuropsychologia.2012.03.031

Rorden, C., & Karnath, H.-O. (2010). A simple measure of neglect severity. *Neuropsychologia, 48*(9), 2758-2763. doi:10.1016/j.neuropsychologia.2010.04.018

Saviola, D., De Tanti, A., Conforti, J., Posteraro, L., Manfredini, A., Bagattini, C., & Basagni, B. (2018). Safe return to driving following severe acquired brain injury: Role of a short neuropsychological assessment. *European Journal of Physical & Rehabilitation Medicine., 54*(5), 717-723. doi:10.23736/S1973-9087.17.04905-X

Su, C., Chien, T., Cheng, K., & Lin, Y. (1995). Performance of older adults with and without cerebrovascular accident on the test of visual-perceptual skills. *American Journal of Occupational Therapy, 49*(6), 491-499. doi:10.5014/ajot.49.6.491

Su, C., Chang, J., Chen, H., Su, C., Chien, T., & Huang, M. (2000). Perceptual differences between stroke patients with cerebral infarction and intracerebral hemorrhage. *Archives of Physical Medicine & Rehabilitation, 81*(6), 706-714. doi:10.1016/S0003-9993(00)90097-2

Ten Brink, A., Visser-Meily, J., & Nijboer, T. (2018). Dynamic assessment of visual neglect: The mobility assessment course as a diagnostic tool. *Journal of Clinical and Experimental Neuropsychology, 40*(2), 161-172. doi:10.1080/13803395.2017.1324562

Ten Brink, A., Visser-Meily, J., & Nijboer, T. (2018). What does it take to search organized? The cognitive correlates of search organization during cancellation after stroke. *Journal of the International Neuropsychological Society, 24*(5), 424-436. doi:10.1017/S1355617717001254

Tippett, W., Alexander, L., Rizkalla, M., Sergio, L., & Black, S. (2013). True functional ability of chronic stroke patients. *Journal of Neuroengineering & Rehabilitation, 10*, 20. doi:10.1186/1743-0003-10-20

Tippett, W., & Sergio, L. (2006). Visuomotor integration is impaired in early stage Alzheimer's disease. *Brain Research, 1102*(1), 92-102. doi:10.1016/j.brainres.2006.04.049

Titus, M., Gall, N., Yerxa, E., Roberson, T., & Mack, W. (1991). Correlation of perceptual performance and activities of daily living in stroke patients. *American Journal of Occupational Therapy, 45*(5), 410-418. doi:10.514/ajot.45.5.410

Toglia, J., & Cermak, S. (2009). Dynamic assessment and prediction of learning potential in clients with unilateral neglect. *American Journal of Occupational Therapy, 63*(5), 569-579. doi:10.5014/ajot.63.5.569

Tsirlin, I., Dupierrix, E., Chokron, S., Coquillart, S., & Ohlmann, T. (2009). Uses of virtual reality for diagnosis, rehabilitation and study of unilateral spatial neglect: Review and analysis. *Cyberpsychology & Behavior, 12*(2), 175-181. doi:10.1089/cpb.2008.0208

Van der Stigchel, S., & Nijboer, T. (2018). Temporal order judgements as a sensitive measure of the spatial bias in patients with visuospatial neglect. *Journal of Neuropsychology, 12*(3), 427-441. doi:10.1111/jnp.12118

Van Deusen, J. (1988). Unilateral neglect: Suggestions for research by occupational therapists. *American Journal of Occupational Therapy, 42*(7), 441-448. doi:10.5014/ajot.42.7.441

Wang, Q., Sonoda, S., Hanamura, M., Okazaki, H., & Saitoh, E. (2005). Line bisection and rebisection: The crossover effect of space location. *Neurorehabilitation & Neural Repair, 19*(2), 84-92. doi:10.1177/1545968305274661

Warren, M. (1990). Identification of visual scanning deficits in adults after cerebrovascular accident. *American Journal of Occupational Therapy, 44*(5), 391-399. doi:10.5014/ajot.44.5.391

Weightman, M., Radomski, M., Mashima, P., & Roth, C. (2014). *Mild traumatic brain injury rehabilitation toolkit*. Borden Institute.

Weintraub, S. (1985). Mental state assessment of young and elderly adults in behavioral neurology. *Principles of Behavioral Neurology*, 71-123.

Wetzel, P., Lindblad, A., Raizada, H., James, N., Mulatya, C., Kannan, M., . . . Weaver, L. (2018). Eye tracking results in postconcussive syndrome versus normative participants. *Investigative Ophthalmology & Visual Science, 59*(10), 4011-4019. doi:10.1167/iovs.18-23815

Whitehouse, C., Green, J., Giles, S., Rahman, R., Coolican, J., & Eskes, G. (2019). Development of the Halifax Visual Scanning Test: A new measure of visual-spatial neglect for personal, peripersonal, and extrapersonal space. *Journal of the International Neuropsychological Society*, 1-11. doi:10.1017/S135561771900002X

Whitney, S., & Sparto, P. (2019). Eye movements, dizziness, and mild traumatic brain injury (mTBI): A topical review of emerging evidence and screening measures. *Journal of Neurologic Physical Therapy, 43*, S31-S36. doi:10.1097/NPT.0000000000000272

Yaretzky, A., Raviv, S., Netz, Y., & Jacob, T. (1995). Primary visual memory of stroke patients. *Disability & Rehabilitation, 17*(6), 293-297. doi:10.3109/09638289509166649

Zaninotto, A., Vicentini, J., Solla, D., Silva, T., Guirado, V., Feltrin, F., . . . Paiva, W. (2017). Visuospatial memory improvement in patients with diffuse axonal injury (DAI): A 1-year follow-up study. *Acta Neuropsychiatrica, 29*(1), 35-42. doi:10.1017/neu.2016.29

Zeltzer, L., & Poulin, V. (2012). Color Trails Test (CTT). Retrieved from <https://www.strokengine.ca/en/assess/ctt/>

Zeltzer, L. (2008a). Motor-Free Visual Perception Test (MVPT). Retrieved from <https://www.strokengine.ca/en/assess/mvpt/>

Zeltzer, L. (2008b). Ontario Society of Occupational Therapists (OSOT) Perceptual Evaluation. Retrieved from <https://www.strokengine.ca/en/assess/osot/>

Zeltzer, L., & Menon, A. (2008a). Clock Drawing Test (CDT). Retrieved from <https://www.strokengine.ca/en/assess/cdt/>

Zeltzer, L., & Menon, A. (2008b). Draw-A-Man test. Retrieved from <https://www.strokengine.ca/en/assess/damt/>

Zeltzer, L., & Menon, A. (2008c). Line Bisection test. Retrieved from <https://www.strokengine.ca/en/assess/lbt/>

Zeltzer, L., & Menon, A. (2010). Albert's test. Retrieved from <https://www.strokengine.ca/en/assess/at/>

Zoltan, B. (2007). *Vision, perception, and cognition: A manual for the evaluation and treatment of the adult with acquired brain injury, fourth edition (4^th^ ed.)*. Thorofare, NJ: Slack Incorporated.
