# Supplementary material for: Vision Evaluation Tools for Adults With Acquired Brain Injury: A Scoping Review
Source: Can J Occup Ther. 2021 Oct 18;88(4):340–51. doi: 10.1177/00084174211042955 (PMC8640270; doi:10.1177/00084174211042955)
Supplement: sj-docx-4-cjo-10.1177_00084174211042955 - Supplemental material for Vision Evaluation Tools for Adults With Acquired Brain Injury: A Scoping Review [file sj-docx-4-cjo-10.1177_00084174211042955.docx]

Appendix D

Psychometric properties of the tools

| **Name of the evaluation tool (s)** | **Sensitivity, Specificity & Predictive Value** | **Reliability** | **Validity** | **Other Psychometric Properties, Comments & Summary** |
| --- | --- | --- | --- | --- |
| Adult Visual-Perceptual Assessment | N/A | **Test-retest reliability**:  r = 0.17-0.77 (poor to excellent) (Titus, Gall, Yerxa, Roberson, & Mack, 1991) | N/A | **Other Psychometrics:** No statistically significant differences between the subjects with right or left lesions (Titus et al., 1991).  **Comments:** Due to poor test-retest reliability on certain subtests, authors conclude that this test should not be used to assess change (Titus et al., 1991). The Adult Visual-Perceptual Assessment showed consistent correlation with ADLs, therefore occupational therapists might consider using it to identify perceptual performance deficits that might affect performance on ADLs (Titus et al., 1991). |
| Albert's test | **Predictive Value:** 56.8% of individuals identified with visual neglect were true cases of neglect **(true positive)** (Fullerton, McSherry, & Stout, 1986). Approximately 4.3% of individuals without neglect were also screened negative **(true negative)** (Fullerton et al., 1986). | **Test-retest reliability:** r = 0.79 (excellent) (Sea & Henderson, 1994). | **Construct Convergent validity:** Excellent correlations (r = 0.85) with the Line Bisection Test (Agrell, Dehlin, & Dahlgren, 1997), adequate correlation with the Star Cancellation Test (r = 0.63) (Agrell et al., 1997), excellent correlation between the Wundt-Jastrow Area Illusion test and Albert’s test (r = 0.64) (Massironi, Antonucci, Pizzamiglio, Vitale, & Zoccolotti, 1988), and excellent correlation between the Catherine Bergego Scale and Albert’s test (r = 0.73) (Azouvi et al., 1996). Adequate (r = 0.31–0.59) to excellent (r ≥ 0.60) correlations with Single Letter Cancellation Test (ranging from r = 0.36 to r = 0.69) (Zoccolotti et al., 1989). Significant difference was found on the number of omissions in cancellation between patients with neglect (27.7 omissions) and those without neglect as well as age-matched controls (34.4-35.6 omissions) (Potter et al., 2000). **Concurrent validity:** r = -0.95, p ≤ 0.01 (excellent) with the CBS eating item (Luukkainen-Markkula, Tarkka, Pitkanen, Sivenius, & Hamalainen, 2011). | **Comments:** The Albert’s test is not suitable for use as a comprehensive screening tool for visual perception as it measures only left neglect, which is only one of multiple construct areas of visual perception (Cooke, McKenna, & Fleming, 2005). |
| Apples test | **Sensitivity:** 100% (Bickerton, Samson, Williamson, & Humphreys, 2011).  **Specificity:** 59% (Bickerton et al., 2011). | **Test-retest reliability:** 88% concordance rate on a classification of patients having/not having egocentric neglect and 94% concordance rate on classifying patients as having/not having allocentric neglect (Bickerton et al., 2011). **Interrater reliability:** κ = 0.13 (poor agreement) between the Apples test and the Bells test (Basagni et al., 2017). Agreement between the Apples test and the Bells test was higher in the acute sample with κ= 0.17 (poor) compared to the post-acute sample with κ = 0.09 (poor) (Basagni et al., 2017). k = 0.87, p = 0.001 (substantial agreement) between Star Cancellations and Apples test on the diagnosis of a page-based asymmetry (Munoz & Bangdiwala, 1997). | N/A | **Other Psychometrics:** Likelihood Ratios (calculated by the reviewers): *Positive LR:* 2.439. *Negative LR:* 0.  **Comments:** Using preliminary results, the Apples test is preferred when comparing to the Bells test to assess peripersonal neglect (Basagni et al., 2017). |
| Ayres' Figure-Ground test | N/A | **Interrater reliability:** r = 0.87 (excellent) (Baum, 1981). | N/A | **Comments:** The test is a subtest of the Southern California Sensory Integration tests (Zoltan, 2007). |
| Ayres' Space Visualization test | N/A | N/A | Validity is in doubt: When the test was administered to both R & L hemiplegic adults, the L hemiplegics did better as a group than the R hemiplegics, although the difference was not significant. Because it is generally thought that L hemiplegics do significantly poorer on spatial relations test, the validity of this test with adults is in some doubt (Bouska, Kauffman, & Marcus, 1990). | N/A |
| BTT | **Sensitivity:** 66.7% (Bailey, Riddoch, & Crome, 2000). | N/A | **Convergent validity:** r = -0.66 (excellent) with the Line Bisection Test (Zeltzer & Menon, 2008c). It is negative because a high score on the Line Bisection Test indicates the presence of unilateral spatial neglect whereas a high score on the BTT indicates normal performance (Zeltzer & Menon, 2008c). | N/A |
| BEN | **Sensitivity:** 85.9% for the whole battery (Azouvi et al., 2002). 19.9% to 50.5% for individual paper-and-pencil tests, with the Bells test (50.5%) and the reading test (46.8%) being the most sensitive measures (Azouvi et al., 2002). | **Internal consistency**: < 0.50 (poor inter-item correlation) for patients with L hemisphere stroke (Azouvi et al., 2006; Azouvi et al., 2002). 0.1 to 0.78 (poor to adequate inter-item correlation) for patients with R hemisphere stroke (Azouvi et al., 2006; Azouvi et al., 2002). | r=0.53-0.77 (adequate to excellent) for all pencil-and-paper tests with the CBS, except the Line Bisection subtest with r = 0.16-0.49 (poor) (Azouvi et al., 2006; Azouvi et al., 2002). | **Comments:** Line Bisection of short lines should not be recommended as a screening test for neglect (Azouvi et al., 2006; Azouvi et al., 2002). |
| Battery (Beis et al., 2004) | **Sensitivity:** On individual tests, personal neglect was found in 12.8% of the patients, and extra-personal neglect was found in 3.8-13.2% of the patients (Beis et al., 2004). When the whole battery was taken together, 43.5% demonstrated neglect on at least one measure, suggesting that the whole battery is more sensitive than any single test alone (Beis et al., 2004). | N/A | N/A | N/A |
| Battery (Saviola et al., 2018) | N/A | N/A | **Construct validity:** Both “pencil and paper” and computerized tests in the cognitive domains of attentive functions and those involving performance with visual-spatial material are significantly correlated with the driving test outcome, although is insufficient evidence of the relative value of off-road compared to direct on-road tests (Saviola et al., 2018). | **Other Psychometrics:** Spatial span and visuo-spatial supra-span appear to be predictive of the capacity to resume driving (Saviola et al., 2018).  **Comments:** Often used to assess driving. On-road testing is considered the gold standard for assessing driving ability, but since cognitive-behavioral disorders are the main causes of loss of driving ability and pose a risk for inappropriate road behavior, some authors agree a preliminary series of tests prior to on-road testing may be useful to exclude subjects with a high probability of failing the driving test (Saviola et al., 2018). |
| Battery (Rorden et al., 2012) | N/A | This battery was found to be a relatively reliable measure of allocentric neglect (Rorden et al., 2012). | For the validity of defect detection tests for allocentric neglect, predictive value of multiple tests of allocentric neglect were compared: When tested on 33 participants: their allocentric neglect score was strongly correlated with their egocentric neglect score, with a PCC of 0.799 (t(31) = 7.40, p < 0.00001) (Rorden et al., 2012). | **Other Psychometrics:** The authors have administered the defect detection task slightly differently from the conventional method (Rorden et al., 2012). In discussion, state that the defect detection task (Ota, Fujii, Suzuki, Fukatsu, & Yamadori, 2001) and the Apples test (Bickerton et al., 2011) do not provide accurate reliable measures of possible allocentric neglect when severe egocentric neglect is present (Rorden et al., 2012).  **Comments:** Authors discuss that when severe egocentric neglect is present, the defect defection task and the Apples test do not provide accurate reliable measures of possible allocentric neglect (Rorden et al., 2012). |
| Battery (Azouvi et al., 1996) | **Sensitivity:** The Bells test, Reading task, and Line Cancellation tasks were most sensitive, demonstrating neglect in 42-49% patients (Azouvi et al., 1996). | N/A | N/A | N/A |
| Battery (Barco, Wallendorf, Snellgrove, Ott, & Carr, 2014) | **Predictive Value:** A combination of Trail Making Test Part A and the SMT best predicted the probability of road test: **true positive rate** = 0.40, the **false positive rate** = 0.07 (based on cross-validation predictions with a cut point of 0.70 probability of failure) (Barco et al., 2014). | N/A | N/A | **Other Psychometrics:** Precision = 5.77, accuracy = 5.74, and **positive LR** 16.0 (95% CI [1.7, 21.1]) (based on cross-validation predictions with a cut point of 0.70 probability of failure) (Barco et al., 2014).  **Comments:** Often used in return to driving assessment. The Trail Making Test Part A and SMT combined were the best predictors of driving performance; may be useful for clinicians in determining whether a client should receive a comprehensive driving evaluation (Barco et al., 2014). |
| Battery (Akinwuntan et al., 2006) | **Sensitivity:** 79.4% (Akinwuntan et al., 2006).  **Specificity:** 94.1% (Akinwuntan et al., 2006).  **Predictive Value: PPV:** 93.1% (Akinwuntan et al., 2006). **NPV (calculated by the reviewers):** 82.1%. | N/A | N/A | **Other Psychometrics:** Likelihood ratios (calculated by the reviewers): *Positive LR:* 13.5. *Negative LR:* 0.219.  **Comments:** Often used in return to driving assessment. A combination of visual neglect, Figure of Rey, and On-road test constituted the best model to predict the 3-class group decision and accounted for 73% of the variance (Akinwuntan et al., 2006). |
| Battery (Bailey et al., 2000) | **Sensitivity:** Star Cancellation: 76.4%; Line Bisection: 76.4%; Baking Tray Task: 66.7%; Copy-a-Daisy: 57.5% (low sensitivity) (Bailey et al., 2000). | N/A | Because of the time needed to administer the full BIT, a shorter version has been validated (Bailey et al., 2000). Draw-a-Clock was found to have questionable validity in the assessment of representational neglect (Bailey et al., 2000). | **Comments:** Although it is restricted to testing neglect in the visual-spatial modality, the modified BIT battery may be more appropriate than the original BIT for use with elderly patients (Bailey et al., 2000). However, more research is required to assess the validity and sensitivity of the Exploratory Motor (EM) task for directional hypokinesia (Bailey et al., 2000). |
| BIT & BITC | **Sensitivity:** 19% to 50.5% (Maxton, Dineen, Padamsey, & Munshi, 2013). 93% for BITC (Halligan, Wilson, & Cockburn, 1990). 38% (Article Reading) to 100% (Star Cancellation and Coin Sorting) for R brain damaged patients (Figueiredo, 2011). 0% (Representational Drawing) to 100% (Letter Cancellation, Star Cancellation, Figure Copying, Telling Time, and Map Navigation) for L brain damaged patients (Figueiredo, 2011).  **Specificity:** 100% for BITC (Halligan et al., 1990). 64% (Star Cancellation, Representational Drawing, Article Reading) to 100% (Coin Sorting) for R brain damaged patients (Figueiredo, 2011). 77% (Star Cancellation) to 100% (Telling Time) for L brain damaged patients (Figueiredo, 2011).  **Predictive Value: PPV:** 74% and **NPV:** 94.5% for the BITC (Halligan et al., 1990). | **Interrater reliability**: r = 0.99-1.00 (Cermak & Hausser, 1989; Figueiredo, 2011; Van Deusen, 1988; Zoltan, 2007). **Parallel form reliability** was established between two test versions at 0.91 (p < 0.001) (Cermak & Hausser, 1989; Figueiredo, 2011). **Test-retest reliability**: r = 0.83-0.99 (excellent) (Cermak & Hausser, 1989; Figueiredo, 2011; Van Deusen, 1988; Zoltan, 2007) for the BIT. r = 0.89 (excellent) for the BITC and r = 0.97 (excellent) for the BITB (Figueiredo, 2011). | **Convergent validity:** r = -0.65 (excellent) between the BIT and the Occupational Therapist Checklist (Figueiredo, 2011). r = 0.55 (adequate) between the BIT and the Rivermead Activities of Daily Living Assessment (Figueiredo, 2011). r = 0.77 (excellent) between the BIT Behavioral subtest and the checklist of ADLs (Hartman-Maeir & Katz, 1995). r = 0.64 (excellent) between the BIT with the Barthel Index (Cassidy, Bruce, Lewis, & Gray, 1994). **Known groups validity:** Individuals with visual neglect performed significantly worse on the BITC as compared to healthy ones (p < 0.001) (Halligan, Cockburn, & Wilson, 1991). **Predictive validity:** Linear regression shows that BIT is an excellent predictor of poor functional outcomes, accounting for 73%, 64% and 61% of the total variance of the Frenchay Activities Index at 3, 6 and 12 months respectively (Figueiredo, 2011). | **Other Psychometrics:** Likelihood ratios (calculated by the reviewers): *Positive LR:* 15.3. *Negative LR:* 0.  **Comments:** The modified BIT battery may be more appropriate than the original BIT for use with elderly patients, although it is still restricted to testing neglect in the visuo-spatial modality (Bailey et al., 2000). The test appears to be useful to clinicians because it is relatively simple to administer, is standardized, and is intended to identify problems that patients will demonstrate in daily life (Cermak & Hausser, 1989). |
| Bells test | **Sensitivity:** 37.5%-49% in demonstrating neglect in stroke patients (Azouvi et al., 1996; Basagni et al., 2017). | N/A | **Predictive validity**: r = 0.622, p = 0.04 (moderate) between the Bells test and time taken searching on the right with Tobii eye-tracking glasses (Kortman & Nicholls, 2016). The total number of omission at the Bells test was significant (but not the asymmetry score) in predicting whether stroke patients would pass or fail their on-road driving exam (Saviola et al., 2018). | N/A |
| Bender Visual Motor Gestalt Test | N/A | **Test-retest reliability:** r = 0.89 (excellent) (Titus et al., 1991). | N/A | **Other Psychometrics:** There were no statistically significant differences between the subjects with right or left lesions (Titus et al., 1991).  **Comments:** The Bender Visual Motor Gestalt Test was one of the most discriminating in differentiating this sample of stroke patients from normative samples on perceptual performance (Titus et al., 1991). |
| BLO | **Sensitivity:** Scores on the BLO were significantly different between stroke patients and control group (F1,12 = 12.24, p < 0.005) (Tippett, Alexander, Rizkalla, Sergio, & Black, 2013). There were no within group effects observed (p > 0.05) (Tippett et al., 2013).  **Specificity:** There was no significant difference in variability between the groups for the BLO (Tippett et al., 2013). | N/A | N/A | **Comments:** Research has shown that the right parietal region is active during BLO task performance using functional neuroimaging (Tippett et al., 2013). The BLO has been shown to correlate with right parietal damage and is one of few noted “pure” visuospatial measures (Tippett et al., 2013). |
| Block Design and Object Assembly subtest of the WAIS-R | N/A | **Internal consistency** for Block Design subtest: r = 0.87 (excellent) and for the Object Assembly subtest: r = 0.68 (adequate) (Titus et al., 1991). | N/A | **Other Psychometrics:** There were no statistically significant differences between the subjects with right or left lesions (Titus et al., 1991).  **Comments:** The Block Design subtest of the WAIS-R. The Adult Visual-Perceptual Assessment showed consistent correlation with ADLs, therefore occupational therapists might consider using it in order to identify perceptual performance deficits that might affect ADL performance (Titus et al., 1991). |
| BIVSS | **Sensitivity:** 82.2% (correct in predicting TBI) (Laukkanen, Scheiman, & Hayes, 2017).  **Specificity:** 90.4% (correct in predicting non-TBI status) (Laukkanen et al., 2017). | **Person reliability:** 0.91 with 3.18 separation  **Item reliability:** 0.97 with 5.72 separation based on Rasch analysis (excellent) (Laukkanen et al., 2017). | N/A | **Other Psychometrics:** Likelihood ratios (calculated by the reviewers): *Positive LR:* 8.56. *Negative LR:* 0.197.  **Comments:** Sensitivity and specificity determined based on Rasch scale with a 0.5 cutoff criteria (Laukkanen et al., 2017). Total raw score equivalent cut-off for the specified sensitivity and specificity: ≥32 on the revised 25-item scale (Laukkanen et al., 2017). |
| CBS | **Sensitivity:** The behavioural assessment of CBS was more sensitive to the presence of UN than any single paper-and-pencil test with the sensitivity ranges from 32.81% to 68.25% (Azouvi et al., 2006; Azouvi et al., 2002). This was not statistically different from the sensitivity of the whole paper-and-pencil battery (χ2 test = 2.7, df = 1, p > 0.1) (Azouvi et al., 2002). The highest incidence of neglect in conventional tests comprised of the Bells test, a figure copying task, clock drawing, line bisection task, overlapping figures test, reading task and a writing task was 50%, whereas neglect was seen on at least 1 of the 10 CBS items in 76% of patients (Azouvi et al., 2006). | CBS is reliable in subacute and chronic stroke patients (Azouvi et al., 2003) and is used as a reliable measure of neglect in everyday life (Azouvi et al., 1996). **Internal consistency**: Cronbach’s alpha = 0.48–0.94, p < 0.05 to p < 0.01 (adequate to excellent) (Azouvi et al., 1996; Azouvi et al., 2003; Bergego et al., 1995; Luukkainen-Markkula et al., 2011; Nijboer, Ten Brink, Kouwenhoven, & Visser-Meily, 2014). **Interrater reliability**: kappa coefficient range = 0.59–0.99 (adequate to excellent) (Bergego et al., 1995). **Correct item-total correlation range**: 0.63-0.90 (adequate to excellent) (Azouvi et al., 2003; Nijboer et al., 2014). | CBS valid in subacute and chronic people with a stroke (Azouvi et al., 2003). **Concurrent validity:** r range = 0.41–0.77, p < 0.0001 to p < 0.01 (adequate to excellent) for correlations reported between the CBS and the Bells test, the Ogden's scene drawing task, the writing task, the reading task, the Albert's Test and the flower drawing task (Azouvi et al., 1996; Azouvi et al., 2006; Azouvi et al., 2003; Azouvi et al., 2002; Bergego et al., 1995). There was no significant correlation with the line bisection test (Azouvi et al., 1996; Azouvi et al., 2006; Azouvi et al., 2003; Azouvi et al., 2002). **Construct validity:**  A single underlying factor explained 65.8% of total variance in CBS (Azouvi et al., 2003). All 10 items obtained a high loading on this factor (range = 0.77–0.84) (Azouvi et al., 2003). **Known Group validity:** Significant difference in total CBS scores between the two patients with behavioural neglect on conventional tests and patients with no neglect (p < 0.0001) (Azouvi et al., 1996). Patients with right hemisphere stroke and with visual field deficits demonstrated significantly more severe behavioral neglect (i.e. higher CBS total score) than patients with right hemisphere stroke and with intact visual fields (p = 0.03) (Luukkainen-Markkula et al., 2011). **Convergent/Discriminant validity** : r = -0.63, p < 0.0001(excellent) correlation to the aspects of daily functioning related to neglect measured by the Barthel Index (Azouvi et al., 2003).  r = -0.48, p < 0.01 (adequate) correlation between the CBS score and the FIM (Azouvi et al., 2006). | **Other Psychometrics:** Floor/Ceiling effect: adequate (Philippe Azouvi et al., 2003).  Responsiveness: CBS was found sensitive to clinical change, and useful to monitor patients’ improvement after rehabilitation (Azouvi et al., 2006; Samuel et al., 2000). |
| Checklist for Vision Problems Post Stroke | **Sensitivity:** 69% (ability of the tool in identifying ocular conditions and vision defects identified by the orthoptist) (Jolly, Macfarlane, & Heard, 2013). | N/A | N/A | **Comments:** Use of this tool is limited, as it has not been used by non–eye care practitioners for whom it was developed (Jolly et al., 2013). Non-orthoptic health professionals identified only 17% of the conditions (compared to 69% identified by the tool), but the tool was less accurate than unaided non-orthoptic health professionals for pupils, field loss, neglect, and nystagmus (Jolly et al., 2013). Conclusion: tool has a better capacity to reveal ocular conditions and VD than input from unaided non-orthoptic healthcare practitioners (Jolly et al., 2013). |
| CDT | **Sensitivity:** 27.8% (Azouvi et al., 2002). When compared to a whole battery, 13.2% patients were found to have signs of right neglect based on the CDT, comparatively to 43.5% on the battery (Bailey et al., 2000).  **Specificity:** when used as a subtest of the SNAP, along with drawing a daisy: 99% (Leibovitch, Vasquez, Ebert, Beresford, & Black, 2012). | **Test-retest reliability:** r = 0.87-0.94 (excellent) for 2-day test-retest (Manos & Wu, 1994); r = 0.70 (adequate) for 4 days test-retest (Tuokko, Hadjistavropoulos, Miller, & Beattie, 1992) and r = 0.76-0.78 (excellent) for 3-6 months test-retest (Mendez, Ala, & Underwood, 1992). **Interrater reliability:** r = 0.63-0.97 (adequate to excellent) (Zeltzer & Menon, 2008a). | Correlation of the CDT with the MMSE and the FIM: r = 0.51 to 0.59 (adequate) (Adunsky, Fleissig, Levenkrohn, Arad, & Noy, 2002). The CDT has questionable validity in the assessment of representational neglect (Bailey et al., 2000). | **Comments:** Drawings are the second most frequently used tests for spatial neglect (Azouvi et al., 2002). Other versions: Verbal command free drawn clock or pre-drawn clock (simultaneously assess language, memory and executive function); Copy command (less reliance language and memory, more on visuospatial and perceptual skills); Clock reading test; Time-setting: “10 after 11” (Zeltzer & Menon, 2008a). |
| CTT | N/A | **Test-retest reliability**: r = 0.64 (adequate) for CTT1 and r = 0.79 (excellent) for CTT2 in healthy individuals (D'Elia, Satz, Uchiyama, & White, 1996). | **Construct Validity (Known groups validity):** Clients in the stroke group required significantly more time to complete the CTT1 and CCT2 than the healthy controls (p < 0.001) (Messinis, Malegiannaki, Christodoulou, Panagiotopoulos, & Papathanasopoulos, 2011). **Construct Validity (Convergent/discriminant validity):** r = 0.407-0.602 (adequate to excellent) for CTT1 and r = not significant to 0.629 for CTT2 (poor to excellent) with Useful Field of View (UFOV) processing speed subtests processing speed, divided attention and selective attention (Hartman-Maeir, Erez, Ratzon, Mattatia, & Weiss, 2008). **Construct Validity (Concurrent validity):** r = 0.91 (excellent) of CTT1 with TMT-A and r = 0.72 (excellent) of the CTT2 with TMT-B (Elkin-Frankston, Lebowitz, Kapust, Hollis, & O'Connor, 2007). **Criterion Validity (Predictive validity)**: Performance time < 60 seconds on the CTT1 was found to predict passing the on-road evaluation, whereas > 60 seconds was predictive of failing (Cohen’s d = 0,66-0.67, p < 0.05) (Elkin-Frankston et al., 2007; Hartman-Maeir et al., 2008). The CTT2 observes the same relationship but is not statistically significant (Elkin-Frankston et al., 2007). | **Other Psychometrics:** Responsiveness: The CTT detected change in both clients with reduced time to complete the CTT1 and CTT2 after mental imagery intervention (Liu, Chan, Lee, & Hui-Chan, 2004). |
| CbVM | N/A | N/A | N/A | **Other Psychometrics:** The effect size values (Cohen's D) for the CbVM ranged from 0.04-0.86, with 2 of the MT measures having large effect size value (range 0.76-0.86) (Tippett et al., 2013). The effect size results show that individuals in the post subacute phase of a stroke still display significant impairments on a number of the neuropsychological tasks (Tippett et al., 2013). **Inter-individual variability:** The average IT and MT coefficient of variation for the patient and control groups indicated significant differences in variability, with the patient group having greater coefficient of variation for IT (0.45 vs. 0.22) and MT (0.38 vs. 0.24) than controls (Tippett et al., 2013). **Intra-individual variance:** individuals with greater frontal regional damage demonstrated consistently higher variability on IT than MT, and vice-versa for individuals with greater parietal regional damage (Tippett et al., 2013). Moreover, an overall strong correlation was demonstrated between injury severity and the measure of variability (r = 0.69, p < 0.05) (Tippett et al., 2013).  **Comments:** Each of the 4 conditions involved 20 trials. This study concludes that analysis of neuropsychological tests requires consideration of the performance variability on tests; a lack of performance consistency was found in stroke patients (Tippett et al., 2013). This calls into question the accuracy of diagnosis of other measures without an examination of variability. It is therefore important to have an assessment tool that could assist in monitoring performance variability, such as the CbVM tool (Tippett et al., 2013). In addition, the use of this tool may also be able to shed light on whether performance variability in brain injured populations improves with time or how this may dissociate from general slowness. Fluctuations in performance may underlie some of the difficulties, such as fatigue, which are commonly reported by patients with stroke (Tippett et al., 2013). |
| VISSTA | **Sensitivity:** to distinguish between neglect and normal performance was tested by comparing the scores of the RHD USN+ and the control group. Results revealed significant disadvantage for the neglect group in all measures (Erez, Katz, Ring, & Soroker, 2009). | **Test-retest reliability:** r = 0.692-0.895 (adequate to excellent) (Erez et al., 2009). | Correlation between HR in visual search (in VISSTA) and the total score in the MWCT, a widely used standardised test for neglect, based on target cancellation (Weintraub, 2000): r = 0.5-0.6 for RHD groups (adequate to excellent correlation) (Erez et al., 2009). Analysis of the conjunction search performance revealed correlations of the HR with the MWCT scores in both RHD, LHD and controls:  r = 0.5-0.8 (adequate to excellent) (Erez et al., 2009). The findings of the present study indicate that the VISSTA, which employs computerised visual search tasks, is a valid tool for the assessment of disturbances in spatial attention arising from stroke (Erez et al., 2009). | **Comments:** In clinical practice, assessment of USN is usually performed using traditional paper-and-pencil tests (Robertson, 1993; Weintraub, 2000; Wilson, Cockburn, & Halligan, 1987) . Adding a computerized test for the assessment of visual-spatial inattention after stroke provides sensitive and useful information (Erez et al., 2009). It provides both HR and RT quantitative measures that can serve in the longitudinal monitoring of recovery and for the evaluation of the efficacy of rehabilitation efforts directed toward normalisation of lateralized inattention and neglect (Erez et al., 2009). |
| CHEERS | **Sensitivity:** Average of 95.45% for the total score (Politzer et al., 2017).  **Specificity:** Average of 55.55% for the total score (Politzer et al., 2017).  **Predictive Value:** **Average PPV:** 72.8% for the total score (Politzer et al., 2017). **Average NPV:** 92.85% for the total score (Politzer et al., 2017). | **Average intra-rater reliability:** 0.9855 for the total score (excellent) (Politzer et al., 2017). **Interrater reliability: Test:** 0.63 for the total score (adequate) and **Retest:** 0.69 for the total score (adequate) (Politzer et al., 2017). | N/A | **Other Psychometrics:** Likelihood ratios (calculated by the reviewers): *Positive LR:* 2.15. *Negative LR:* 0.082.  **Comments:** The psychometric properties are based on only 2 raters (Politzer et al., 2017). The CHEERS was designed with expert input from multiple specialists (neuro-optometry/ophthalmology, OT, etc.)(Politzer et al., 2017). Level of evidence: IV (Politzer et al., 2017). |
| Design Copy Test | N/A | The Spearman rank order correlations were statistically significant for both the **interrater** and **test-retest** comparisons for all tests (Warren, 1990). | Differences in performance between the hemiplegic and control groups were statistically significant for all tests, with the hemiplegic group achieving lower scores compared to controls (Warren, 1990). This indicates that the tests can identify visual deficits in post-stroke persons (Warren, 1990). | **Comments:** Hemiplegic subjects demonstrated deficits in visual scanning speed, identification of visual stimuli in the hemifield on the affected side, use of a systematic search pattern, and ability to accurately reproduce visual designs (Warren, 1990). A person with ABI may miss key elements in an aspect of the environment and as a result fail to make accurate spatial judgments (Warren, 1990). If this occurs during visual-perceptual tests, it may be interpreted as a deficit in figure-ground perception or visual memory, while the true deficit lies in the efficiency of visual scanning (Warren, 1990). This misinterpretation occurs because the current tests of visual perception focus on the evaluation of a higher-level skill and rarely contain subtests that measure basic oculomotor functions such as scanning (Warren, 1990). |
| DEM Test | N/A | **Internal consistency:** Significant correlations between all subtests (p < 0.001) except vertical time and ratio score (r = –0.05) (Garzia, Richman, Nicholson, & Gaines, 1990). **Test-retest reliability:**  Vertical time r = 0.89 (p < 0.001) (excellent), horizontal time r = 0.86 (p < 0.001) (excellent), ratio r = 0.57 (p < 0.01) (adequate) when performing the DEM Test on 2 occasions 1 week apart (Tassinari & DeLand, 2005). 2 other studies show poor test-retest reliability for vertical, horizontal, and ratio (Tassinari & DeLand, 2005). **Interrater reliability:** Horizontal time r = 0.91 (p < 0.001) (excellent), vertical time r = 0.81 (p < 0.001) (excellent), ratio r = 0.57 (p < 0.01) (adequate) (Garzia et al., 1990). | **Construct validity:** Vertical time r = –0.79; horizontal time r = –0.78; ratio = –0.55, p < 0.001 (adequate to excellent) when comparing the Wide Range Achievement Test with all DEM subtests (Garzia et al., 1990). | **Comments:** Even though normative data is only available up to age 13, the test can be used with adults with ABI because only limited additional improvement is expected in performance on this test with increased age. The adult with ABI should perform at least as well as the top level norms (Lyon, Goss, Horner, Downey, & Rainey, 2005). |
| DTVP-A | N/A | **Test-retest reliability**: Cronbach’s alpha = 0.91 to 0.94 (excellent) for the total scale (Brown, Mullins, & Stagnitti, 2008). | **Construct validity:** r = 0.00-0.42 (poor) between the DTVP-A and the OT-APST (Brown, Mapleston, & Nairn, 2011). **Predictive validity:** DTVP-A visual-motor search subscale was found to be the only negative independent predictor of FIM cognitive scale, accounting for 21% of variance (Brown, Mapleston, & Nairn, 2012) . | **Comments:** Although the DTVP-A is the most in-depth and time-consuming test to administer and interpret between the OT-APST and the Cognistat, it is the most appropriate tool to use to predict functional abilities (Brown et al., 2012). |
| DAT | N/A | N/A | The results on the DAT mimic results found on line bisection tests previously demonstrated in patients with neglect (Punt et al., 2008). | N/A |
| DLCT | N/A | **Test-retest reliability:** r = 0.62 (adequate) (Gordon et al., 1984). | Correlated with mean CT-scan damage: r = -0.35 (adequate correlation) (Egelko et al., 1988). | **Comments:** The finding suggests that the DLCT should be used as a screening tool rather than as a diagnostic tool for USN (Zeltzer, 2008b). This is known to be a more taxing measure of USN than the Single Letter Cancellation Test (Zeltzer, 2008b). It requires language skills to be sufficient to identify letters, and therefore may not be suitable for patients with receptive aphasia (Zeltzer, 2008b). |
| Draw-A-Man Test | N/A | **Test-retest reliability:** r = 0.50-0.62 (adequate) (Chen-Sea, 2000; Gordon et al., 1984)  **Interrater reliability:** r = 0.96-1.00 (excellent) (Chen-Sea, 2000). | **Construct (Convergent) validity:** Significant correlations of the Draw-A-Man Test with ADL performance measured by the Klein-Bell ADL Scale (Chen-Sea, 2000). **Construct (Known groups) validity:** The Draw-A-Man Test was able to discriminate patients with personal neglect from those without personal neglect (Chen-Sea, 2000). | **Other Psychometrics:** Responsiveness: The Draw-A-Man Test does not detect change in patients (Zeltzer & Menon, 2008b). |
| Eye Alignment Test | N/A | **Interrater reliability:** r = 0.92 (excellent) (Rainey, Schroeder, Goss, & Grosvenor, 1998). **Intrarater reliability:** No difference between the results of the various tests was “statistically significant” for repeatability (Antona et al., 2011). | **Criterion validity:** compared the modified Thorington test was compared with 3 other tests (von Graefe technique, Maddox rod test, and prism cover test) and concluded that due to the low level of agreement observed between these tests, interchangeability is not recommended in clinical practice (Antona et al., 2011). | **Other Psychometrics:** Not normalized on adults with mild TBI (Weightman, Radomski, Mashima, & Roth, 2014).  **Comments:** Test should only be performed once with the Maddox rod, it is not necessary to repeat the test (Weightman et al., 2014). |
| Eye movements recorded binocularly with video-oculography device | N/A | N/A | **Construct validity:** r = 0.60 and 0.54 (adequate to excellent) between variability measures for both the radial and tangential visual tracking errors and the overall mean reaction time in the ANT and r = −0.49 and −0.46 (adequate) with the orienting effect in the ANT (Maruta, Suh, Niogi, Mukherjee, & Ghajar, 2010). r = −0.48 to −0.60, (adequate to excellent) between the variability measure for the visual tracking errors with the total recall discriminability, recognition discriminability indices of CVLT-II (Maruta et al., 2010). | **Comments:** Performance variability during predictive visual tracking is a powerful indicator for decreased integrity in frontal white matter tracts vulnerable to mTBI as well as for altered cognitive functioning (Maruta et al., 2010). Gaze error variability was significantly correlated with the mean FA values of the right anterior corona radiata (ACR) and the left superior cerebellar peduncle (Maruta et al., 2010). Because the ACR and the genu are among the most frequently damaged white matter tracts in mTBI, the correlations imply that gaze error variability during visual tracking may provide a useful screening tool for mTBI (Maruta et al., 2010). Gaze error variability was also significantly correlated with attention and working memory measures in neurocognitive testing; thus, measurement of visual tracking performance is promising as a fast and practical screening tool for mTBI (Maruta et al., 2010). Visual tracking performance can be monitored precisely and continuously, allowing detection and objective quantification of subtle momentary lapses in attention over a matter of seconds, a significantly shorter time than required for administration of traditional neurocognitive testing (Maruta et al., 2010). Measurement of visual tracking performance is promising as a fast and practical screening tool for mTBI (Maruta et al., 2010). |
| Eye tracking assessments via an EyeLink 1000 remote eye tracking system | N/A | N/A | No difference between mTBI and normal participants in main sequence profiles was observed. On the circular task, intersaccadic interval duration was shorter in mTBI compared with normal subjects (horizontal:  Cohen’s D=0.65; vertical:  Cohen’s D=0.75).  For reading, absolute saccadic amplitudes (Cohen’s D=0.76) and average forward saccadic amplitudes were lower (Cohen’s D=0.61). Absolute fixation velocity was higher (Cohen’s D=1.02), and overall fixation durations (Cohen’s D=0.58), regression durations (Cohen’s D=0.49), and forward saccadic durations (Cohen’s D=0.54) were longer. mTBI participants had more fixations (Cohen’s D=0.54) and regressions per line (Cohen’s D=0.70) and read fewer lines (Cohen’s D=0.38) than normal subjects. On the horizontal ramp task, mTBI participants had lower weighted smooth pursuit gains (Cohen’s D=0.55).  On the horizontal step task, mTBI participants had shorter mean fixation times (Cohen’s D=0.55). These results suggest vulnerability of the smooth pursuit and saccadic systems in mTBI. Eye tracking shows promise as an objective, sensitive assessment of damage after mTBI (Wetzel et al., 2018). | **Comments:** It is concluded that eye tracking appears to be an objective and sensitive assessment of damage after mTBI(Wetzel et al., 2018). |
| Form H of Judgment of Line Orientation | N/A | **Internal consistency** r = 0.94 (excellent) (Titus et al., 1991). | N/A | **Other Psychometrics:** There were no statistically significant differences between the subjects with right or left lesions (Titus et al., 1991).  **Comments:** Among other tests, the Judgment of Line Orientation was one of the most discriminating in differentiating this sample of stroke patients from normative samples on perceptual performance (Titus et al., 1991). |
| Formboard Test | N/A | **Interrater reliability:** r = 1.0 (excellent) established on a sample of adult clients with head trauma (Baum, 1981). | To improve validity, rule out poor vision, visual field loss, poor color discrimination, and constructional apraxia as causes of poor performance (Zoltan, 2007). | N/A |
| GST | N/A | **Test-retest reliability:** r = 0.25-0.48 (poor) for individuals with vestibular disease and r = 0.62-0.79 (adequate to excellent) in a healthy population (Dunlap et al., 2018) | Correlation with the ABC Scale: r = 0.20-0.21 (poor correlation) and with the DHI: r = 0.18-0.22 (poor correlation). Correlation with the visual motor processing speed domain within ImPACT: r = 0.24 (poor correlation) (Dunlap et al., 2018) | **Comments:** The GST measures the maximum voluntary rotational head velocity that can be attained while accurately identifying a fixed optotype size. It is a functional measure of VOR and may be useful in determining readiness for discharge from vestibular physical therapy (Dunlap et al., 2018). Since the GST is a psychophysical test, visual motor processing speed is likely a component of the performance, and impairments on the test may indicate a need for vestibular rehabilitation (Dunlap et al., 2018). |
| GEMAT visual-memory test | N/A | **Internal consistency**: r = 0.86-0.89 (excellent) (Yaretzky, Raviv, Netz, & Jacob, 1995). | **Construct validity:** r = 0.33-0.44 (adequate) using 22 different performance tests such as a tachistoscope test for examining visual perception, the line labyrinth test for examining visual structuring ability and the concentration test Q1 (Yaretzky et al., 1995). **Known group validity:** Able to identify differences between patients and controls and between patients with high and low MMSE score (Yaretzky et al., 1995). Patients were slower (p < 0.01) and made more mistakes (p < 0.01) than controls (Yaretzky et al., 1995). Significant differences were indicated between patients with high (27-30) and low (24-26) score of MMSE on the GTT (Yaretzky et al., 1995). Patients who scored 24-26 (n = 9) were slower (x = 310, SD = 136) (p < 0.05) (Yaretzky et al., 1995) . | **Comments:** It is culture-free and suitable for patients unfamiliar with computerized technology (Yaretzky et al., 1995). It is also designed for patients with upper extremity motor disability (Yaretzky et al., 1995). The test variables are: (a) the number of correct GEMAT answers (GCA), and (b) the time required by the subject to complete the test (GTT) (Yaretzky et al., 1995). |
| Greyscales task | N/A | N/A | RH patients with no neglect and full visual fields still showed an abnormal bias on the Greyscales task, which suggests that the Greyscales task can measure attentional bias separately from clinical neglect and visual sensory loss (Mattingley et al., 2004). | **Other Psychometrics:** The test was shown to be a strong predictor of driving test performance in unilateral stroke patients with visual impairments, as scores on Greyscales task had a high correlation with visual performance during driving (r = 0.81) (Tant, Brouwer, Cornelissen, & Kooijman, 2002). |
| Gross Visual Skills | N/A | **Interrater reliability** of the overall Gross Visual Skills assessment: r = 0.83 (excellent) (Titus et al., 1991). | N/A | **Other Psychometrics:** There were no statistically significant differences between the subjects with R or L lesions (Titus et al., 1991). |
| HVST | N/A | N/A | **Construct validity:** Comparison of each subtests with 3 of the BIT subtests (Star Cancellation, Letter Cancellation and Line Crossing): r = 0.38-0.74 (adequate to excellent) except for the one correlation (between the HVST Wall subtest and BIT Letter cancellation): r = 0.33 (poor) (Whitehouse et al., 2019). | **Comments:** Performance is not affected by a motor deficit (Whitehouse et al., 2019). Subtests less related to conventional tasks from the BIT subtests may be identifying additional aspects of neglect not captured by the BIT (Whitehouse et al., 2019). |
| Haptic Visual Discrimination Test | N/A | **Test-retest reliability** for overall test: r = 0.93 (excellent) (Titus et al., 1991). | N/A | **Other Psychometrics:** Subjects with left hemispheric lesions performed better on the left-handed Haptic Visual Discrimination Test subtests, and those with right hemispheric lesions performed better on the right-handed Haptic Visual Discrimination Test subtests (Titus et al., 1991).  **Comments:** The Haptic Visual Discrimination Test was one of the most discriminating in differentiating this sample of stroke patients from normative samples on perceptual performance (Titus et al., 1991). |
| HVOT | N/A | N/A | **Construct validity:** Correlations with 4 Cognistat subtests (Confrontation Naming, Construction, Memory and Similarities): r = 0.27-0.56 (poor to adequate); the WAIS-R Perceptual Organization subtest: r = 0.65 (excellent correlation) and Performance subtests: r = 0.43-0.63 (adequate to excellent correlation) (Greve, Lindberg, Bianchini, & Adams, 2000). It therefore appears that the HVOT is a valid measure of visual spatial ability (Greve et al., 2000). | **Other Psychometrics:** Predicting rehabilitation and functional outcome: HVOT scores did not correlate significantly with FIM scores when admission was controlled (r = 0.01-0.37; r squared = 0.00-0.14), and it did not correlate significantly with length of hospitalization (r = 0.093, r squared = 0.01) (Greve et al., 2000). This suggests that HVOT does not provide any additional information regarding rehabilitation and functional outcome (Greve et al., 2000). |
| ImPACT | **Sensitivity:** 81.9% (Schatz, Pardini, Lovell, Collins, & Podell, 2006).  **Specificity:** 89.4% (Schatz et al., 2006).  **Predictive Value:** **PPV:** 89.4% (Schatz et al., 2006) **NPV:** 81.9% (Schatz et al., 2006). | Reliability for each subscale: r = 0.65-0.86 (adequate to excellent) (Schatz, 2010). | ImPACT is a widely used computerized neurocognitive test and has been validated as an evaluative tool in sports-related concussion (Dunlap et al., 2018). A correlation of r = 0.24 (poor correlation) was found between horizontal GST and the visual motor processing speed domain within ImPACT (Dunlap et al., 2018). | **Other Psychometrics :** Likelihood rations : *Positive LR:* 7.73 (Schatz et al., 2006) *Negative LR:* 0.20 (Schatz et al., 2006). |
| KVIQ-20/ KVIQ-10 | N/A | **Test-retest reliability:** ICC = 0.72-0.90 (adequate to excellent) (Malouin et al., 2007). **Internal consistency:** The Cronbach α = 0.87-0.94 (excellent) (Malouin et al., 2007). Items-to-corrected items correlation ranges were 0.63-0.83 (KVIQ-20) and 0.62-0.77 (KVIQ-10), indicating that no item was problematic (Malouin et al., 2007). | **Construct validity:** The KVIQ demonstrated very good item-to-goal score consistency, which suggests that all items measured the same construct (Malouin et al., 2007). | **Comments:** Given the good psychometric properties of both versions, the authors suggest that the KVIQ-10 appears to be a good choice for assessing persons with physical disabilities because it can be administered in half the time (Malouin et al., 2007). |
| King-Devick test | N/A | **ICC** of 0.91(excellent) (Worts & Burkhart, 2019). | Valid measure of saccadic eye movements under a fixed test condition (Galetta et al., 2011; King, Brughelli, Hume, & Gissane, 2013) | **Other Psychometrics:** The King-Devick has a higher false-positive rate (36%) at detecting mTBI than the VOMS tool (2%) (Worts & Burkhart, 2019).  **Comments:** King-Devick test is a brief assessment and does not measure oculomotor functions such as pursuits, convergence, and accommodation, and therefore may be limited in its screening capability (Collins, Kontos, Reynolds, Murawski, & Fu, 2014). |
| Light Show Device Tests | N/A | The Spearman rank order correlations were statistically significant for both the **interrater** and **test-retest** comparisons for all tests (Warren, 1990). | Differences in performance between the hemiplegic and control groups were statistically significant for all tests, with the hemiplegic group achieving lower scores compared to controls (Warren, 1990).This indicates that the tests are valid to identify visual deficits in post-stroke persons (Warren, 1990) | N/A |
| Line bisection test | **Sensitivity:** 76.4% (Bailey et al., 2000). Longer lines (20 cm) were nearly twice as sensitive than shorter (5 cm) ones. Bisection of short lines was the less sensitive test in the battery and did not correlate with behavioural neglect (Azouvi et al., 2002). The VR test was more sensitive than pen and paper alone (Tsirlin, Dupierrix, Chokron, Coquillart, & Ohlmann, 2009). | **Test-retest reliability**: r = 0.64-0.97 (adequate to excellent) (Bailey, Riddoch, & Crome, 2004; Kinsella, Packer, Ng, Olver, & Stark, 1995; Schenkenberg, Bradford, & Ajax, 1980; Sea & Henderson, 1994), r = 0.60-0.68 (moderate) for left-placed lines (Downing, 1986; Van Deusen, 1988). | **Construct validity:** r = -0.40 (adequate) correlation of the Line Bisection Test with the Star Cancellation Test (Marsh & Kersel, 1993). It is negative as a high score on the Line Bisection Test indicates USN, whereas a score close to 0 on the Star Cancellation Test indicates the absence of USN (Marsh & Kersel, 1993). However, a study reported no significant correlation with the Star Cancellation Test (Schubert & Spatt, 2001) r = (-0.37)-(-0.59) (adequate) between the Line Bisection Test with CT-scan damage of temporal lobe, parietal lobe, and occipital lobe (Egelko et al., 1988). **Construct (convergent) validity:** r = -0.66 (excellent) with the BTT (Bailey et al., 2000). No significant correlation between line bisection with Letter Cancellation Test (Binder, Marshall, Lazar, Benjamin, & Mohr, 1992). r = 0.05 (poor) with the CDT (Ishiai, Sugishita, Ichikawa, Gono, & Watabiki, 1993). r = -0.21; p = 0.08 (no significant correlation) between Line bisection vs. TOJ test (Van der Stigchel & Nijboer, 2018). r = 0.85 (excellent) with Albert’s Test (Agrell et al., 1997). RPAB, BIT, Drawing and copying tests, Line Bisection tasks and Cancellation tests correlate well with one another and appear to measure a common underlying construct (ie spatial neglect) (Bohannon, 2003). **Known groups validity:** The line bisection test clearly differentiated the subjects with right-brain lesions, 90% of whom had unilateral neglect, from the other groups (Schenkenberg et al., 1980; Van Deusen, 1988) **Predictive validity**: Can predict the severity of UN in daily activities (Wang, Sonoda, Hanamura, Okazaki, & Saitoh, 2005). | **Comments:** Should be used with caution in the clinical diagnosis of spatial neglect as it lacks sensitivity to the degree of impairment of the patients, such as for hemianopia and in assessing important everyday tasks in natural environments (Ferber & Karnath, 2001). |
| LOTCA, LOTCA-II, DLOTCA | N/A | **LOTCA Interrater reliability**: r = 0.82-0.97 (excellent) (Su et al., 2000; Zoltan, 2007)  **DLOTCA interrater reliability**: r = 0.98 (excellent) (Razemba, Jacobs, & Franzsen, 2017)  **Internal consistency:** alpha = 0.68-0.85 (adequate to excellent) for all domains except memory: r = 0.26 (poor) (Katz, Livni, Erez, & Averbuch, 2011). | A Wilcoxon two-sample test showed that all subtests differentiated at the 0.0001 level of significance between the patient groups and the control group, which supports the LOTCA's validity (Katz, Itzkovich, Averbuch, & Elazar, 1989). | **Comments:** DLOTCA is the latest version of the LOTCA. It includes mediating and cueing during administration, which helps determine the patient's potential for learning during rehabilitation (Razemba et al., 2017). |
| Manikin and Feature Profile subtests of the Arthur Point Scale of Performance Tests | N/A | **Test-retest reliability** for the Manikin subtests: r = 0.59-0.61 (adequate) and Feature Profile subtest: r = -0.1-0.53 (poor to adequate) (Titus et al., 1991). | N/A | **Other Psychometrics:** There were no statistically significant differences between the subjects with R or L lesions (Titus et al., 1991).  **Comments:** Very low test-retest reliability coefficients were found in this study for the Manikin subtest, therefore this test should not be used to assess change (Titus et al., 1991). |
| MAC | **Sensitivity:** The MAC detected neglect in 60.0-66.7% patients considered "recovered" based on paper-and-pencil tests, indicating that the MAC may be more sensitive in detecting neglect compared to neuropsychological assessment (Ten Brink, Visser-Meily, & Nijboer, 2018). | N/A | Correlation with shape cancellation: r = 0.53 (adequate), line bisection: r = 0.38 (adequate) and CBS total score r = 0.42 (adequate) (Ten Brink et al., 2018). MAC appears to be ecologically valid (Ten Brink et al., 2018). | **Other Psychometrics:** 81-82.8% patients showed neglect on both paper-and-pencil tests and the MAC, indicating that there is agreement between these tasks (Ten Brink et al., 2018). Despite the 60.0-66.7% patients showing neglect on the MAC but not the paper-and-pencil tests, 17.2-19% patients showed neglect on neuropsychological assessments, but not on the MAC (Ten Brink et al., 2018).  **Comments:** Task requires patients to be able to move independently through the corridor route, so motor impairment can affect performance (Ten Brink et al., 2018). One limitation is that the MAC can never be standardized across settings (Ten Brink et al., 2018). It is recommended administering the MAC in conjunction with neuropsychological assessment in order to assess the neglect in a dynamic way (Ten Brink et al., 2018). For psychometric properties, presence of neglect on neuropsychological assessments but absence on the MAC may be due to the differences in level of arousal needed to perform the different tests (Ten Brink et al., 2018). |
| MVPT & MVPT-3 | **Predictive Value: PPV** for prediction of on-road driving performance: With a cut- off score of 25: 74% (proportion of individuals with scores < 25 who failed road test), 53% for RH lesion and 41% for LH lesion (Korner-Bitensky et al., 2000). With a cut-off score 30: 60.9%-86.1%, 94% for RH lesion and 80% for LH lesion (Zeltzer, 2008a) . A **NPV** of 64.2% was found (low) (Zeltzer, 2008a). | All of the studies investigating reliability on the MVPT or MVPT-3 were only done on a children population. **Original MVPT Test-retest reliability:** r = 0.77-0.91 (excellent) (Colarusso & Hammill, 1972).                  **MVPT-3 Test-retest reliability:** r = 0.87-0.92 (excellent) (Colarusso & Hammill, 1972). **Internal consistency**. Alpha = 0.69-0.90 (poor to excellent) (Colarusso, & Hammill, 2003). | **Construct validity:** The degree of difference between the mean scores of individuals with stroke and without stroke calculated using effect size was ES = 0.67 and 0.54, suggesting that the MVPT can discriminate between individuals with and without stroke (York & Cermak, 1995). **Correlations was found between the MVPT and the following tests:** LOTCA subscales of Visuo-motor organization and Thinking operations: r = 0.60-0.72 (excellent); RPAB subscales of Sequencing, Figure Ground Discrimination and Spatial Awarenes: r = 0.39-0.72 (adequate to excellent); Frostig Developmental Test of Visual Perception: r = 0.38-0.60 (adequate to excellent) and DTVP: r = 0.27-0.74 (poor to excellent) (Brown, Rodger, & Davis, 2003; Su et al., 2000). | **Other Psychometrics:** Time of injury and time when the testing took place appears to be related, as participants who were tested in earlier weeks after their CVA scored lower in the MVPT. The study found a significant correlation between the time of diagnosis of the CVA and the MVPT scores (r = 0.38, p = 0.03) (Cate & Richards, 2000).  **Comments:** Scores on the MVPT seem not be strongly differentially sensitive to the hemispheric specialization in stroke population (Su et al., 2000). One study indicates that a combined use of the LOTCA, RPAB, and MVPT can provide a multidimensional screening of perceptual functions in stroke patients (Su et al., 2000). The predictive validity of the MVPTt is not sufficiently high to warrant its use as the sole screening tool in identifying those who are unfit to undergo an on-road evaluation (Korner-Bitensky et al., 2000). |
| MIQ-RS | N/A | **Test-retest reliability:** ICC = 0.83-0.99 (excellent) (Butler et al., 2012). **Internal consistency** of the visual and kinesthetic subscales: Cronbach α: 0.95-0.98 (excellent) (Butler et al., 2012). | Total variance: 88.6% for visual component and 83.4% for kinesthetic component for both able-bodied and stroke patients (Butler et al., 2012). | N/A |
| OT-APST | **Sensitivity:** Agnosia: 85.7%; Body scheme: 30.8%; Neglect: 69.2%; Constructional skills: 51.9%; Apraxia: 50.0% (Cooke, McKenna, Fleming, & Darnell, 2006).  **Specificity:** Agnosia: 66.1%; Body scheme: 95.7%; Neglect: 91.2%; Constructional skills: 71.4%; Apraxia: 84.9% (Cooke et al., 2006). | The OT-APST has high levels of interrater, intra-rater, and test-retest reliability for all items (Cooke et al., 2006). | **Concurrent Criterion validity:** (measured using Somers’s d correlations): Agnosia: 0.64 (0.13) (moderate); Body scheme: 0.27 (0.10) (fair); Neglect: 0.66 (0.08) (moderate); Constructional skills: 0.28 (0.12) (fair); Apraxia: 0.35 (0.12) (fair) (Cooke et al., 2006). **Convergent validity:** Has demonstrated correlations with the Lowenstein Occupational Therapy Cognitive Assessment (Cooke et al., 2006). The OT-APST subscale constructs appear to be distinct from the Cognistat and the DTVP-A (Brown et al., 2011). Only 3/10 Cognistat subscales were significantly correlated with OT-APST subscales (Cognistat Constructional ability and OT-APST Body Scheme; Cognistat Memory and OT-APST Functional Skills; and Cognistat Calculations and OT-APST Body Scheme) (Brown et al., 2011). Only 1 OT-APST subscale (Body Scheme), was significantly correlated with the DTVP-A’s Figure-Ground, Visual-Motor Search and Visual Closure subscales (Brown et al., 2011). The OT-APST Agnosia, Unilateral Neglect and Constructional Skills sub-scales had moderate to very high correlations with similar construct RPAB items (Colour Matching, Figure-Ground, Shapes Copying, Word Copying and Cube Copying) (Razemba et al., 2017). Body Scheme, Apraxia and Acalculia OT-APST subscales did not have significant correlations with any of the RPAB items (Razemba et al., 2017). **Criterion, Ecological and Construct validity**: have been demonstrated (Razemba et al., 2017). | **Other Psychometrics:** Likelihood ratios (calculated by the reviewers): *Positive LR:* Agnosia: 2.53; Body scheme: 7.16; Neglect: 7.86; Constructional skills: 1.81; and Apraxia: 3.31. *Negative LR:* Agnosia: 0.216; Body scheme: 0.723; Neglect: 0.338; Constructional skills: 0.674; and Apraxia: 0.589.  Accuracy: Agnosia: 71.2%; Body scheme: 72.6%; Neglect: 79.5%; Constructional skills: 57.3%; and Apraxia: 75.3% (Cooke et al., 2006).  **Comments:** The OT-APST demonstrates construct validity when compared to the DLOTCA and RPAB; therefore the OT-APST can be used as a perceptual and cognitive screening tool for English speaking stroke patients (Razemba et al., 2017) The OT- APST is not intended to assess visual perceptual skills in the healthy adult population (Cooke, McKenna, Fleming, & Darnell, 2005). The OT-APST has cultural bias limitations (Cooke, McKenna, Fleming, et al., 2005). The OT-APST is recommended to be used in addition to observational assessment of ADLs for stroke and ABI patients (Cooke, McKenna, & Fleming, 2005). Screening of primary visual skills (visual acuity, visual tracking, etc.) should be completed prior to OT-APST administration (Cooke, McKenna, & Fleming, 2005). If a visual field impairment is identified on visual screening, the OT-APST test procedure should be modified, and test materials should be placed in the patient's intact visual field (Cooke, McKenna, & Fleming, 2005). The OT-APST should be completed in its entirety to maintain its sensitivity as a screening tool, and for the reliability and validity of result interpretation (Cooke et al., 2006). |
| OSOT Perceptual Evaluation | **Sensitivity:** (differentiating between individuals with neurological impairment and neurotypical control): For the original version, at a total score cut-off of 110, sensitivity was 100%; At a cut-off of 100, the sensitivity was 73.8% [corrected value] (Boys, Fisher, Holzberg, & Reid, 1988). For the revised version, sensitivity was 100% with a cut-off score of 70 and over, and of 58% with a cut-off of 60 (Fisher, Boys, & Holzberg, 1991).  **Specificity:** (differentiating between individuals with functional impairment and a neurotypical control): For the original version, specificity was 40% for a total score cut-off of 110 (Boys et al., 1988). In the revised version, the specificity was 40% with a cut-off score of 70 and over, and 100% with a cut-off of 60 (Fisher et al., 1991).  **Predictive Value:** All predictive values were calculated by the reviewers. **PPV** for the 100 cut-offs: 100%. **NPV** for the 100 cut-offs: 76.9%. **PPV** for the 110 cut-offs: 65.6%. **NPV** for the 110 cut-offs: 100%. | **Internal consistency:** Original OSOT: Ranges from Cronbach's alpha = 0.23-0.95 (poor to excellent) (Boys et al., 1988). Revised: Cronbach's alpha = 0.90 (excellent) (Fisher et al., 1991). **Interrater reliability:** Original OSOT: r = 0.93 (excellent (Boys et al., 1988). | **Criterion (Concurrent) validity:** Correlations of the OSOT with the Physical Self-Maintenance Scale (PSMS), the Instrumental Activities of Daily Living Scale and the Mini-Mental State Evaluation (MMSE) ranged from 0.43-0.44 (adequate correlation) (Boyd & Dawson, 2000). The results of this study suggest that perceptual impairment is related to activities of daily living status (Boyd & Dawson, 2000). **Construct validity:** Moderate correlations were reported for the observed scores between each of the 6 domains of the original OSOT, demonstrating that each domain measures different concepts, which together give a global perceptual deficit score (Boys et al., 1988). | **Other Psychometrics:** Likelihood ratios (calculated by the reviewers): *Positive LR* for the 100 cut-offs: not calculable. *Negative LR* for the 100 cut-offs: 0.263. *Positive LR* for the 110 cut-offs likelihood ratios: 1.67. *Negative LR* for the 110 cut-offs: 0. *Positive LR* for the 70 and over cut-offs: 1.67. *Negative LR* for the 70 and over cut-offs: 0. *Positive LR* for the 60 cut-offs: not calculable. *Negative LR* for the 60 cut-offs: 0.42. |
| Read-Right | **Sensitivity:** 95-100% for the non-affected field (rarely falsely identified points as missed in the normally seeing field) and 55-100% for the affected hemifield (Koiava et al., 2012) .  **Specificity:** 100% for the non-affected field; and 75-100% for the affected hemifield (Koiava et al., 2012). | **Interrater reliability:** k values = 0.46-1 (adequate to excellent) (Koiava et al., 2012). | **Construct validity:** The intraclass correlation coefficient, collapsed across all 6 points in the affected hemi-field, was highly significant (0.884, p < 0.001(Koiava et al., 2012). A value > 0.75 indicates excellent correlation (Koiava et al., 2012). | **Comments:** Read-Right may be superior to longer algorithms (e.g., the Humphrey or the Goldman) when testing patients with poor attention, low acuity or those at the extremes of age (Koiava et al., 2012). Read-Right is not designed to replace standardised visual perimetry but may be useful as a quick and easy screening (Koiava et al., 2012). The test is available as part of two free to use web-based therapy applications (Koiava et al., 2012). |
| ROCF Test/ The Figure of Rey | **Sensitivity:** The ROCF test is particularly sensitive to diffuse damage after TBI (Ashton, Donders, & Hoffman, 2005), because the instrument is non-specific with respect to laterality (Ariza et al., 2006). | N/A | N/A | **Other Psychometrics:** Visuospatial memory recall scores, as assessed by ROCF recall, improved at 6 months (p = 0.013), but no differences were observed for ROCF copy (p = 0.657) (Zaninotto et al., 2017). Neither age, years of education, admission Glasgow Coma Scale (CGS), nor IQ influenced the ROCF recall improvement (Zaninotto et al., 2017). In prediction of stroke patients' ability to drive, the ROCF combined with a visual neglect assessment and an on-road test constituted the best model, accounting for 73.0% of the variance (Akinwuntan et al., 2006).  **Comments:** Often used as one measure in a battery to assess readiness to return to driving. |
| RightEye oculomotor tests | **Sensitivity:** 64% for Horizontal Saccades test (Hunfalvay et al., 2019) and 68% for Vertical Saccades test (Hunfalvay et al., 2020).  **Specificity:** 65% for Horizontal Saccades test (Hunfalvay et al., 2019) and 73% for Vertical Saccades test (Hunfalvay et al., 2020). | N/A | N/A | **Other Psychometrics:** Likelihood ratios (calculated by the reviewers): *Positive LR* for the Horizontal Saccades: 1.829. *Negative LR* for the Horizontal Saccades: 0.554. *Positive LR* for the Vertical Saccades: 2.519. *Negative LR* for the Vertical Saccades: 0.438.  **Comments:** The eye-tracking technology used to measure vertical saccadic eye movements is able to provide a timely, objective method of differentiating between individuals with moderate and severe TBI, but could not detect differences in performance of those with mTBI and no TBI (Hunfalvay et al., 2020). The sensitivity and specificity values for the horizontal and vertical saccade metrics are promising in differentiating TBI cases from no-TBI (Hunfalvay et al., 2019). Over the next 5–10 years, it is predicted that the use of eye-tracking tests will become commonplace to diagnose TBI, evaluate its severity, and monitor its recovery (Hunfalvay et al., 2019). |
| RightEye Vertical Smooth Pursuit test | **Sensitivity**: 68% (when used to differentiate between mTBI. moderate TBI and severe TBI) (Hunfalvay et al., 2020).  **Specificity:** 73% (when used to differentiate between mTBI, moderate TBI and severe TBI) (Hunfalvay et al., 2020). | N/A | Sn=68%; Sp=73%; Area under the curve (AUC)=0.772. Both Variance of gaze deviance and SP% (smooth pursuit) metrics revealed significant between group differences between mTBI and healthy controls (F (3, 88)=4.52; p=0.005 and F (3, 88)=3.80; p=0.013) respectively. Significant difference also found between moderate and severe TBI groups and the no-TBI group, but not between mild TBI and no-TBI groups. Conclusion: Vertical smooth pursuit eye movements measured through eye-tracking technology could accurately differentiate between individuals with moderate and severe levels of TBI (Hunfalvay et al., 2020). | **Other Psychometrics:** Likelihood ratios (calculated by the reviewers): *Positive LR:* 2.52. *Negative LR:* 0.438.  **Comments:** The Vertical Smooth Pursuit eye tracking test was able to distinguish between severe and moderate levels of TBI but unable to detect differences in the performance of participants with mTBI and healthy controls in one study (Hunfalvay et al., 2020). It is concluded that the eye-tracking technology used to measure VSP eye movements is able to provide a timely and objective method of differentiating between individuals with moderate and severe levels of TBI (Hunfalvay et al., 2020). |
| RPAB | **Sensitivity:** Most subtests of the RPAB lack sensitivity in the upper range, rendering a ‘‘ceiling effect, except for the Figure-Ground Discrimination and the Spatial Awareness subtests (Su et al., 2000). Many of the subtests are simple in nature so that the majority of patients obtain the maximum score, which suggests that they lack the sensitivity to detect anything other than gross deficits (Matthey, Donnelly, & Hextell, 1993). | **Interrater reliability:** r = 0.72-1.00 (excellent) (Bhavnani, Cockburn, Whiting, & Lincoln, 1983; Matthey et al., 1993). **Test-retest reliability:** r = 0.59-1.00 (adequate to excellent) (Bhavnani et al., 1983; Matthey et al., 1993). Small sample sizes (n = 6-19) were used to calculate the reliability coefficient, making the reliability data questionable (Bhavnani et al., 1983; Matthey et al., 1993) | The RPAB manual suggests that the RPAB’s validity is supported by its correlation with other tests of perception and its ability to discriminate between brain-damaged and normal subjects (Whiting, Lincoln, Bhavnani, & Cockburn, 1986) | **Other Psychometrics:** The findings of the study suggest that RPAB severity scores do not significantly predict functional performance, but the presence of a visual perceptual disorder as assessed by the RPAB (failure on three or more subtests) is significant in predicting functional performance and FIM discharge scores (Donnelly, 2002). The presence or absence of a visual perceptual disorder produces approximately a 10-point (9.67) difference on the discharge FIM score (Donnelly, 2002).  **Comments:** The RPAB can be used as an outcome measure to monitor progress in perceptual status over time (Matthey et al., 1993). Scores on the RPAB seem to not be strongly differentially sensitive to the hemispheric specialization in stroke. A study indicates that a combined use of the LOTCA, RPAB, and MVPT can provide a multidimensional screening of perceptual functions in stroke patients (Su et al., 2000). Other authors however indicate that the RPAB may be limited for use as a screening as it may be too lengthy and does not evaluate all major constructs (Cooke, McKenna, & Fleming, 2005). |
| Scan Board Test | N/A | **Interrater and test-retest reliability:** statistically significant Spearman rank order correlations (Warren, 1990). | Differences in performance between the hemiplegic and control groups were statistically significant for all tests as the hemiplegic group achieved lower scores (Warren, 1990). | N/A |
| SAW | N/A | N/A | **Construct validity** was supported both by test scores distinguishing subjects with right-side brain injury from other subjects, and by means of a factor analysis (Van Deusen, 1988). 2 factors were clearly identified: left spatial hemi imperception and lateral eye movement efficiency, that is, efficient eye function in scanning stimuli (Van Deusen, 1988). 2 foveal imperception factors were less clear (Van Deusen, 1988). | N/A |
| Semi-Structured Scale for the Functional Evaluation of Hemi-inattention Evaluation | N/A | **Internal consistency (inter-item correlations):** Personal subscale: r = 0.57-0.62 (adequate); Extrapersonal subscale: r = 0.44-0.71 (adequate) (Zoccolotti, Antonucci, & Judica, 1992). **Interrater reliability:** Personal subscale: r = 0.88 (excellent); Extrapersonal subscale: r = 0.96 (excellent) (Zoccolotti et al., 1992)**.** However, in this study, raters underwent intense training, which may limit the generalizability of these findings. | **Construct (Concurrent) validity:** The personal and extrapersonal subscales were compared with performance on 4 standard diagnostic tests for USN (Line Cancellation Test, Letter Cancellation Test, Wundt-Jastrow Area Illusion Test, and Sentence Reading Test). The extrapersonal subscale correlated with each conventional test (kendall tau = -0.60; -0.52; 0.20; and -0.40, respectively). Performance on the personal subscale did not correlate with performance on these conventional tests, which suggests that this subscale measures different aspects of neglect and requires further validation(Zoccolotti et al., 1992). | N/A |
| Short assessment battery (Akinwuntan et al., 2007) | **Sensitivity**: 77% (Akinwuntan et al., 2007)  **Specificity:** 92% (Akinwuntan et al., 2007).  **Predictive Value:** **PPV:** 87% (probability of actually failing when predicted to fail based on performance). **NPV:** 86% (Probability of actually passing when predicted to pass) (Akinwuntan et al., 2007). | **Interrater reliability:** k range = 0.44-0.78 (adequate) (Akinwuntan et al., 2005). **Item-per item reliability:** ICC = 0.63-0.87 (adequate to excellent) (Akinwuntan et al., 2005). **Reliability of the overall performance in the road test**: ICC = 0.83 (excellent) (Akinwuntan et al., 2005) | **Criterion validity** of the road test: 78.9% of the subjects were correctly classified when the judgments of the principal evaluator were compared with outcomes of the SDSA (Akinwuntan et al., 2005). | **Other Psychometrics:** 81.6% agreement in classification between the principal evaluator and a state-registered evaluator’s judgments (Akinwuntan et al., 2005).  Likelihood ratios (calculated by the reviewers): *Positive LR:* 9.94*. Negative LR:* 0.255.  **Comments:** This short assessment battery is a good predictor of fitness-to-drive in stroke survivors with moderate physical and cognitive impairments (Akinwuntan et al., 2007). This finding suggests that 128 is the cut-off point in the standardized on-road test for a chance to be found fit-to-drive after a stroke in Belgium (Akinwuntan et al., 2007). All 4 visual tests were performed on Ergovision equipment (Akinwuntan et al., 2007). |
| SLCT | N/A | **Test-retest reliability:** r = 0.63 (adequate) (Gordon et al., 1984). | **Construct validity:** Correlation with mean CT-scan damage was found to be r = -0.35 (adequate) (Egelko et al., 1988). Correlations between the SLCT and other visuo-spatial tests (Albert’s Test, Sentence Reading Test, and the Wundt-Jastrow Area Illusion Test) ranged from r = 0.36-0.69 (adequate to excellent) (Zoccolotti et al., 1989). Correlation with the Extra-personal subscale of Semi-Structured Scale for the Functional Evaluation of Hemi-inattention (Kendal’s tau = -0.52) was also established (Zoccolotti et al., 1992). | **Other Psychometrics:** Normative data has been published by sex and age, based on the results from 341 patients with lesions of the right hemisphere (Gordon et al., 1984).  **Comments:** No training is required for administration. This test requires the ability to recognize letters of the alphabet. It should not be used to differentiate between sensory neglect and motor neglect because it requires both visual search and manual exploration (Làdavas, 1994). |
| SRWL | N/A | N/A | **Construct validity** was supported both by test scores distinguishing subjects with right-side brain injury from other subjects, and by means of a factor analysis (Van Deusen, 1988). 2 factors were clearly identified: left spatial hemi imperception and lateral eye movement efficiency, that is, efficient eye function in scanning stimuli (Van Deusen, 1988). 2 foveal imperception factors were less clear (Van Deusen, 1988). | N/A |
| St. Marys CVA Evaluation Battery | N/A | N/A | Results support the construct validity with a Chi-square of 9.949 for this test, with a probability of 0.0069 (Harlowe & Van Deusen, 1984). | **Other Psychometrics:** From the results of this analysis, it is apparent that the perceptual test scores of these patients did differentiate discharge location well beyond the 0.05 level (Harlowe & Van Deusen, 1984). |
| SCT | **Sensitivity:** 76.4% (most sensitive of the BIT battery) (Bailey et al., 2000; Harlowe & Van Deusen, 1984). It appears to be less sensitive than the Apples Test in identifying patients with milder signs of inattention (Bickerton et al., 2011). | **Interrater reliability:** r = 0.99 (excellent) (Bailey et al., 2004). **Test-retest reliability:** r = 0.99 (excellent) (Bailey et al., 2004). | **Construct convergent validity:** Correlation with Line Bisection Test: r = -0.33 (adequate), with the Albert's test: r = 0.63 (adequate) (Agrell et al., 1997), and the eating item of the CBS r = -0.83 (excellent) (Luukkainen-Markkula et al., 2011). The Star Cancellation Test is a highly valid test of UN that correlates with functional abilities and predicts long-term outcome (Toglia & Cermak, 2009). | **Other Psychometrics:** Star Cancellation predicted 61% of the variance in the FIM and RKE-R scores (ADL and iADL tests), suggesting that this individual subtest has the same predictive power as the total BIT score (Katz, Hartman-Maeir, Ring, & Soroker, 1999).  **Comments:** SCT is referred to as the gold standard (Bickerton et al., 2011). The maximum possible number of stars for the patient to cross is54, 27 on each side of the page. Scores between 0 and 0.46 indicated left-sided neglect, and 0.54–1 indicated right-sided neglect (Bailey et al., 2000). The Star Ratio can provide clinicians with a useful measure of the lateralized extent and severity of omissions in contra-lesional space (Bailey et al., 2000). For the Star Cancellation Test, a study suggested that neglect is present if individuals omit more than 15 stars, or omit between 6 and 15 stars with more than twice the number of omissions located on one side of the page (Stone et al., 1991). |
| SNAP | **Sensitivity:** 68% compared with the VSB task (Leibovitch et al., 2012).  **Specificity:** 76% compared with the VSB task (Leibovitch et al., 2012). | **Internal consistency:** Cronbach’s coefficient α = 0.84, p < 0.0005 (excellent) (Leibovitch et al., 2012). | **Concurrent Criterion validity:** Area under the curve = 0.78, p < 0.001 (adequate) using the VSB as the comparator (Leibovitch et al., 2012). | **Other Psychometrics:** Likelihood ratios (calculated by the reviewers): *Positive LR:* 2.83. *Negative LR:* 0.421.  **Comments:** The test is recommended to use as a beside battery in the acute phase of stroke (Leibovitch et al., 2012). |
| TOJ test | **Sensitivity:** When compared to the line bisection and shape cancellation tests, around 20% of patients performed worse than the cut-off score, indicating that they showed deficits on that particular test (Van der Stigchel & Nijboer, 2018). This percentage was twice as high compared to the TOJ test, suggesting that the TOJ is less sensitive than traditional tests in assessing a broad range of neglect symptoms, and may be sensitive to deficits in the spatial bias only (Van der Stigchel & Nijboer, 2018). | N/A | Correlation between tests: Shape cancellation vs TOJ: r = - 0.39 (adequate); Line bisection vs. TOJ: r = - 0.211 (poor) (Van der Stigchel & Nijboer, 2018). | **Other Psychometrics:** The cut-off score was validated using Crawford and Howell’s significance test on differences between individual’s score and control sample, which confirmed that this cut-off score is indeed significantly different from the control sample (t = 2.52; p = 0.02; estimated percentage of normal population falling below individual’s score: 98.95%) (Van der Stigchel & Nijboer, 2018).  **Comments:** The TOJ test might be used as a complement to the line bisection and shape cancellation tests (Van der Stigchel & Nijboer, 2018). The TOJ test does not depend on the reaction time but requires a simple non speeded response to which of the two stimuli arrived earlier (Van der Stigchel & Nijboer, 2018). Results indicated that the strength of the spatial bias was strongly correlated with an object cancellation test, but not with a line bisection test (Van der Stigchel & Nijboer, 2018). This may be explained by the fact that contrary to shape cancellation, successful performance on line bisection tests depends primarily on an object-based, allocentric representation of space and is unrelated to any spatial bias (Van der Stigchel & Nijboer, 2018). |
| NPC | N/A | **Interrater reliability:** Excellent with children (Rouse, Borsting, & Deland, 2002). **Intra-rater reliability:** ICC = 0.94 to 0.98 (excellent) with children (Rouse et al., 2002). **Test-retest reliability:**  ICC = 0.89 to 0.92 (excellent) with children (Rouse et al., 2002). | N/A | N/A |
| Test of Three-Dimensional Constructional Praxis (3rd edition) | N/A | **Test-retest reliability:** r = 0.82 (excellent) (Titus et al., 1991). | N/A | **Other Psychometrics:** There were no statistically significant differences between the subjects with right or left lesions (Titus et al., 1991).  **Comments:** The Test of Three-Dimensional Constructional Praxis was one of the most discriminating in differentiating compared to other tests on this sample stroke patients from normative samples on perceptual performance (Titus et al., 1991). It was also one of the perceptual tests showing the most consistent correlation with activities of daily living, therefore occupational therapists might consider using it in order to identify perceptual performance deficits that might affect performance on ADLs (Titus et al., 1991). |
| TVPS & TVPS-3 | **Sensitivity:** 77.3% for the TVPS, not sensitive to the side of stroke (Su, Chien, Cheng, & Lin, 1995). No studies investigated on the sensitivity of the TVPS-3.  **Specificity:** 71.6% for the TVPS (Su et al., 1995). No studies investigated on the specificity of the TVPS-3.  **Predictive Value (calculated by the reviewers):** **PPV:** 27.9%. **NPV:** 95.7%. | **Test-retest reliability for TVPS**: ICC = 0.92 (excellent) for the overall scale (Chiu, Wu, Chou, Yu, & Hung, 2016). ICC = 0.53-0.82 (adequate to excellent) for subscales (Chiu et al., 2016). | **Construct validity for TVPS:** Subjects with CVA's significantly lower mean accuracy scores compared to control subjects (Su et al., 1995). **Criterion validity for TVPS**: moderate relationship between the TVPS and the Picture Completion subtest of the Wechsler Intelligence Scale, the Bender Visual-Motor Gestalt Test, and the Developmental Test of Visual-Motor Integration (Su et al., 1995). **Ecological validity for TVPS-3:** r = 0.40 (adequate) of the TVPS-3 overall scale with the Frenchay Activities Index (FAI) and r = 0.48 (adequate) of the TVPS-3 overall scale with the BI (Chiu et al., 2019). **Convergent validity for TVPS-3:** r = 0.60 (excellent) of TVPS-3 overall scale with the MMSE, and r = 0.49–0.68 (adequate to excellent) with 7 WCST indices (Chiu et al., 2019). **Discriminative validity for TVPS-3:** excellent discriminative validity at the overall scale and adequate discriminative validity at the subscale level between the 2 groups with different levels of disability (Chiu et al., 2019). | **Other Psychometrics:** Likelihood ratios (calculated by the reviewers): *Positive LR:* 2.72. *Negative LR:* 0.317.  **Comments:** The TVPS has been standardized on 962 school children but has not yet been standardized on adults (Su et al., 1995). Even though the test was standardized with children, it may be useful in screening for Visual-perceptual deficits in adult patients with brain damage (Su et al., 1995). Several advantages of the TVPS for use with our older patient sample are identified: bold lines that form the designs are less stressful to the vision; the test is not confounded by verbal and motor impairments commonly associated with persons who have had stroke; and the test administration is not complicated (Su et al., 1995). Concern that the TVPS may be unreliable for older adults because of ceiling effects was not supported by the results Su et al. (Su et al., 1995). Median subtest intercorrelations (18 to 0.40) was found to be low, indicating each subtest assesses a unique aspect of visual perception (Su et al., 1995). Correlations between TVPS performance and age across subtests ranged from 0.67 to 0.77, indicating a substantial relationship between these 2 variables, although standardized on children (Su et al., 1995). |
| Tobii glasses eye-tracking while performing a task (making a cup of coffee) | **Sensitivity:** 100% (tested on 6 patients) (Kortman & Nicholls, 2016). | N/A | **Concurrent validity** of Tobii glasses as a measure of USN: Correlation with Bells test and time taken to search on the right r = 0.622 (excellent) (Kortman & Nicholls, 2016). | **Comments:** The findings of this study suggest that eye-tracking glasses may be a way for OT to objectively and sensitively detect USN dynamically using relevant daily occupations (Kortman & Nicholls, 2016). |
| TMT | **Sensitivity:** In a study comparing performance of stroke vs healthy control, Tippett et al. (2013) found no between group differences regarding visual-spatial/visual-motor function reaction times both in pen and paper and a computerized visuomotor procedure (F1,18 = p >0.05). However, when examining the performance of individuals at two different point in time on the same measure (inter-individual variability) significant between group differences in variance, with stroke group vs controls having greater variability for the Trails A (2.43 (95% CI, −153.04 to −9.92), p=0.028) and Trails B (2.9 (95% CI, −78.26 to −12.35), p=0.010). Reaction time and coefficient of variability CbVM for stroke vs controls found significant between group differences in variability, with stroke group vs controls having greater coefficient of variation for initiation time (0.45 vs. 0.22) and movement times (0.38 vs. 0.24) (Tippett et al., 2013). | **Interrater reliability:** r = 0.94 (excellent) for Trails A and r = 0.90 (excellent) for Trails B (Lezak, Howieson, Loring, & Fischer, 2004).                **Test-retest reliability:** r = 0.78 (excellent) for Trails A and r = 0.67 (adequate) for Trails B for patients with stroke (Matarazzo, Wiens, Matarazzo, & Goldstein, 1974). | **Content validity (Face validity)**: TMT A and TMT B were both significant, in accordance with other pathologies tests that assessed attention in visual-spatial tasks and visual exploration, and were correlated with driving ability (Saviola et al., 2018). **Criterion validity (Predictive validity):** For TMT B, 85% (high positive predictive value) and 48% (low negative predictive value) for a successful completion of driving evaluation with a cut-off score of < 3 errors (Marshall et al., 2007; Mazer, Korner-Bitensky, & Sofer, 1998; Mazer et al., 2003). Participants who scored poorly on both the MFVP and TMT Part B had 22 times the likelihood of failing the on-road evaluation (Marshall et al., 2007; Mazer et al., 1998; Mazer et al., 2003). TMT B was one of the best predictors for on-road driving test outcome post-stroke (Marshall et al., 2007; Mazer et al., 1998; Mazer et al., 2003). **Construct validity (Convergent/Discriminant validity):** r = 0.30-0.44 (poor to adequate) with Category Test, WCST, Paced Auditory Serial Addition Task, and Visual Search and Attention Test (O'donnell, Macgregor, Dabrowski, Oestreicher, & Romero, 1994). Strong correlations with other timed executive dysfunction tests, such as with the UFOV (Barker-Collo, Feigin, Lawes, Parag, & Senior, 2010; Calvanio et al., 2004). **Construct validity (Known groups validity):** Highly significant differences in mean and sum scores were found between the two groups (p < 0.001) on both parts of the TMT, suggesting that the TMT is able to differentiate between patients with and without brain damage (Reitan, 1955). | **Other Psychometrics:** Floor and ceiling effect: Significant ceiling effects for Part A, no ceiling effects for part B (Mazer et al., 1998).  Responsiveness: Able to detect an improvement in attention at 6 weeks and 6 months following stroke (Barker-Collo et al., 2010).    **Comments:** Often used as one measure in a battery to assess readiness to return to driving. |
| UFOV | N/A | **Test-retest reliability:** adequate reliability (ICC 0.70) (Mazer, Sofer, Korner-Bitensky, & Gelinas, 2001). | **Convergent validity:** Internally, UFOV subtest 2 (UF2) correlated strongly with the other 2 subtests (UF1, UF3) (Calvanio et al., 2004). Externally, strong correlations were found between the UF2 test (measure of divided visual attention) and 6 paper-and-pencil measures of visual attention (visual span forward total score, visual span backward total score, TMT-A time to completion, TMT-B number correct, TMT-B time to completion, digit symbol number correct) (Calvanio et al., 2004). The UF1 and UF3 did not significantly correlate with the line cancellation number correct, line cancellation time to completion, letter cancellation number correct, letter cancellation time to completion, TMT-A number correct (Calvanio et al., 2004). **Concurrent Validity:** The UF2 predicted 52% of the FIM change and 60% of the length of stay (inpatient) variance (Calvanio et al., 2004). | **Other Psychometrics:** UFOV subtest 2 has been found to be highly sensitive in its ability to identify stroke patients who were unable to pass a subsequent standardized on-road test (George & Crotty, 2010).  **Comments:** Often used as one measure in a battery to assess readiness to return to driving. |
| VOMS tool | N/A | **Internal consistency** is high with a Cronbach α of 0.97 in youth and collegiate athletes (Kontos, Sufrinko, Elbin, Puskar, & Collins, 2016; Moran, Covassin, Elbin, Gould, & Nogle, 2018; Mucha et al., 2014). | Correlation of the VOMS with the Post-Concussion Symptom Scale was found to be significant (Mucha et al., 2014). | **Comments:** The VOMS tool is to be used in conjunction with other assessments (e.g., neurocognitive, symptoms, balance) and a comprehensive clinical examination, interview, and medical history (Kontos et al., 2016). |
| Virtual Wheelchair Navigation Skills | N/A | N/A | **Construct validity (convergent):** Significant correlations for simple and complex array, for participant and examiner driven for Bells Test, Letter Cancellation, Line Bisection, Picture and Menu reading (p < 0.01); Moss-Magee Wheelchair Navigation Test and simple array participant driven only (p < 0.01) (Buxbaum et al., 2008). | N/A |
| VRST | N/A | Research evidence demonstrates that the VRST has a high internal reliability (George, Clark, & Crotty, 2008). | **Construct validity:** The study results indicate that the VRST measures the construct of identification (inspection time), of the speed of information processing system and of the ability to extract visual information in a brief glance (George et al., 2008). The VRST does not measure the process of response after identification of the stimulus has occurred (George et al., 2008). **Predictive validity**: Results on the VRST were significantly associated with the on-road result. The VRST was able to predict those people most likely to be recommended, by a driver-trained occupational therapist, to pass or require lessons from their on-road evaluation (George et al., 2008). | **Comments:** The VRST provides useful clinical information about people with stroke but has not been validated specifically with the stroke group yet (George et al., 2008). The VRST predicted people most likely to be recommended, by a driver-trained OT, to pass or require lessons from their on-road evaluation, which may be useful clinically to indicate best timing for driving to be evaluated during the rehabilitation program (George et al., 2008). This research indicates that the VRST, is a potentially valid measure for inclusion in the off-road driver rehabilitation program for people with stroke (George et al., 2008). |
| VSA | N/A | N/A | VSA is a valid measure of the constructs of information processing and visual scanning (George et al., 2008). | **Comments:** The VSA should be further evaluated for consideration of inclusion in the occupational therapy driver rehabilitation program (George et al., 2008). |
| VSRT or Pepper Test | N/A | **Test-retest reliability:** 0.90 (excellent) for reading accuracy rate and 0.97 (excellent) for corrected reading in a group of patients with central field loss (Blaylock, Warren, Yuen, & DeCarlo, 2016). **Internal consistency:** 0.82 (excellent) for participants with homonymous hemianopia and 0.69 (acceptable) for quadrantanopia (Blaylock et al., 2016). | **Construct validity:** r = 0.82 (p < 0.05) (excellent) for the reading rates of VSRT (Blaylock et al., 2016; Zoltan, 2007). VSRT can discriminate between readers with normal sight and readers with a visual field deficit (Blaylock et al., 2016). Similar results were obtained when comparing the corrected reading rate of patients with homonymous hemianopia (t568 = 9.81; P < 0.0001) or those with quadrantanopia (t554 = 3.95; P < 0.0001) (Blaylock et al., 2016). | N/A |
| VV assessment | N/A | **Test-retest reliability:** ICC = 0.029-0.933 (poor to excellent) (Piscicelli, Nadeau, Barra, & Pérennou, 2015). | N/A | **Comments:** 6–10 trials are recommended to obtain reliable VV orientation in subacute stroke patients (Piscicelli et al., 2015). |
| VRLAT | **Sensitivity:** USN was detected by the VRAT in 56% patients (in comparison to conventional tests that detected 50%) (Buxbaum, Dawson, & Linsley, 2012). | **Internal Consistency:** 3 levels of the test had a Chronbach’s alpha = 0.97 (excellent); corrected item total correlation: 0.92 (excellent) (Buxbaum et al., 2012). | **Concurrent criterion validity:** significant correlation of VRLAT left sided scores with standard measures of USN and collisions in the Real World Navigation Test (Buxbaum et al., 2012).               **Construct validity (convergent):** Correlations of the VRLAT with personal neglect measure: r = 0.41 (adequate), Bells Test cancel left: r = 0.43 (adequate); Letter Cancel Left and Right: r = 0.59 and 0.49 (adequate) (Buxbaum et al., 2012).              **Content and Face validity:** The task provides virtual replication of many of the tasks demands that are difficult for patients with USN (Buxbaum et al., 2012). | **Other Psychometrics:** VRLAT is equally likely to categorise patients with and without visual deficits as having neglect (Buxbaum et al., 2012). |
| VR-DiSTRO | **Sensitivity:** Total score: 100%; Sensitivity for subtests: SCT: 54%; LBT: 33%; BTT: 100%; EXT: 100% (Fordell, Bodin, Bucht, & Malm, 2011).  **Specificity:** Total score: 82%; Specificity for subtests: SCT: 96%; LBT: 100%; BTT: 86%; EXT: 95% (Fordell et al., 2011). | N/A | **Construct validity (convergent):** Correlation of VR-BTT and VR-EXT with BIT: r = 0.8 and 0.88 (excellent) (Fordell et al., 2011). | **Other Psychometrics:** Likelihood ratios: *Positive LR***:** total score: 5.5; SCT: 12.22; LBT: not calculable; BTT: 7.33; and EXT: 21.0 (Fordell et al., 2011). *Negative LR (calculated by the reviewers):* total score: 0; SCT: 0.479; LBT: 0.67; BTT: 0; and EXT: 0. |

ADLs, Activities of Daily Living; CBS, Catherine Bergego Scale; LR, Likelihood Ratio; N/A, Not Applicable; R, Right; L, Left; BTT, Baking Tray Test; BEN, Batterie d’Évaluation de la Négligence spatiale; SMT, Snellgrove Maze Test; PPV, Positive Predictive Value; NPV, Negative Predictive Value; BITC, Conventional Behavioral Inattention Test; BLO, Benton Judgement of Line Orientation; WAIS-R, Wechsler Adult Intelligence Scale-Revised; BIVSS, Brain Injury Vision Symptom Survey; TBI, Traumatic Brain Injury; VD, Visual Deficits; CDT, Clock Drawing Test; SNAP, Sunnybrook Neglect Assessment Procedure; MMSE, Mini-Mental State Examination; FIM, Functional Independence Measure; CTT, Color Trails Test; TMT, Trail Making Test; CbVM, Computer-based Visuomotor Task; MT, Movement time; IT, Initiation Time; VISSTA, Visual Spatial Search Task; RHD, Right Hemisphere Damaged; USN, Unilateral Spatial Neglect; HR, Hit Rate; MWCT, Mesulam-Weintraub Cancellation Test; LHD, Left Hemisphere Damaged; RT, Reaction Time; CHEERS, Craig Hospital Eye Evaluation Rating Scale; OT, Occupational Therapist; ABI, Acquired Brain Injury; DEM, Developmental Eye Movement; DVPT-A, Developmental Test of Visual Perception – Adolescent and Adult; OT-APST, Occupational Therapy Adult Perceptual Screening Test; DAT, Doorway Accuracy Test; DLCT, Double Letter Cancellation Test; CT, Computerized Tomography; ANT, Attention Network Test; CVLT-II, California Verbal Learning Test 2nd Edition; mTBI, mild Traumatic Brain Injury; FA, Fractional Anisotropy; GST, Gaze Stabilization Test; ABC, Activities-specific Balance Confidence; DHI, Dizziness Handicap Inventory; ImPACT, Immediate Post-Concussion Assessment and Cognitive Test; VOR, Vestibular Ocular Reflex; GEMAT, Gedachtnis Markaufsamkeit Test; GTT, GEMAT Total Time; SD, Standard Deviation; RH, Right Hemisphere; HVST, Halifax Visual Scanning Test; HVOT, Hooper Visual Organization Test; KVIQ, Kinesthetic and Visual Imagery Questionnaire; ICC, Intraclass Correlation Coefficient; VOMS, Vestibular/Ocular-Motor Screening; TOJ, Temporal Order Judgement; RPAB, Rivermead Perceptual Assessment Battery; UN, Unilateral Neglect; LOTCA, Loewenstein Occupational Therapy Cognitive Assessment; DLOTCA, Dynamic Loewenstein Occupational Therapy Cognitive Assessment; MAC, Mobility Assessment Course; MVPT, Motor-Free Visual Perception Test; LH, Left Hemisphere; CVA, Cerebral Vascular Accident; MIQ-RS, Movement Imagery Questionnaire-Revised; OSOT, Ontario Society Occupational Therapists; ROCF, Rey–Osterrieth Complex Figure; IQ, Intelligence Quotient; SAW, Search-A-Word; SDSA, Stroke Driver Screening Assessment; SLCT, Single Letter Cancellation Test; SRWL, Speeded Reading of Word Lists; SCT, Star Cancellation Test; iADL, Instrumental Activities of Daily Living; VSB, Visual Search Board; NPC, Near Point Convergence; TVPS, Test of Visual Perceptual Skills; WCST, Wisconsin Card Sort Test; UFOV, Useful Field Of View; VRST, Visual Recognition Slide Test; RKE-R, Rabideau Kitchen Evaluation – Revised; VSA, Visual Scanning Analyser; VSRT, Visual Skills for Reading Test; VV, Visual Vertical; VRLAT, Virtual Reality Lateralised Attention Test; LBT, Line Bisection Test; EXT, Visual Extinction

References

Adunsky, A., Fleissig, Y., Levenkrohn, S., Arad, M., & Noy, S. (2002). Clock drawing task, mini-mental state examination and cognitive-functional independence measure: Relation to functional outcome of stroke patients. *Archives of Gerontology and Geriatrics, 35*(2), 153-160. doi: 10.1016/S0167-4943(02)00018-3

Agrell, B., Dehlin, O., & Dahlgren, C. (1997). Neglect in elderly stroke patients: A comparison of five tests. *Psychiatry and Clinical Neurosciences, 51*(5), 295-300. doi: 10.1111/j.1440-1819.1997.tb03201.x

Akinwuntan, A., Feys, H., De Weerdt, W., Baten, G., Arno, P., & Kiekens, C. (2006). Prediction of driving after stroke: A prospective study. *Neurorehabilitation and Neural Repair, 20*(3), 417-423. doi:10.1177/1545968306287157

Akinwuntan, A., De Weerdt, W., Feys, H., Baten, G., Arno, P., & Kiekens, C. (2005). The validity of a road test after stroke. *Archives of Physical Medicine and Rehabilitation, 86*(3), 421-426. doi:10.1016/j.apmr.2004.04.047

Akinwuntan, A., Devos, H., Feys, H., Verheyden, G., Baten, G., Kiekens, C., & De Weerdt, W. (2007). Confirmation of the accuracy of a short battery to predict fitness-to-drive of stroke survivors without severe deficits. *Journal of Rehabilitation Medicine, 39*(9), 698-702. doi:10.2340/16501977-0113

Antona, B., Gonzalez, E., Barrio, A., Barra, F., Sanchez, I., & Cebrian, J. (2011). Strabometry precision: Intra-examiner repeatability and agreement in measuring the magnitude of the angle of latent binocular ocular deviations (heterophorias or latent strabismus). *Binocular Vision & Strabology Quarterly, Simms-Romano's, 26*(2), 91.

Ariza, M., Pueyo, R., Junqué, C., Mataró, M., Poca, M., Mena, M., & Sahuquillo, J. (2006). Differences in visual vs. verbal memory impairments as a result of focal temporal lobe damage in patients with traumatic brain injury. *Brain Injury, 20*(10), 1053-1059. doi:10.1080/02699050600909862

Ashton, L., Donders, J., & Hoffman, N. (2005). Rey Complex Figure Test performance after traumatic brain injury. *Journal of Clinical and Experimental Neuropsychology, 27*(1), 55-64. doi:10.1080/138033990513636

Azouvi, Marchal, Samuel, C., Morin, Renard, C., Louis-Dreyfus, A., . . . Bergego. (1996). Functional consequences and awareness of unilateral neglect: Study of an evaluation scale. *Neuropsychological Rehabilitation, 6*(2), 133-150. doi:10.1080/713755501

Azouvi, P., Bartolomeo, P., Beis, J., Perennou, D., Pradat-Diehl, P., & Rousseaux, M. (2006). A battery of tests for the quantitative assessment of unilateral neglect. *Restorative Neurology & Neuroscience, 24*(4), 273-285.

Azouvi, P., Olivier, S., De Montety, G., Samuel, C., Louis-Dreyfus, A., & Tesio, L. (2003). Behavioral assessment of unilateral neglect: Study of the psychometric properties of the Catherine Bergego Scale. *Archives of Physical Medicine and Rehabilitation, 84*(1), 51-57. doi:10.1053/apmr.2003.50062

Azouvi, P., Samuel, C., Louis-Dreyfus, A., Bernati, T., Bartolomeo, P., Beis, J., . . . Rousseaux, M. (2002). Sensitivity of clinical and behavioural tests of spatial neglect after right hemisphere stroke. *Journal of Neurology, Neurosurgery & Psychiatry*, 160-166. doi:10.1136/jnnp.73.2.160

Bailey, M., Riddoch, M., & Crome, P. (2000). Evaluation of a test battery for hemineglect in elderly stroke patients for use by therapists in clinical practice. *Neurorehabilitation, 14*(3), 139-150. doi:10.3233/NRE-2000-14303

Bailey, M., Riddoch, M., & Crome, P. (2004). Test–retest stability of three tests for unilateral visual neglect in patients with stroke: Star Cancellation, Line Bisection, and the Baking Tray Task. *Neuropsychological Rehabilitation, 14*(4), 403-419. doi:10.1080/09602010343000282

Barco, P., Wallendorf, M., Snellgrove, C., Ott, B., & Carr, D. (2014). Predicting road test performance in drivers with stroke. *American Journal of Occupational Therapy, 68*(2), 221-229. doi:10.5014/ajot.2014.008938

Barker-Collo, S., Feigin, V., Lawes, C., Parag, V., & Senior, H. (2010). Attention deficits after incident stroke in the acute period: Frequency across types of attention and relationships to patient characteristics and functional outcomes. *Topics in Stroke Rehabilitation, 17*(6), 463-476. doi:10.1310/tsr1706-463

Basagni, B., De Tanti, A., Damora, A., Abbruzzese, L., Varalta, V., Antonucci, G., . . . Mancuso, M. (2017). The assessment of hemineglect syndrome with cancellation tasks: a comparison between the Bells test and the Apples test. *Neurological Sciences, 38*(12), 2171-2176. doi:10.1007/s10072-017-3139-7

Baum, B. (1981). *The establishment of reliability and validity of a perceptual evaluation on a sample of adult head trauma patients.* (Doctoral dissertation). University of Southern California.

Beis, J., Keller, C., Morin, N., Bartolomeo, P., Bernati, T., Chokron, S., . . . Azouvi, P. (2004). Right spatial neglect after left hemisphere stroke: Qualitative and quantitative study. *Neurology, 63*(9), 1600-1605. doi:10.1212/01.WNL.0000142967.60579.32

Bergego, C., Azouvi, P., Samuel, C., Marchal, F., Louis-Dreyfus, A., Jokic, C., . . . Deloche, G. (1995). Validation d'une échelle d'évaluation fonctionnelle de l'héminégligence dans la vie quotidienne: l'échelle CB. *Annales de Réadaptation et de Médecine Physique,* 38(4), 183-189. doi:10.1016/0168-6054(96)89317-2

Bhavnani, G., Cockburn, J., Whiting, S., & Lincoln, N. (1983). The reliability of the Rivermead Perceptual Assessment and implications for some commonly used assessments of perception. *British Journal of Occupational Therapy, 46*(1), 17-19.

Bickerton, W., Samson, D., Williamson, J., & Humphreys, G. (2011). Separating forms of neglect using the Apples Test: Validation and functional prediction in chronic and acute stroke. *Neuropsychology, 25*(5), 567-580. doi:10.1037/a0023501

Binder, J., Marshall, R., Lazar, R., Benjamin, J., & Mohr, J. (1992). Distinct syndromes of hemineglect. *Archives of Neurology, 49*(11), 1187-1194. doi:10.001/archneur.1992.00530350109026

Blaylock, S., Warren, M., Yuen, H., & DeCarlo, D. (2016). Validation of a reading assessment for persons with homonymous hemianopia or quadrantanopia. *Archives of Physical Medicine & Rehabilitation, 97*(9), 1515-1519. doi:10.1016/j.apmr.2016.02.022

Bohannon, R. (2003). Evaluation and treatment of sensory and perceptual impairments following stroke. *Topics in Geriatric Rehabilitation, 19*(2), 87-97.

Bouska, M., Kauffman, N., & Marcus, S. (1990). Disorders of the visual perceptual system. *Neurological rehabilitation*, 705-740.

Boyd, A., & Dawson, D. (2000). The relationship between perceptual impairment and self-care status in a sample of elderly persons. *Physical & Occupational Therapy in Geriatrics, 17*(4), 1-16. doi:10.1080/J48v17n04_01

Boys, M., Fisher, P., Holzberg, C., & Reid, D. W. (1988). The OSOT Perceptual Evaluation: A research perspective. *American Journal of Occupational Therapy, 42*(2), 92-98. doi:10.5014/ajot.42.2.92

Brown, G., Rodger, S., & Davis, A. (2003). Motor-free visual perception test—revised: An overview and critique. *British Journal of Occupational Therapy, 66*(4), 159-167. doi:10.1067/mem.2003.274

Brown, T., Mapleston, J., & Nairn, A. (2011). Convergent validity of the Occupational Therapy Adult Perceptual Screening Test (OT-APST) with two other cognitive-perceptual tests. *The British Journal of Occupational Therapy, 74*(12), 562-572. doi:10.4276/030802211X13232584581416

Brown, T., Mapleston, J., & Nairn, A. (2012). Can cognitive and perceptual standardized test scores predict functional performance in adults diagnosed with stroke? A pilot study. *Physical & Occupational Therapy in Geriatrics, 30*(1), 31-44. doi:10.3109/02703181.2011.652348

Brown, T., Mullins, E., & Stagnitti, K. (2008). The reliability of performance of healthy adults on three visual perception tests. *British Journal of Occupational Therapy, 71*(10), 438-447. doi:10.1177/030802260807101007

Butler, A., Cazeaux, J., Fidler, A., Jansen, J., Lefkove, N., Gregg, M., . . . Wolf, S. (2012). The Movement Imagery Questionnaire-Revised, Second Edition (MIQ-RS) is a reliable and valid tool for evaluating motor imagery in stroke populations. *Evidence-Based Complementary & Alternative Medicine: eCAM, 2012*, 497289. doi:10.1155/2012/497289

Buxbaum, L., Dawson, A., & Linsley, D. (2012). Reliability and validity of the Virtual Reality Lateralized Attention Test in assessing hemispatial neglect in right-hemisphere stroke. *Neuropsychology, 26*(4), 430. doi:10.1037/a0028674

Buxbaum, L., Palermo, M., Mastrogiovanni, D., Read, M., Rosenberg-Pitonyak, E., Rizzo, A., & Coslett, H. (2008). Assessment of spatial attention and neglect with a virtual wheelchair navigation task. *Journal of Clinical and Experimental Neuropsychology, 30*(6), 650-660. doi:10.1080/13803390701625821

Calvanio, R., Williams, R., Burke, D., Mello, J., Lepak, P., Al-Adawi, S., & Shah, M. (2004). Acquired brain injury, visual attention, and the useful field of view test: A pilot study. *Archives of Physical Medicine & Rehabilitation, 85*(3), 474-478. doi:10.1016/S0003-9993(03)00469-6

Cassidy, T., Bruce, D., Lewis, S., & Gray, C. (1994). The Association of visual field deficits and visuospatial neglect. *Age and Ageing, 23*(suppl_2), P21-b-P21. doi:10.1093/ageing/23.suppl_2.P21-b

Cate, Y., & Richards, L. (2000). Relationship between performance on tests of basic visual functions and visual-perceptual processing in persons after brain injury. *American Journal of Occupational Therapy, 54*(3), 326-334. doi:10.5014/ajot.54.3.326

Cermak, S., & Hausser, J. (1989). The Behavioral Inattention Test for unilateral visual neglect: A critical review. *Physical & Occupational Therapy in Geriatrics, 7*(3), 43-53. doi:10.1300/J148v07n03_04

Chen-Sea, M. (2000). Validating the Draw-A-Man Test as a personal neglect test. *American Journal of Occupational Therapy, 54*(4), 391-397. doi:10.5014/ajot.54.4.391

Chiu, E., Wu, W., Chou, C., Yu, M., & Hung, J. (2016). Test-retest reliability and minimal detectable change of the Test of Visual Perceptual Skills-Third Edition in patients with stroke. *Archives of Physical Medicine & Rehabilitation, 97*(11), 1917-1923. doi:10.1016/j.apmr.2016.04.023

Chiu, E., Yu, M., Wu, W., Chou, C., Hung, J., & Chen, P. (2019). Validation of the Test of Visual Perceptual Skills-Third Edition in patients with stroke. *Disability & Rehabilitation, 41*(1), 104-109. doi:10.1080/09638288.2017.1378389

Colarusso, R., & Hammill, D. (2003). *The Motor Free Visual Perception Test (MVPT-3)*. Navato, CA: Academic Therapy Publications.

Colarusso, R., & Hammill, D. (1972). *Motor-free visual perception test*. Academic Therapy Pub.

Collins, M., Kontos, A., Reynolds, E., Murawski, C., & Fu, F. (2014). A comprehensive, targeted approach to the clinical care of athletes following sport-related concussion. *Knee Surgery, Sports Traumatology, Arthroscopy, 22*(2), 235-246. doi:10.1007/s00167-013-2791-6

Cooke, D., McKenna, K., & Fleming, J. (2005). Development of a standardized occupational therapy screening tool for visual perception in adults. *Scandinavian Journal of Occupational Therapy, 12*(2), 59-71. doi:10.1080/11038120410020683-1

Cooke, D., McKenna, K., Fleming, J., & Darnell, R. (2005). The reliability of the Occupational Therapy Adult Perceptual Screening Test (OT-APST). *British Journal of Occupational Therapy, 68*(11), 509-517. doi:10.1177/03080226050681105

Cooke, D., McKenna, K., Fleming, J., & Darnell, R. (2006). Criterion validity of the Occupational Therapy Adult Perceptual Screening Test (OT-APST). *Scandinavian Journal of Occupational Therapy, 13*(1), 38-48. doi:10.1080/11038120500363006

D'Elia, L., Satz, P., Uchiyama, C., & White, T. (1996). *Color trails test*. PAR Odessa, FL.

Donnelly, S. (2002). The Rivermead Perceptual Assessment Battery: Can it predict functional performance? *Australian Occupational Therapy Journal, 49*(2), 71-81. doi:10.1046/j.1440-1630.2002.00308.x

Downing, D. (1986). *Line bisection.' A criterion related validity study.* Unpublished Master's Project. Department of Occupational Therapy. University of Florida. Gainesville, FL.

Dunlap, P., Mucha, A., Smithnosky, D., Whitney, S., Furman, J., Collins, M., . . . Sparto, P. J. (2018). The gaze stabilization test following concussion. *Journal of the American Academy of Audiology,* Advance online publication. doi:10.3766/jaaa.18015

Egelko, S., Gordon, W., Hibbard, M., Diller, L., Lieberman, A., Holliday, R., . . . Orazem, J. (1988). Relationship among CT scans, neurological exam, and neuropsychological test performance in right-brain-damaged stroke patients. *Journal of Clinical & Experimental Neuropsychology: Official Journal of the International Neuropsychological Society, 10*(5), 539-564. doi:10.1080/01688638808402794

Elkin-Frankston, S., Lebowitz, B., Kapust, L., Hollis, A., & O'Connor, M. (2007). The use of the Color Trails Test in the assessment of driver competence: Preliminary report of a culture-fair instrument. *Archives of Clinical Neuropsychology, 22*(5), 631-635. doi:10.1016/j.acn.2007.04.004

Erez, A., Katz, N., Ring, H., & Soroker, N. (2009). Assessment of spatial neglect using computerised feature and conjunction visual search tasks. *Neuropsychological Rehabilitation, 19*(5), 677-695. doi:10.1080/09602010802711160

Ferber, S., & Karnath, H. (2001). How to assess spatial neglect-line bisection or cancellation tasks? *Journal of Clinical and Experimental Neuropsychology, 23*(5), 599-607. doi:10.1076/jcen.23.5.599.1243

Figueiredo, S. (2011). Behavioral Inattention Test (BIT). Retrieved from <https://www.strokengine.ca/en/assess/bit/>

Fisher, P., Boys, M., & Holzberg, C. (1991). *The OSOT Perceptual evaluation manual: Revised.* Scarborough, Ont.: Nelson, Canada.

Fordell, H., Bodin, K., Bucht, G., & Malm, J. (2011). A virtual reality test battery for assessment and screening of spatial neglect. *Acta Neurologica Scandinavica, 123*(3), 167-174. doi:10.1111/j.1600-0404.2010.01390.x

Fullerton, K., McSherry, D., & Stout, R. (1986). Albert's test: A neglected test of perceptual neglect. *The Lancet, 327*(8478), 430-432. doi:10.1016/S0140-6736(86)92381-0

Galetta, K., Brandes, L., Maki, K., Dziemianowicz, M., Laudano, E., Allen, M., . . . Devick, S. (2011). The King–Devick test and sports-related concussion: study of a rapid visual screening tool in a collegiate cohort. *Journal of the Neurological Sciences, 309*(1-2), 34-39. doi:10.1016/j.jns.2011.07.039

Garzia, R., Richman, J., Nicholson, S., & Gaines, C. (1990). A new visual-verbal saccade test: The Developmental Eye Movement test (DEM). *Journal of the American Optometric Association*, 61(2), 124-135 .

George, S., Clark, M., & Crotty, M. (2008). Validation of the visual recognition slide test with stroke: A component of the New South Wales occupational therapy off-road driver rehabilitation program. *Australian Occupational Therapy Journal, 55*(3), 172-179. oi:10.1111/j.1440-1630.2007.00699.x

George, S., & Crotty, M. (2010). Establishing criterion validity of the useful field of view assessment and stroke drivers’ screening assessment: Comparison to the result of on-road assessment. *American Journal of Occupational Therapy, 64*(1), 114-122. doi:10.5014/ajot.64.1.114

Gordon, W., Ruckdeschel-Hibbard, M., Egelko, S., Diller, L., Simmens, S., Langer, K., . . . Weinberg, J. (1984). Single Letter Cancellation (Cancellation H) Test in evaluation of the deficits associated with right brain damage: normative data on the Institute of Rehabilitation Medicine Test Battery. *New York University Medical Center, Institute of Rehabilitation Medicine, New York*, 1-7.

Greve, K., Lindberg, R., Bianchini, K., & Adams, D. (2000). Construct validity and predictive value of the Hooper Visual Organization Test in stroke rehabilitation. *Applied Neuropsychology, 7*(4), 215-222. doi:10.1207/S15324826AN0704_3

Halligan, P., Wilson, B., & Cockburn, J. (1990). A short screening test for visual neglect in stroke patients. *International Disability Studies, 12*(3), 95-99. doi:10.3109/03790799009166260

Halligan, P., Cockburn, J., & Wilson, B. (1991). The behavioural assessment of visual neglect. *Neuropsychological Rehabilitation, 1*(1), 5-32. doi:10.1080/09602019108401377

Harlowe, D., & Van Deusen, J. (1984). Construct validation of the St. Marys CVA evaluation: Perceptual measures. *American Journal of Occupational Therapy, 38*(3), 184-186. doi:10.5014/ajot.38.3.184

Hartman-Maeir, A., Erez, A., Ratzon, N., Mattatia, T., & Weiss, P. (2008). The validity of the Color Trail Test in the pre-driver assessment of individuals with acquired brain injury. *Brain Injury, 22*(13/14), 994-998. doi:10.1080/02699050802491305

Hartman-Maeir, A., & Katz, N. (1995). Validity of the Behavioral Inattention Test (BIT): Relationships with functional tasks. *American Journal of Occupational Therapy, 49*(6), 507-516. doi:10.5014/ajot.49.6.507

Hunfalvay, M., Roberts, C., Murray, N., Tyagi, A., Kelly, H., & Bolte, T. (2019). Horizontal and vertical self-paced saccades as a diagnostic marker of traumatic brain injury. *Concussion, 4*(1), CNC60. doi:10.2217/cnc-2019-0001

Hunfalvay, M., Roberts, C., Murray, N., Tyagi, A., Barclay, K., Bolte, T., . . . Carrick, F. (2020). Vertical smooth pursuit as a diagnostic marker of traumatic brain injury. *Concussion, 5*(1), CNC69. doi:10.2217/cnc-2019-0013

Ishiai, S., Sugishita, M., Ichikawa, T., Gono, S., & Watabiki, S. (1993). Clock‐drawing test and unilateral spatial neglect. *Neurology, 43*(1 Part 1), 106-106. doi:10.1212/WNL.43.1_Part_1.106

Jolly, N., Macfarlane, A., & Heard, R. (2013). Towards gaining the best information about vision to assist the recovery of a patient with stroke. *Strabismus, 21*(2), 145-149. doi:10.3109/09273972.2013.787633

Katz, N., Hartman-Maeir, A., Ring, H., & Soroker, N. (1999). Functional disability and rehabilitation outcome in right hemisphere damaged patients with and without unilateral spatial neglect. *Archives of Physical Medicine & Rehabilitation, 80*(4), 379-384. doi:10.1016/S0003-9993(99)90273-3

Katz, N., Itzkovich, M., Averbuch, S., & Elazar, B. (1989). Loewenstein Occupational Therapy Cognitive Assessment (LOTCA) battery for brain-injured patients: Reliability and validity. *American Journal of Occupational Therapy, 43*(3), 184-192. doi:10.5014/ajot.43.3.184

Katz, N., Livni, L., Erez, A., & Averbuch, S. (2011). *Dynamic Loewenstein Occupational Therapy Cognitive Assessesment (DLOTCA)*. Pequannock, NJ: Maddak.

King, D., Brughelli, M., Hume, P., & Gissane, C. (2013). Concussions in amateur rugby union identified with the use of a rapid visual screening tool. *Journal of the Neurological Sciences, 326*(1-2), 59-63. doi:10.1016/j.jns.2013.01.012

Kinsella, G., Packer, S., Ng, K., Olver, J., & Stark, R. (1995). Continuing issues in the assessment of neglect. *Neuropsychological Rehabilitation, 5*(3), 239-258. doi:10.1080/09602019508401469

Koiava, N., Ong, Y., Brown, M., Acheson, J., Plant, G., & Leff, A. (2012). A 'web app' for diagnosing hemianopia. *Journal of Neurology, Neurosurgery & Psychiatry, 83*(12), 1222-1224. doi:10.1136/jnnp-2012-302270

Kontos, A., Sufrinko, A., Elbin, R., Puskar, A., & Collins, M. (2016). Reliability and associated risk factors for performance on the Vestibular/Ocular Motor Screening (VOMS) tool in healthy collegiate athletes. *The American Journal of Sports Medicine, 44*(6), 1400-1406. doi:10.1177/0363546516632754

Korner-Bitensky, N., Mazer, B., Sofer, S., Gelinas, I., Meyer, M., Morrison, C., . . . White, M. (2000). Visual testing for readiness to drive after stroke: A multicenter study. *American Journal of Physical Medicine & Rehabilitation, 79*(3), 253-317. doi:10.1097/00002060-200005000-00007

Kortman, B., & Nicholls, K. (2016). Assessing for unilateral spatial neglect using eye-tracking glasses: A feasibility study. *Occupational Therapy In Health Care, 30*(4), 344-355. doi:10.1080/07380577.2016.1208858

Làdavas, E. (1994). The role of visual attention in neglect: A dissociation between perceptual and directional motor neglect. *Neuropsychological Rehabilitation, 4*(2), 155-159. doi:10.1080/09602019408402275

Laukkanen, H., Scheiman, M., & Hayes, J. (2017). Brain Injury Vision Symptom Survey (BIVSS) questionnaire. *Optometry and Vision Science, 94*(1), 43-50. doi:10.1097/OPX.0000000000000940

Leibovitch, F., Vasquez, B., Ebert, P., Beresford, K., & Black, S. (2012). A short bedside battery for visuoconstructive hemispatial neglect: Sunnybrook Neglect Assessment Procedure (SNAP). *Journal of Clinical & Experimental Neuropsychology: Official Journal of the International Neuropsychological Society, 34*(4), 359-368. doi:10.1080/13803395.2011.645016

Lezak, M., Howieson, D., Loring, D., & Fischer, J. (2004). *Neuropsychological assessment*. Oxford University Press, USA.

Liu, K., Chan, C., Lee, T., & Hui-Chan, C. (2004). Mental imagery for relearning of people after brain injury. *Brain Injury, 18*(11), 1163-1172. doi:10.1080/02699050410001671883

Luukkainen-Markkula, R., Tarkka, I., Pitkanen, K., Sivenius, J., & Hamalainen, H. (2011). Comparison of the Behavioural Inattention Test and the Catherine Bergego Scale in assessment of hemispatial neglect. *Neuropsychological Rehabilitation, 21*(1), 103-116. doi:10.1080/09602011.2010.531619

Lyon, D., Goss, D., Horner, D., Downey, J., & Rainey, B. (2005). Normative data for modified Thorington phorias and prism bar vergences from the Benton-IU study. *Optometry-Journal of the American Optometric Association, 76*(10), 593-599. doi:10.1016/j.optm.2005.08.014

Malouin, F., Richards, C., Jackson, P., Lafleur, M., Durand, A., & Doyon, J. (2007). The Kinesthetic and Visual Imagery Questionnaire (KVIQ) for assessing motor imagery in persons with physical disabilities: A reliability and construct validity study. *Journal of Neurologic Physical Therapy, 31*(1), 20-29. doi:10.1097/NPT.0000260567.24122.64

Manos, P., & Wu, R. (1994). The ten point clock test: a quick screen and grading method for cognitive impairment in medical and surgical patients. *The International Journal of Psychiatry in Medicine, 24*(3), 229-244. doi:10.2190/5A0F-936P-VG8N-0F5R

Marsh, N., & Kersel, D. (1993). Screening tests for visual neglect following stroke. *Neuropsychological Rehabilitation, 3*(3), 245-257. doi:10.1080/09602019308401439

Marshall, S., Molnar, F., Man-Son-Hing, M., Blair, R., Finestone, H., Brosseau, L., . . . Wilson, K. (2007). Predictors of driving ability following stroke: A systematic review. *Topics in Stroke Rehabilitation, 14*(1), 98-114. doi:10.1310/tsr1401-98

Maruta, J., Suh, M., Niogi, S., Mukherjee, P., & Ghajar, J. (2010). Visual tracking synchronization as a metric for concussion screening. *The Journal of Head Trauma Rehabilitation, 25*(4), 293-305. doi:10.1097/HTR.0b013e3181e67936

Massironi, M., Antonucci, G., Pizzamiglio, L., Vitale, M., & Zoccolotti, P. (1988). The Wundt-Jastrow illusion in the study of spatial hemi-inattention. *Neuropsychologia, 26*(1), 161-166. doi:10.1016/0028-3932(88)90039-5

Matarazzo, J., Wiens, A., Matarazzo, R., & Goldstein, S. (1974). Psychometric and clinical test-retest reliability of the Halstead impairment index in a sample of healthy, young, normal men. *Journal of Nervous and Mental Disease*, 158(1), 37-49. doi:10.1097/00005053-197401000-00006

Matthey, S., Donnelly, S., & Hextell, D. (1993). The clinical usefulness of the Rivermead Perceptual Assessment Battery: Statistical considerations. *The British Journal of Occupational Therapy, 56*(10), 365-370. doi:10.1177/030802269305601003

Mattingley, J., Berberovic, N., Corben, L., Slavin, M., Nicholls, M., & Bradshaw, J. (2004). The greyscales task: A perceptual measure of attentional bias following unilateral hemispheric damage. *Neuropsychologia, 42*(3), 387-394. doi:10.1016/j.neuropsychologia.2003.07.007

Maxton, C., Dineen, R., Padamsey, R., & Munshi, S. (2013). Don't neglect 'neglect'- an update on post stroke neglect. *International Journal of Clinical Practice, 67*(4), 369-378. doi:10.1111/ijcp.12058

Mazer, B., Korner-Bitensky, N., & Sofer, S. (1998). Predicting ability to drive after stroke. *Archives of Physical Medicine and Rehabilitation, 79*(7), 743-757. doi:10.1016/S0003-9993(98)90350-1

Mazer, B., Sofer, S., Korner-Bitensky, N., & Gelinas, I. (2001). Use of the UFOV to evaluate and retrain visual attention skills in clients with stroke: A pilot study. *American Journal of Occupational Therapy, 55*(5), 552-557. doi:10.5014/ajot.55.5.552

Mazer, B., Sofer, S., Korner-Bitensky, N., Gelinas, I., Hanley, J., & Wood-Dauphinee, S. (2003). Effectiveness of a visual attention retraining program on the driving performance of clients with stroke. *Archives of Physical Medicine & Rehabilitation, 84*(4), 541-550. doi:10.1053/apmr.2003.50085

Mendez, M., Ala, T., & Underwood, K. (1992). Development of scoring criteria for the clock drawing task in Alzheimer's disease. *Journal of the American Geriatrics Society, 40*(11), 1095-1099. doi:10.1111/j.1532-5415.1992.tb01796.x

Messinis, L., Malegiannaki, A., Christodoulou, T., Panagiotopoulos, V., & Papathanasopoulos, P. (2011). Color Trails Test: Normative data and criterion validity for the Greek adult population. *Archives of Clinical Neuropsychology, 26*(4), 322-330. doi:10.1093/arclin/acr027

Moran, R., Covassin, T., Elbin, R., Gould, D., & Nogle, S. (2018). Reliability and normative reference values for the vestibular/ocular motor screening (VOMS) tool in youth athletes. *The American Journal of Sports Medicine, 46*(6), 1475-1480. doi:10.1177/0363546518756979

Mucha, A., Collins, M., Elbin, R., Furman, J., Troutman-Enseki, C., DeWolf, R.., . . . Kontos, A. (2014). A brief vestibular/ocular motor screening (VOMS) assessment to evaluate concussions: Preliminary findings. *The American Journal of Sports Medicine, 42*(10), 2479-2486. doi:10.1177/0363546514543775

Munoz, S., & Bangdiwala, S. (1997). Interpretation of Kappa and B statistics measures of agreement. *Journal of Applied Statistics, 24*(1), 105-112. doi:10.1080/02664769723918

Nijboer, T., Ten Brink, A., Kouwenhoven, M., & Visser-Meily, J. (2014). Functional assessment of region-specific neglect: Are there differential behavioural consequences of peripersonal versus extrapersonal neglect? *Behavioural Neurology, 2014*, 526407. doi:10.1155/2014/526407

O'donnell, J., Macgregor, L., Dabrowski, J., Oestreicher, J., & Romero, J. (1994). Construct validity of neuropsychological tests of conceptual and attentional abilities. *Journal of Clinical Psychology, 50*(4), 596-600. doi:10.1002/1097-4679(199407)50:4<596::AID-JCLP2270500416>3.0.CO;2-S

Ota, H., Fujii, T., Suzuki, K., Fukatsu, R., & Yamadori, A. (2001). Dissociation of body-centered and stimulus-centered representations in unilateral neglect. *Neurology, 57*(11), 2064-2069. doi:10.1212/WNL.57.11.2064

Piscicelli, C., Nadeau, S., Barra, J., & Pérennou, D. (2015). Assessing the visual vertical: How many trials are required? *BMC Neurology, 15*(1), 1-5. doi:10.1186/s12883-015-0462-6

Politzer, T., Berryman, A., Rasavage, K., Snel doi:10.1016/j.pmrj.2016.08.032

Potter, J., Deighton, T., Patel, M., Fairhurst, M., Guest, R., & Donnelly, N. (2000). Computer recording of standard tests of visual neglect in stroke patients. *Clinical Rehabilitation, 14*(4), 441-446. doi:10.1191/0269215500cr344oa

Punt, T., Kitadono, K., Hulleman, J., Humphreys, G., Riddoch, M., Punt, T., . . . Riddoch, M. (2008). From both sides now: Crossover effects influence navigation in patients with unilateral neglect. *Journal of Neurology, Neurosurgery & Psychiatry, 79*(4), 464-466. doi:10.1136/jnnp.2007.139832

Rainey, B., Schroeder, T., Goss, D., & Grosvenor, T. (1998). Inter-examiner repeatability of heterophoria tests. *Optometry and vision science: official publication of the American Academy of Optometry, 75*(10), 719-726. doi:10.1097/00006324-199810000-00016

Razemba, F., Jacobs, L., & Franzsen, D. (2017). Convergent validity of the Occupational Therapy Adult Perceptual Screening Test (OT-APST) with two other cognitive-perceptual tools in a South African context. *South African Journal of Occupational Therapy, 47*(2), 3-10. doi:10.17159/2310-3833/2017/v47n2a2

Reitan, R. (1955). The relation of the trail making test to organic brain damage. *Journal of Consulting Psychology, 19*(5), 393. doi:10.1037/h0044509

Robertson, L. (1993). Attentional search in unilateral visual neglect. In I. H. M. Robertson, J. C. (Ed.), *Unilateral neglect: Clinical and experimental studies* (pp. 169 –191). Hillsdale, NJ: Lawrence Erlbaum.

Rorden, C., Hjaltason, H., Fillmore, P., Fridriksson, J., Kjartansson, O., Magnusdottir, S., & Karnath, H. (2012). Allocentric neglect strongly associated with egocentric neglect. *Neuropsychologia, 50*(6), 1151-1157. doi:10.1016/j.neuropsychologia.2012.03.031

Rouse, M., Borsting, E., & Deland, P. (2002). Reliability of binocular vision measurements used in the classification of convergence insufficiency. *Optometry and Vision Science, 79*(4), 254-264.

Samuel, C., Louis-Dreyfus, A., Kaschel, R., Makiela, E., Troubat, M., Anselmi, N., . . . Azouvi, P. (2000). Rehabilitation of very severe unilateral neglect by visuo-spatio-motor cueing: Two single case studies. *Neuropsychological Rehabilitation, 10*(4), 385-399. doi:10.1080/096020100411970

Saviola, D., De Tanti, A., Conforti, J., Posteraro, L., Manfredini, A., Bagattini, C., & Basagni, B. (2018). Safe return to driving following severe acquired brain injury: Role of a short neuropsychological assessment. *European Journal of Physical & Rehabilitation Medicine., 54*(5), 717-723. doi:10.23736/S1973-9087.17.04905-X

Schatz, P. (2010). Long-term test-retest reliability of baseline cognitive assessments using ImPACT. *The American Journal of Sports Medicine, 38*(1), 47-53. doi:10.1177/0363546509343805

Schatz, P., Pardini, J., Lovell, M., Collins, M., & Podell, K. (2006). Sensitivity and specificity of the ImPACT Test Battery for concussion in athletes. *Archives of Clinical Neuropsychology, 21*(1), 91-99. doi:10.1016/j.acn.2005.08.001

Schenkenberg, T., Bradford, D., & Ajax, E. (1980). Line bisection and unilateral visual neglect in patients with neurologic impairment. *Neurology, 30*(5), 509-509. doi:10.1212/WNL.30.5.509

Schubert, F., & Spatt, J. (2001). Double dissociations between neglect tests: Possible relation to lesion site. *European Neurology, 45*(3), 160-164. doi:10.1159/000052115

Sea, M., & Henderson, A. (1994). The reliability and validity of visuospatial inattention tests with stroke patients. *Occupational Therapy International, 1*(1), 36-48. doi:10.1002/oti.6150010106

Stone, S., Wilson, B., Wroot, A., Halligan, P., Lange, L., Marshall, J., & Greenwood, R. (1991). The assessment of visuo-spatial neglect after acute stroke. *Journal of Neurology, Neurosurgery & Psychiatry, 54*(4), 345-350. doi:10.1136/jnnp.54.4.345

Su, C., Chien, T., Cheng, K., & Lin, Y. (1995). Performance of older adults with and without cerebrovascular accident on the test of visual-perceptual skills. *American Journal of Occupational Therapy, 49*(6), 491-499. doi:10.5014/ajot.49.6.491

Su, C., Chang, J., Chen, H., Su, C., Chien, T., & Huang, M. (2000). Perceptual differences between stroke patients with cerebral infarction and intracerebral hemorrhage. *Archives of Physical Medicine & Rehabilitation, 81*(6), 706-714. doi:10.1016/S0003-9993(00)90097-2

Tant, M., Brouwer, W., Cornelissen, F., & Kooijman, A. (2002). Driving and visuospatial performance in people with hemianopia. *Neuropsychological Rehabilitation, 12*(5), 419-437. doi:10.1080/09602010244000183

Tassinari, J., & DeLand, P. (2005). Developmental eye movement test: Reliability and symptomatology. *Optometry-Journal of the American Optometric Association, 76*(7), 387-399. doi:10.1016/j.optm.2005.05.006

Ten Brink, A., Visser-Meily, J., & Nijboer, T. (2018). Dynamic assessment of visual neglect: The mobility assessment course as a diagnostic tool. *Journal of Clinical and Experimental Neuropsychology, 40*(2), 161-172. doi:10.1080/13803395.2017.1324562

Tippett, W., Alexander, L., Rizkalla, M., Sergio, L., & Black, S. (2013). True functional ability of chronic stroke patients. *Journal of Neuroengineering & Rehabilitation, 10*, 20. doi:10.1186/1743-0003-10-20

Titus, M., Gall, N., Yerxa, E., Roberson, T., & Mack, W. (1991). Correlation of perceptual performance and activities of daily living in stroke patients. *American Journal of Occupational Therapy, 45*(5), 410-418. doi:10.514/ajot.45.5.410

Toglia, J., & Cermak, S. (2009). Dynamic assessment and prediction of learning potential in clients with unilateral neglect. *American Journal of Occupational Therapy, 63*(5), 569-579. doi:10.5014/ajot.63.5.569

Tsirlin, I., Dupierrix, E., Chokron, S., Coquillart, S., & Ohlmann, T. (2009). Uses of virtual reality for diagnosis, rehabilitation and study of unilateral spatial neglect: Review and analysis. *Cyberpsychology & Behavior, 12*(2), 175-181. doi:10.1089/cpb.2008.0208

Tuokko, H., Hadjistavropoulos, T., Miller, J., & Beattie, B. (1992). The Clock Test: A sensitive measure to differentiate normal elderly from those with Alzheimer disease. *Journal of the American Geriatrics Society, 40*(6), 579-584. doi:10.1111/j.1532-5415.1992.tb02106.x

Van der Stigchel, S., & Nijboer, T. (2018). Temporal order judgements as a sensitive measure of the spatial bias in patients with visuospatial neglect. *Journal of Neuropsychology, 12*(3), 427-441. doi:10.1111/jnp.12118

Van Deusen, J. (1988). Unilateral neglect: Suggestions for research by occupational therapists. *American Journal of Occupational Therapy, 42*(7), 441-448. doi:10.5014/ajot.42.7.441

Wang, Q., Sonoda, S., Hanamura, M., Okazaki, H., & Saitoh, E. (2005). Line bisection and rebisection: The crossover effect of space location. *Neurorehabilitation & Neural Repair, 19*(2), 84-92. doi:10.1177/1545968305274661

Warren, M. (1990). Identification of visual scanning deficits in adults after cerebrovascular accident. *American Journal of Occupational Therapy, 44*(5), 391-399. doi:10.5014/ajot.44.5.391

Weightman, M., Radomski, M., Mashima, P., & Roth, C. (2014). *Mild traumatic brain injury rehabilitation toolkit*: Borden Institute.

Weintraub, S. (2000). Neuropsychological assessment. *Principles of Behavioral and Cognitive Neurology, 121*.

Wetzel, P., Lindblad, A., Raizada, H., James, N., Mulatya, C., Kannan, M., . . . Weaver, L. (2018). Eye tracking results in postconcussive syndrome versus normative participants. *Investigative Ophthalmology & Visual Science, 59*(10), 4011-4019. doi:10.1167/iovs.18-23815

Whitehouse, C., Green, J., Giles, S., Rahman, R., Coolican, J., & Eskes, G. (2019). Development of the Halifax Visual Scanning Test: A new measure of visual-spatial neglect for personal, peripersonal, and extrapersonal space. *Journal of the International Neuropsychological Society*, 1-11. doi:10.1017/S135561771900002X

Whiting, S., Lincoln, N., Bhavnani, G., & Cockburn, J. (1986). Rivermead perceptual assessment battery. *Occupational Therapy in Health Care,* 3(3-4), 209-10. doi:10.1080/J003v03n03_18

Wilson, B., Cockburn, J., & Halligan, P. (1987). Development of a behavioral test of visuospatial neglect. *Archives of Physical Medicine & Rehabilitation, 68*(2), 98-102.

Worts, P., & Burkhart, S. (2019). Test performance and test-retest reliability of the vestibular/ocular motor screening and King-Devick Test in adolescent athletes during a competitive sport season: Response. *The American Journal of Sports Medicine, 47*(2), NP16-NP18. doi:10.1177/0363546518819445

Yaretzky, A., Raviv, S., Netz, Y., & Jacob, T. (1995). Primary visual memory of stroke patients. *Disability & Rehabilitation, 17*(6), 293-297. doi:10.3109/09638289509166649

York, C., & Cermak, S. (1995). Visual perception and praxis in adults after stroke. *American Journal of Occupational Therapy, 49*(6), 543-550. doi:10.5014/ajot.49.6.543

Zaninotto, A., Vicentini, J., Solla, D., Silva, T., Guirado, V., Feltrin, F., . . . Paiva, W. (2017). Visuospatial memory improvement in patients with diffuse axonal injury (DAI): A 1-year follow-up study. *Acta Neuropsychiatrica, 29*(1), 35-42. doi:10.1017/neu.2016.29

Zeltzer, L. (2008a). Motor-Free Visual Perception Test (MVPT). Retrieved from <https://www.strokengine.ca/en/assess/mvpt/>

Zeltzer, L. (2008b). Ontario Society of Occupational Therapists (OSOT) Perceptual Evaluation. Retrieved from <https://www.strokengine.ca/en/assess/osot/>

Zeltzer, L., & Menon, A. (2008a). Clock Drawing Test (CDT). Retrieved from <https://www.strokengine.ca/en/assess/cdt/>

Zeltzer, L., & Menon, A. (2008b). Draw-A-Man Test. Retrieved from <https://www.strokengine.ca/en/assess/damt/>

Zeltzer, L., & Menon, A. (2008c). Line Bisection Test. Retrieved from <https://www.strokengine.ca/en/assess/lbt/>

Zoccolotti, P., Antonucci, G., & Judica, A. (1992). Psychometric characteristics of two semi-structured scales for the functional evaluation of hemi-inattention in extrapersonal and personal space. *Neuropsychological Rehabilitation, 2*(3), 179-191. doi:10.1080/09602019208401407

Zoccolotti, P., Antonucci, G., Judica, A., Montenero, P., Pizzamiglio, L., & Razzano, C. (1989). Incidence and evolution of the hemineglect disorder in chronic patients with unilateral right brain damage. *International Journal of Neuroscience, 47*(3-4), 209-216. doi:10.3109/00207458908987435

Zoccolotti, P., Guariglia, C., Pizzamiglio, L., Judica, A., Razzano, C., & Pantano, P. (1992). Good recovery in visual scanning in a patient with persistent anosognosia. *International Journal of Neuroscience, 63*(1-2), 93-104. doi:10.3109/00207459208986659

Zoltan, B. (2007). *Vision, perception, and cognition: A manual for the evaluation and treatment of the adult with acquired brain injury, fourth edition (4^th^ ed.)*. Thorofare, NJ: Slack Incorporated.
